# Dendrimer-Functionalized Superparamagnetic Nanobeacons for Real-Time Detection and Depletion of HSP90α mRNA and MR Imaging

**DOI:** 10.7150/thno.36545

**Published:** 2019-08-12

**Authors:** Zhongyuan Chen, Yueting Peng, Xiaoxue Xie, Yi Feng, Tingting Li, Shun Li, Xiang Qin, Hong Yang, Chunhui Wu, Chuan Zheng, Jie Zhu, Fengming You, Yiyao Liu

**Affiliations:** 1 Department of Biophysics, School of Life Science and Technology, University of Electronic Science and Technology of China, Chengdu 610054, Sichuan, P.R. China; 2 Hospital of Chengdu University of Traditional Chinese Medicine, No. 39 Shi-er-qiao Road, Chengdu 610072, Sichuan, P.R. China; 3 Center for Information in Biology, University of Electronic Science and Technology of China, Chengdu 610054, Sichuan, P.R. China

**Keywords:** iron oxide nanocubes, poly(amidoamine) dendrimer, cancer theranostics, HSP90α, molecular beacon

## Abstract

**Background & Aims**: The use of antisense oligonucleotide-based nanosystems for the detection and regulation of tumor-related gene expression is thought to be a promising approach for cancer diagnostics and therapies. Herein, we report that a cubic-shaped iron oxide nanoparticle (IONC) core nanobeacon is capable of delivering an HSP90α mRNA-specific molecular beacon (HSP90-MB) into living cells and enhancing *T*_2_-weighted MR imaging in a tumor model.

**Methods**: The nanobeacons were built with IONC, generation 4 poly(amidoamine) dendrimer (G4 PAMAM), Pluronic P123 (P123) and HSP90-MB labeled with a quencher (BHQ1) and a fluorophore (Alexa Fluor 488).

**Results**: After internalization by malignant cells overexpressing HSP90α, the fluorescence of the nanobeacon was recovered, thus distinguishing cancer cells from normal cells. Meanwhile, MB-mRNA hybridization led to enzyme activity that degraded DNA/RNA hybrids and resulted in downregulation of HSP90α at both the mRNA and protein levels. Furthermore, the *T*_2_-weighted MR imaging ability of the nanobeacons was increased after PAMAM and P123 modification, which exhibited good biocompatibility and hemocompatibility.

**Conclusions**: The nanobeacons show promise for applicability to tumor-related mRNA detection, regulation and multiscale imaging in the fields of cancer diagnostics and therapeutics.

## Introduction

Heat shock proteins (HSPs) are a group of molecular chaperones that help proteins fold into their native conformation, and protect cells from stressful environments. However, certain HSPs are overexpressed by malignant cells to survive various therapies, inhibit cancer cells from undergoing senescence and apoptosis, stabilize the lysosome membrane and promote autophagy 1. Among the HSP family, HSP27, HSP60, HSP70 and HSP90 are the most well-known and most widely studied. Importantly, HSP90 inhibition sensitizes tumor cells to chemotherapy-induced death 2. Treatment with the HSP90 inhibitor (17-DMAG) promotes breast cancer stem cell necrosis in nanoparticle-based photothermal therapies 3. Therefore, the quantitative detection and downregulation of the HSP90 expression level are of great value for cancer diagnosis and therapy. Currently, quantitative real-time polymerase chain reaction (*q*RT-PCR) and microarray analysis are the standard methods for mRNA detection 4, which can reveal the average mRNA levels in cell lysates from a large number of cell groups rather than individual living cells 5. Recently, hairpin-structured molecular beacons (MBs) have found numerous applications in carcinogenesis studies and medical therapy due to their nucleic acid recognition and sensitive Förster resonance energy transfer (FRET) property 6. For example, Tan and co-workers designed label-free MBs for the selective detection of DNA, RNA, and protein in TMB buffer 7. MBs have also used to detect exosomal miR-21 *in situ* 8.

Antisense oligonucleotides were wieldy used to promote RNA cleavage and degradation by nuclease activation to regulate tumor-related gene expression 9 or to inhibit translation by occupancy-only mechanisms 10. However, RNA and DNA oligonucleotides, including MBs, are rapidly degraded under physiological conditions, and the negatively charged oligonucleotides are difficult to internalize into cancer cells; therefore, they have limited utility as therapeutic agents 11. Fortunately, the advent of nanotechnology has led to the development of different kinds of gene delivery systems, such as mesoporous silica nanoparticles 12, nanographene 13, PLGA-based nanoparticles 14, gold nanoparticles 15, and iron oxide nanoparticles. Such delivery systems are able to protect and transfer plasmid DNA 16, 17, oligonucleotides 18 and fluorescent probes 19 into living cells. For example, Choi et al. constructed nanoprobes from iron oxide nanoparticles (IONPs) and hairpin MBs to detect miR-200a in living cells 20; Yin et al. utilized PEI coated magnetic core-shell nanoparticles to deliver a heat-inducible gene vector that encodes TRAIL into mesenchymal stem cells 21. It has been reported that a CD44-targeting HSP72 depletion nanosystem was fabricated to selectively sensitize MDA-MB-231 cells to hyperthermia and enhance therapeutic efficacy with minimal side effects both *in vitro* and *in vivo* 22. Moreover, since IONPs are able to shorten the T2 relaxation time of water protons, they enhanced imaging contrast and sensitivity in MR imaging; therefore, various sizes and shapes for IONPs have been investigated as *T*_2_-weighted MR imaging contrast agents 23-27. Together with drug/gene delivery 28-30 and magnetic hyperthermia capacities, IONP-based nanosystems exhibit great potential for advancements in cancer diagnostics and therapies 31.

**Figure 1:**
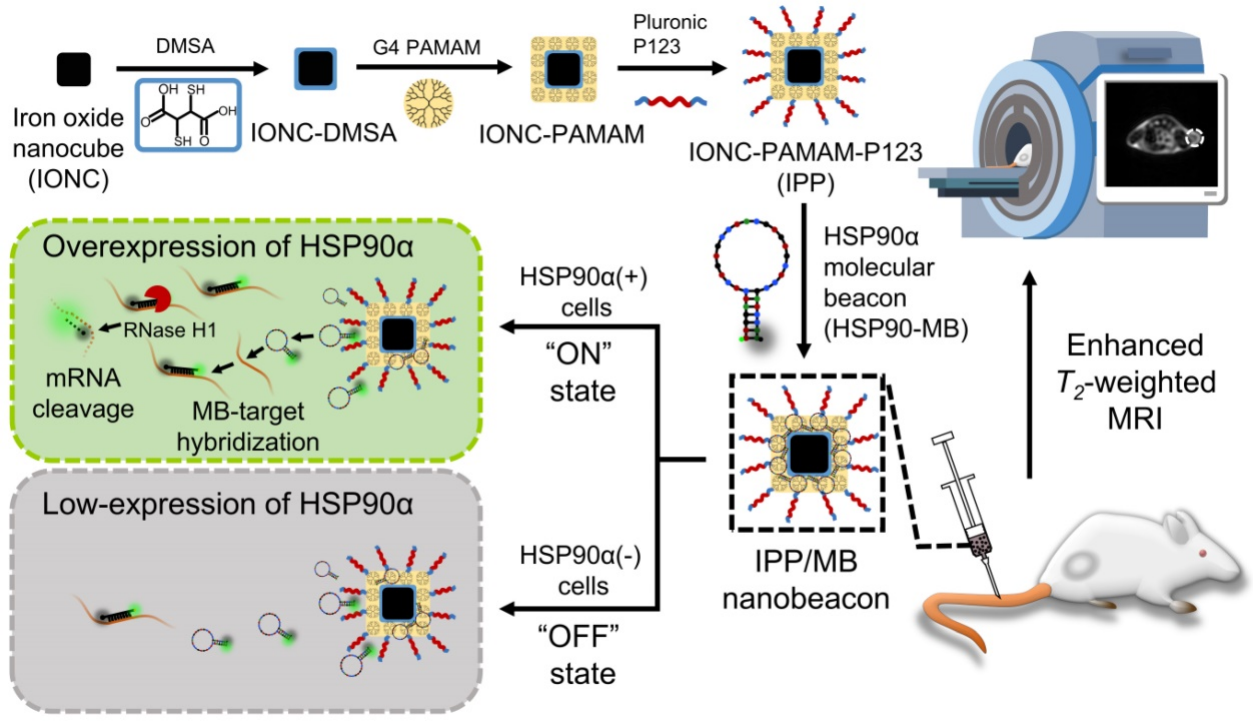
Schematic of the IONC-PAMAM-P123/HSP90α molecular beacon (IPP/MB nanobeacon) fabrication; the detection, imaging and downregulation of HSP90α mRNA in living cells, and enhanced *T*_2_-weighted MR imaging in a tumor model.

In this work, we designed and synthesized a new kind of nanobeacon, built with cubic-shaped iron oxide nanoparticle (IONC), generation 4 poly(amidoamine) dendrimer (G4 PAMAM), triblock copolymer Pluronic P123 (P123) and HSP90α mRNA-specific molecular beacon (HSP90-MB), to implement HSP90α mRNA quantitative detection and efficient downregulation in living cells and to enhance *T*_2_-weighted MR imaging in a tumor model (Figure 1). Thus, it might be a promising application for tumor-related mRNA detection, gene expression regulation and multiscale imaging in the field of theranostic cancer.

## Materials and Methods

### Materials

Iron(III) chloride hexahydrate (FeCl_3_·6H_2_O) was purchased from Chron Chemicals (Chengdu, China); oleic acid, sodium oleate (82% and 97%), 1-octadecene (>90%), meso-2,3-dimercaptosuccinic acid (DMSA), N-(3-dimethylaminopropyl)-N'-ethylcarbodiimide hydrochloride (EDC), N,N'-Carbonyldiimidazole (CDI), and N-hydroxysulfosuccinimide sodium salt (sulfo-NHS) were purchased from Aladdin (Shanghai, China); generation 4 poly(amidoamine) dendrimer (G4 PAMAM-NH_2_, 10% in methanol) was purchased from CY Dendrimer Technology (Weihai, China); Pluronic P123 (MW 5800) was purchased from Sigma-Aldrich (St. Louis, MO, USA); TRIzol reagent was purchased from Thermo Fisher Scientific (Waltham, MA, USA); PrimeScript RT Reagent Kit and SYBR Premix Ex Taq II (Tli RNaseH Plus) were purchased from Takara (Dalian, China); CellTiter 96 AQueous One Solution Cell Proliferation Assay was purchased from Promega (Fitchburg, WI, USA); LysoBlue was purchased from KeyGen BioTECH (Jiangsu, China); and 4S GelRed (10000X in water) and human GAPDH endogenous reference gene primers were purchased from BBI (Shanghai, China). All other oligonucleotides were synthesized and purified by Shanghai Sangon Biological Engineering Technology Co., Ltd. (Shanghai, China). All chemicals, if not mentioned, were of analytical grade and used as received without further purification. All aqueous solutions and dispersions were prepared using ultrapure water (≥18 MΩ, Milli-Q, Millipore, Bedford, MA, USA).

The MDA-MB-231 human breast cancer cell line was obtained from the American Type Culture Collection (ATCC), and cell culture products were purchased from GIBCO (Thermo Fisher Scientific, Waltham, MA, USA). The MCF-10A human breast non-tumorigenic cell line and its culture medium were purchased from Procell Life Science&Technology Co., Ltd. (Wuhan, China). An SPF severe immune-deficient strain (NCG) of female mice was purchased from GemPharmatech Co., Ltd (Jiangsu, China).

### Instruments

The 30,000 MWCO Amicon Ultra-4 Centrifugal Filter (Millipore, Bedford, MA, USA) was used to purify and concentrate nanoparticles during synthesis. The JEM-2010 transmission electron microscope (TEM) (JEOL, Tokyo, Japan) was used to characterize the sizes and shapes of nanoparticles. The size distribution, polydispersity index (PDI) and Zeta potential of nanoparticles were measured by dynamic light scattering (DLS) using a Zetasizer (NanoZS, Malvern Panalytical Instruments, UK). Crystalline structures were measured by X-ray diffraction (XRD) (X'Pert Pro, Malvern Panalytical Instruments, UK). Elemental iron analysis was performed on an ICP-ASE (ICP-OES 730, Agilent Technologies, Santa Clara, CA, USA). The magnetic properties of nanoparticles were measured using an MPMS3 Superconducting Quantum Interference Device (SQUID) magnetometer (Quantum Design, San Diego, CA, USA). Thermogravimetric analysis (TGA) was performed on a thermal analyzer (STA 449 F3, NETZSCH, Selb, Germany). IR spectra were recorded on an FT-IR spectrometer (Nicolet iS5, Thermo Fisher Scientific, Waltham, MA, USA). The *T_1_* and *T*_2_ relaxation time measurement and *T*_2_-weighted MR imaging were performed on a 3T MRI scanner (MR750; GE Discovery, Milwaukee, WI, USA) in the MRI research center, University of Electronic Science and Technology of China. A Fluorescence Microplate Reader (Varioskan Lux, Thermo Fisher Scientific, Waltham, MA, USA) was used to detect the fluorescence properties of nanobeacons. The proliferation assay and hemolysis assay results were recorded by a microplate reader (ELx808; BioTek Instruments, Winooski, VT, USA). Gel images were captured using a UV transilluminator (Bio-Rad, Philadelphia, PA, USA). *q*RT-PCR analysis was performed on a CFX96 Real-Time PCR Detection System (Bio-Rad, Philadelphia, PA, USA). Confocal microscopy was performed on a laser-scanning confocal microscopy system (LSM-800, Carl Zeiss, Oberkochen, Germany). Flow cytometry assays were performed on a Guava easyCyte flow cytometer (Millipore, Bedford, MA, USA).

### Synthesis and characterization of nanoparticles

Detailed synthesis routes of nanoparticles are shown in the Supporting Information. For characterization, the sizes and shapes of the IONC-OA, IONC-DMSA, IONC-PAMAM and IPP nanoparticles were determined by TEM. The size distributions of the IONC-OA nanoparticles were analyzed by measuring the edge length of the nanocubes. The crystalline structures of the IONC core were confirmed using the XRD pattern. The concentration of Fe in IONC-DMSA, IONC-PAMAM and IPP nanoparticles was determined by 10% nitric acid digestion at 70 °C and ICP-AES measurement with proper dilution. The hydrodynamic size distribution, PDI and Zeta potential of nanoparticles were measured by DLS in Milli-Q water (pH 7.0). Surface modification of the nanoparticles was characterized by FT-IR. To estimate the modification molecules on the particle surface and their dissociation behavior, TGA was performed from 30 °C to 600 °C at a heating rate of 10 °C min^-1^ under a flow of nitrogen (50 mL min^-1^). The magnetic properties of nanoparticles were measured using an MPMS3 SQUID magnetometer. Field-dependent magnetization curves were measured at 300 K as a function of the external field, ranging from -7 T to 7 T, and hysteresis curves were recorded. Temperature-dependent magnetization in a magnetic field of 60 O_e_ was recorded in the 5 K-300 K temperature range for zero-field-cooled (ZFC) and field-cooled (FC) samples.

### Synthesis and characterization of the HSP90AA molecular beacon

The sequence of the HSP90AA (Gene ID: 3320) molecular beacon (HSP90-MB), the primers of HSP90AA and the sequences of targeted or mismatched oligonucleotides were designed using Beacon Designer 8 software and verified by NCBI primer-BLAST. The potential secondary structures and the target recognition ability of HSP90-MB were predicted using NUPAK 36. The sequences of oligonucleotides are shown in Table S1 in the Supporting Information. The target concentrations for the dependent fluorescence recovery property of HSP90-MB was examined using MB-target hybridization experiment. HSP90-MB (64 nM) was incubated with increasing concentrations of the oligonucleotide targets (0-128 nM) in 0.01 M phosphate buffered saline (PBS) at 37 °C for 4 h, and the fluorescence spectrum (Ex = 495 nm) was recorded using a fluorescence microplate reader. The specificity of HSP90-MB was examined by incubating HSP90-MB (64 nM) with target, mismatched, or non-target sequences (0-128 nM) in 0.01 M PBS at 37 °C for 4 h and recording the fluorescence intensity (Ex = 495 nm, Em = 519 nm) by a fluorescence microplate reader.

### Preparation and characterization of the IPP/MB nanobeacon

To determine the optimal mass ratio of iron in IPP/HSP90-MB nanobeacon, increasing concentrations of IPP (0, 10, 20, 40, 80, 160, 320 μg Fe mL^-1^) were added to the HSP90-MB solution (10 μg mL^-1^) at equal volume. The mixture was vortexed briefly and incubated at 37 °C for 20 min to ensure complex formation. Agarose gel electrophoresis was performed to reveal the condensation ability of different mass ratios of iron in IPP to HSP90-MB 32.

To examine the oligonucleotides protection ability of IPP nanoparticles, an agarose gel electrophoresis assay of HSP90-MB protection was performed according to our previous study 16. Briefly, IPP/MB at the mass ratio of 16:1 (IPP (Ferrum) to HSP90-MB) was prepared as described above. 100 μL of samples (0.5 μg HSP90-MB) were incubated with 2 μL DNase I (1 U/μL) at 37 °C for 1 h. Samples were treated with 1 μL of heparin (8 U/μL) at 37 °C for 1 h to release the HSP90-MB. Samples were treated with 10 μL of 25mM EDTA (10X EDTA) and incubated at 65 °C for 10 min to inactivate DNase I. Agarose gel electrophoresis was performed to detect the degradation of HSP90-MB.

The particle size distribution, PDI and Zeta potential of the nanobeacon were measured by DLS after the samples were appropriately diluted in Milli-Q water (pH 7.0).

### MR relaxivity assay

To evaluate the longitudinal relaxivity (r_1_) and transverse relaxivity (r_2_) of the nanobeacon, 1 mL of aqueous dispersion containing increasing concentrations of nanobeacon (0.02-0.6 mM Fe) was pipetted into 2-mL PE tubes. *T*_1_ and *T*_2_ relaxation time measurement was performed by a clinical 3T MR imaging device in which the tubes were vertically placed inside an animal coil.

*T*_1_ relaxation time was measured using SE-IR sequence with increasing inversion times (TI = 50, 400, 1100 and 2500 ms) according to a previous study 33, and *T*_2_ mapping sequence was used to generate MR images with increasing echo times (TE = 10.072 to 80.576 ms) and a fixed repetition time (TR = 2000 ms). The *T*_2_ relaxation time was determined by the following formula 34:


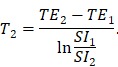


TE_1_ and TE_2_ are different echo times in a *T*_2_ mapping scan, SI_1_ and SI_2_ are the corresponding MR imaging signal intensities in the same region of the corresponding TE image.

The r_1_ and r_2_ relaxivity of each sample were determined by the linear fitting of 1/*T*_1_ and 1/*T*_2_ as a function of the Fe concentration.

### Cell culture

The TNBC cell line, MDA-MB-231, was cultured in Leibovitz's L-15 medium supplemented with 10% new-born calf serum and 1% penicillin-streptomycin at 37 °C in a humidified incubator. The non-tumorigenic epithelial cell line, MCF-10A, was cultured in a medium that contained 92.6% Dulbecco's modified Eagle medium (nutrient mixture F-12 (DMEM/F12), 5% horse serum, 1% penicillin-streptomycin, and 1.4% growth factors) at 37 °C in a 5% CO_2_ humidified incubator.

### Cellular uptake of the IPP/MB nanobeacon

The IPP/MB nanobeacon (>600 μg Fe mL^-1^, dispersed in Milli-Q water) was mixed with culture medium to a final concentration of 10 μg Fe mL^-1^. The concentration of HSP90-MB on the nanobeacon was 0.625 μg mL^-1^ (~54 nM), at a mass ratio of 16:1 (Fe to HSP90-MB). Cells that were seeded at a density of 80% in a 6-well plate and allowed to spread overnight were then incubated with the nanobeacon for another 24 h at 37 °C. The medium was then removed, and the cells were washed with PBS three times, detached by trypsin, centrifuged at 100 *g* for 5 min. The pellet was resuspended in 10% nitric acid, digested at 70 °C, and incubated for 24 h in a constant temperature shaker at 300 rpm. After centrifugation at 2000 *g* for 5 min, the supernatant from the digested cells was obtained to measure the concentration of Fe by ICP-AES with proper dilution.

### Cytotoxicity assay

Cells were seeded in 96-well plates at a density of 80%, allowed to spread overnight, and then incubated with increasing concentrations of IONC-DMSA, PAMAM, IPP nanoparticles or the IPP/MB nanobeacon for another 48 h at 37 °C. A cell proliferation assay was performed using a CellTiter 96 AQueous. The absorbance at 490 nm was recorded by a microplate reader.

### Hemolysis assay

Red blood cells (RBCs) were isolated from fresh rabbit blood by centrifugation at 400 *g* for 10 min. The cells were purified by rinsing five times with PBS. The suspension of RBCs was diluted ten times with PBS and 0.1 mL was added to 0.9 mL of water (positive control), saline (negative control), and saline containing various concentrations of IONC-DMSA, PAMAM, IPP or the IPP/MB nanobeacon. After a gentle shaking, the mixtures were kept still for 2 h at 37 °C. After centrifugation of the mixture (9000 *g*, 1 min), the supernatant (hemoglobin) was transferred to 96-well plates, and the absorbance at 540 nm was recorded by a microplate reader. The percentage hemolysis was calculated using the following equation:


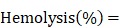



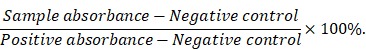


### Quantitative real-time RT-PCR for HSP90α mRNA

Approximately 1.0×10^6^ cells were collected for total RNA isolation using TRIzol reagent. Then, 2 μg of total RNA was reverse transcribed to cDNA using the PrimeScript™ RT Reagent Kit. Afterward, 2 μL of cDNA was analyzed by *q*RT-PCR targeting HSP90AA and GAPDH using SYBR Premix Ex Taq II (Tli RNaseH Plus). The analysis was performed on a CFX96 Real-Time System, with relative HSP90α mRNA expression levels determined by the ^ΔΔ^*C_t_* method using Bio-Rad CFX Manager software. The primers used in the *q*RT-PCR assay are shown in Table S1 in the Supporting Information.

### Western blot analysis for HSP90α

Western blot analysis was performed as previously described 35. Briefly, cells lysed in RIPA lysis buffer (Beyotime, China), after being washed in cold PBS, were separated by SDS-PAGE and transferred to PVDF membranes (Millipore, USA). The membranes were blocked with 5% non-fat dry milk in TBST buffer (10 mM Tris-HCl, 100 mM NaCl, and 0.1% Tween-20) for 1 h at room temperature before incubation with HSP90α (Abcam, mouse mAb; 1:1000) and β-actin (Abcam, mouse mAb; 1:1000) primary antibodies. After being washed to remove non-bound primary antibodies, the membranes were incubated with the corresponding secondary antibody at a 1:2000-10000 dilution at room temperature for 2 h. The membranes were washed three times with TBST, and immunoreactive signals were detected using western blot Luminol Reagent (Beyotime, China), according to the manufacturer's instructions.

### Confocal microscopy assay

MDA-MB-231 or MCF-10A cells were seeded in glass-bottom culture dishes and allowed to spread overnight. For subcellular localization assays, cells were incubated with the IPP/MB nanobeacon (10 μg Fe mL^-1^) for 4 h/24 h/48 h, then washed with PBS several times to remove excess nanobeacon. LysoBlue was used to detect acidic organelles such as lysosomes. For immunofluorescence staining, cells were incubated with the nanobeacon (10 μg Fe mL^-1^) for 24 h. They were then washed with PBS several times to remove the excess nanobeacon and fixed by 4% paraformaldehyde for 15 min at room temperature. Then, cells were permeabilized in 0.1% Triton X-100 for 10 min and blocked in 5% BSA for 1 h at 37 °C. The cells were incubated with HSP90α antibody (Abcam, mouse mAb; 1:100) at 4 °C overnight. Then, cells were incubated with secondary antibody coupled to Alexa Flour 647 for 1 h at 37 °C and washed with PBS before the cell nuclei were stained with DAPI. Finally, the confocal fluorescence images of the cells were obtained on a laser-scanning confocal microscopy system.

### Flow cytometry assay

MDA-MB-231 or MCF-10A cells were seeded at a density of 80% in a 6-well plate and allowed to spread overnight. Then, cells were incubated with the nanobeacon (10 μg Fe mL^-1^) for 24 h. The medium was removed, and the cells were washed with PBS several times to remove excess nanobeacon and detached by trypsin. The excess trypsin was removed by careful pipetting, and the cell monolayer was gently washed with PBS to collect the detached cells. Flow cytometry was performed on a Guava easyCyte flow cytometer using the green fluorescent channel (488 nm excitation) to evaluate the fluorescent recovery of the IPP/MB nanobeacon.

### *In vivo* T_2_-weighted MR imaging of a tumor model

All animal experiments were performed in agreement with the guidelines of the Institutional Animal Care Committee of the University of Electronic Science and Technology of China (UESTC). The MDA-MB-231 tumor model was established in female 4- to 6-week-old NCG mice by subcutaneously injecting 1.0×10^7^ MDA-MB-231 cells in 100 μL of saline-matrigel mixture (v:v = 1:1) into the right flank region. When tumors reached approximately 4-6 mm in diameter, the mice were randomly divided into two groups: saline or IPP/MB. A total of 100 μL of saline or IPP/MB (30 μg Fe mL^-1^, in 100 μL saline) was intravenously injected via the tail vein. *T*_2_-weighted MR images were obtained before injection and at time points 2, 4, 6, and 24 h postinjection by a clinical 3T MR imaging system with an animal coil. The mice were anesthetized with 2.5% isoflurane in air at a flow rate of 0.3 L min^-1^ and placed inside the animal coil. 3D FSE Cube *T*_2_-weighted MR images were obtained with a 2-mm slice thickness, 2000/59.568 ms TR/TE, 12×12 cm FOV, and a 128×128 matrix.

### *In vivo* biocompatibility of the nanobeacon

To evaluate the *in vivo* biocompatibility of the nanobeacon, at 72 h after intravenous injection of the IPP/MB nanobeacon (30 μg Fe mL^-1^, in 100 μL saline) or saline (100 μL), blood samples were collected from all groups of mice for a complete blood count analysis (red blood cells, white blood cells, platelets, mean platelet volume, hematocrit, hemoglobin, mean corpuscular volume, the percentage of intermediate cells, and mean corpuscular hemoglobin concentration). Afterward, all the mice were sacrificed, and the main organs (heart, liver, spleen, lungs, and kidneys) and tumors were collected in 10% neutral formalin. For H&E staining, paraffin-embedded tumor sections were stained and then observed and captured under a bright-field microscope with a digital camera (BA200 Digital, MOTIC, China).

### Statistical analysis

Each experiment was performed at least three times. All of the data were expressed as the means ± SEM using GraphPad Prism software, version 6.0. Statistical analyses were performed using the *t*-test or one-way analysis of variance with post hoc multiple comparisons. Significant differences between groups were considered at a minimum value of P<0.05.

## Results and discussion

### Design and characterization of the HSP90α molecular beacon

**Figure 2:**
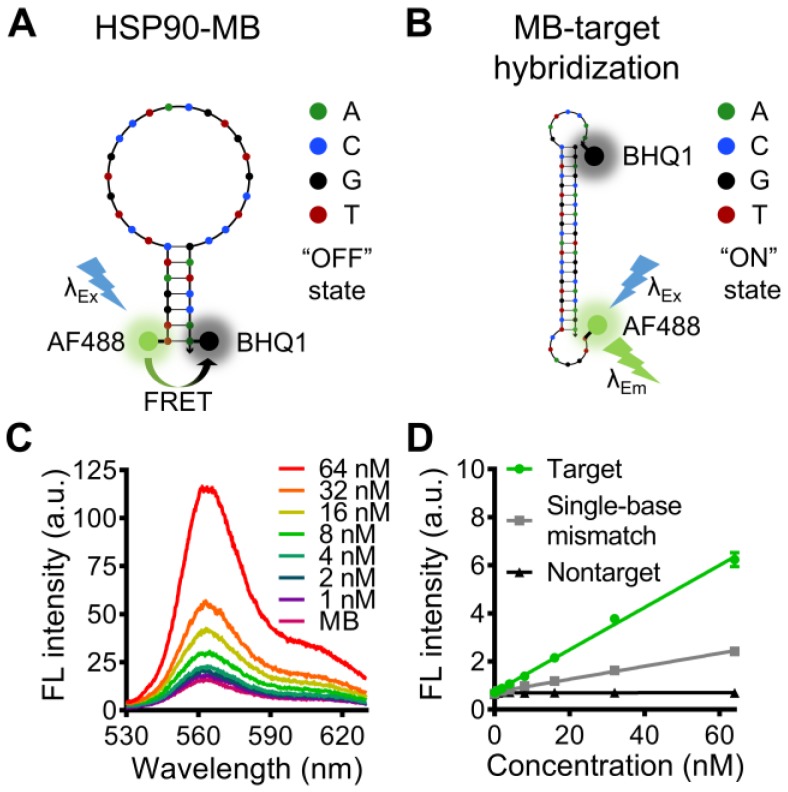
** Design and characterization of the HSP90α mRNA-specific molecular beacon. (A, B)** Potential secondary structures and target sequence recognition of the HSP90α mRNA-specific molecular beacon. **(C)** Target concentration-dependent fluorescence recovery of the HSP90AA molecular beacon was examined by beacon-target hybridization experiments, **(D)** the specificity of the molecular beacon was examined by beacon-target/single-base mismatch/nontarget hybridization experiments. The data are presented as the mean ± SEM.

The potential secondary structures and the target sequence recognition ability of the HSP90α mRNA- specific molecular beacon (HSP90-MB) were predicted using NUPAK 36. NUPAK indicated that, without target sequence, the HSP90-MB was likely to form a “stem and loop” conformation under physiological conditions ([Na^+^] = 154mM), and unfold to a more stable conformation after molecular beacon- target sequence hybridization (Figure 2A-B and Figure S1A-E).

A 5'- fluorophore (Alexa Fluor 488) and a 3'- quencher (BHQ1) were modified to the 34 bases oligonucleotide (Table S1), and the distance between Alexa Fluor 488 and BHQ1 was close enough to cause Förster resonance energy transfer (FRET) when the HSP90-MB was at “stem and loop” conformation. In this case, fluorescence of Alexa Fluor 488 was quenched by BHQ1, and the molecular beacon was at “OFF” state (fluorescence quenched) (Figure 2A). While with target sequence, the “loop” part of HSP90-MB could recognize and hybridize with target sequence through base-pairing interaction, and the “stem” part of HSP90-MB could be separated. In this case, the distance between Alexa Fluor 488 and BHQ1 was increased, and the fluorescence of Alexa Fluor 488 was recovered, the molecular beacon was at “ON” state (fluorescence recovered) (Figure 2B).

In addition, the free energy of secondary structure of those two conformations was calculated with NUPAK, and the result showed a -23.38 kcal/mol energy difference between “ON” and “OFF” states of HSP90-MB.

To verify the ability of HSP90-MB to quantitatively recognize target sequence, an MB-target hybridization experiment with increasing concentrations of the oligonucleotide targets was performed. The fluorescence intensity of HSP90-MB increased in a target concentration-dependent manner after MB-target hybridization (Figure 2C). To examine the specificity of HSP90-MB, increasing concentrations of target, mismatched or non-target sequences were incubated with HSP90-MB. The recovery of HSP90-MB fluorescence intensity was more significant in MB-target hybridization than in mismatched or non-target groups (Figure 2D). These results indicated that, the fluorescence intensity of HSP90-MB was specific to target sequence and in a target concentration-dependent manner.

### Synthesis and characterization of the IPP/MB nanobeacon

**Figure 3:**
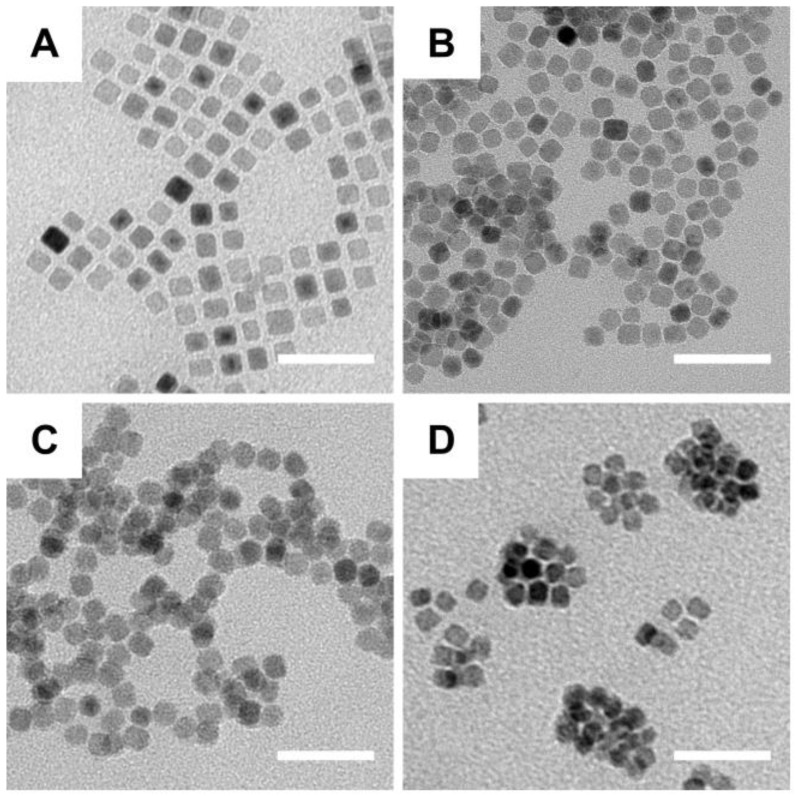
** TEM images of the fabrication of nanobeacons. (A)** IONC-OA, **(B)** IONC-DMSA (ID), **(C)** IONC-PAMAM (IP), and **(D)** IONC-PAMAM-P123 (IPP). Scale bar = 50 nm.

Synthesis of the IPP/MB nanobeacon was mainly divided into the following steps: preparation of the iron oleate precursor, synthesis and purification of iron oxide nanocubes 37, aqueous phase transfer 38, PAMAM conjugation 39, surface modification with Pluronic P123 40, and electrostatic interaction between nanocubes and HSP90-MB. Briefly, the oleate acid coated iron oxide nanocube (IONC-OA) was synthesized by thermal decomposition of iron oleate precursor with surfactants (oleic acid and sodium oleate) in a high-boiling solvent (1-octadecene, B.P. = 315 °C). The thermal decomposition process was controlled by a home-made PID controller based on Arduino (Figure S2). IONC-OA was well dispersed in n-hexane without aggregation. TEM images showed the uniform cube shape of IONC-OA with an edge length of 10.7±1.0 nm (Figure 3A).

**Figure 4:**
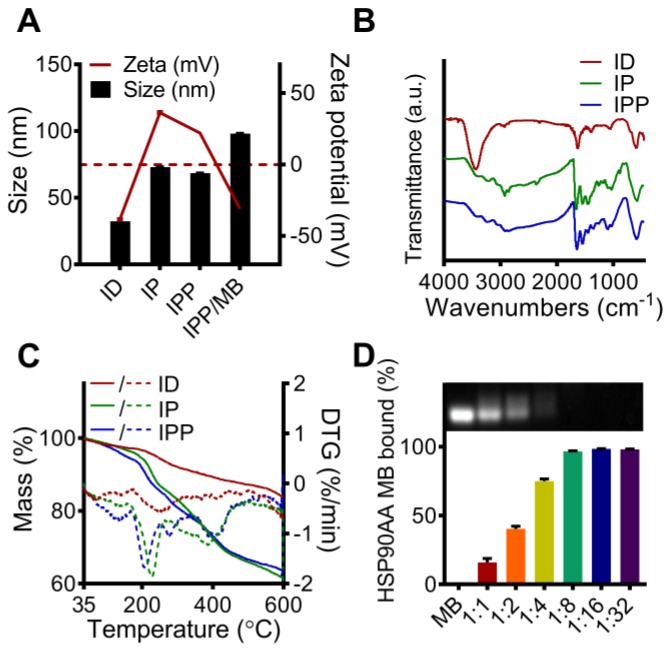
** Characterization of the ID, IP, IPP and IPP/MB nanobeacon. (A)** DLS analysis of the size and Zeta potential of ID, IP, IPP and IPP/MBs. The data are presented as the mean ± SEM. **(B)** IR spectra of ID, IP and IPP. **(C)** TG and DTG analysis of ID, IP, and IPP nanoparticles. Solid curves for mass losses; dashed curves for DTGs. **(D)** Condensation ability and mass ratio of IPP to MB was determined by agarose gel electrophoresis, and the quantitative assay was performed by measuring the intensity of agarose gel electrophoresis images using ImageJ software. The data are presented as the mean ± SEM.

After ligand-exchange with DMSA, thiol and carboxyl groups were anchored to the surface of the nanocubes (IONC-DMSA, denoted as ID), which provided aqueous dispersibility and sites for further conjugation. In addition, IONC-DMSA dispersed less stably in acid solution (pH < 3.0) (Figure S4), which may be due to neutralization of the carboxylate groups under acidic conditions, thus weakening the repulsion of negative charges from surface carboxyl groups 41. PAMAM was conjugated to IONC-DMSA via EDC/sulfo-NHS chemistry. The activation of carboxyl groups was performed in DMSO to avoid particle aggregation, after which IONC-DMSA was added dropwise to PAMAM in PBS (pH 7.4). The successful crosslinking between PAMAM and IONC-DMSA (IONC-PAMAM, denoted as IP) was indicated by a Zeta potential reversal from -40 mV to +40 mV (Figure 4A) and amide bond formation was confirmed by FT-IR (Figure 4B). Interestingly, IONC-PAMAM disperses better in acidic solutions than in alkaline solutions (*e.g.,* pH 10.0) due to the high density of surface amino groups on PAMAM (Figure S4). The high surface charge limited the application of IONC-PAMAM and could be improved via Pluronic P123 decoration 42. The successful fabrication of IONC-PAMAM-P123 (denoted as IPP) was indicated by a decrease in Zeta potential to +20 mV (Figure 4A) and confirmed by FT-IR (Figure 4B). Furthermore, IPP was fairly stable over wide ranges of pH 3.0-10.0 (Figure S4). In addition, thermogravimetric analysis (TGA) was performed to estimate the number of modification molecules on the surface of nanocubes; the DTG peaks of ID, IP and IPP increased in a synthetic procedure manner (Figure 4C). The XRD patterns of ID, IP and IPP were measured and confirmed the IONC core had the same crystalline structure as synthetic magnetite (Fe^2+^Fe^3+^_2_O_4_, PDF#19-0629) (Figure S3).

The IPP/MB nanobeacon was constructed by electrostatic interaction between the positively charged of PAMAM inside IPP and the negatively charged HSP90-MB at the appropriate mass ratio. The optimal mass ratio of iron in IPP to HSP90-MB was determined to be 16:1 by agarose gel electrophoresis (Figure 4D), in which HSP90-MB was completely condensed into the IPP. Under this mass ratio, approximately 16~17 HSP90-MBs was condensed into each IPP. The calculation of the HSP90-MB content in IPP/MB nanobeacons was in Supporting Information.

As cationic polymer, PAMAM can from complexes with oligonucleotides 43, and Pluronic P123 decoration can inhibit nonspecific protein adsorption by forming a non-ionic hydrophilic shell 40, thus may protect HSP90-MB from degradation under physiological conditions. To examine the oligonucleotides protection ability of IPP nanoparticles, another agarose gel electrophoresis assay was performed according to our previous study 16, and the result showed IPP nanoparticles could protect HSP90-MB from DNase I degradation at the mass ratio of 16:1 (Figure S5).

**Figure 5:**
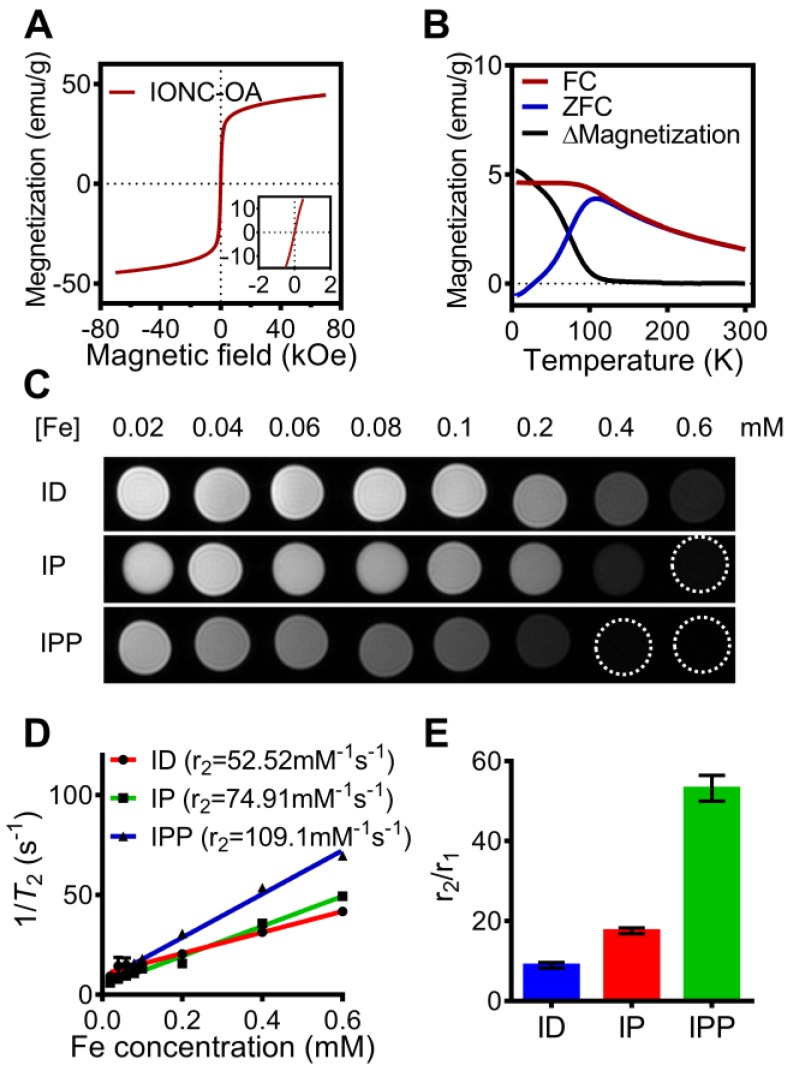
** Magnetic property of IONC-OA and *T*_2_-weighted MR imaging of ID, IP and IPP. (A, B)** Magnetic hysteresis and FC/ZFC assay confirmed the superparamagnetic property of the IONC core above 200K. **(C, D)** *T*_2_-weighted MR imaging and transverse relaxivity (r_2_) of ID, IP and IPP was examined by a clinical 3T MR imaging device with a *T*_2_ mapping sequence. The data are presented as the mean ± SEM. **(E)** r_2_/r_1_ ratio of ID, IP and IPP. The data are presented as the mean ± SEM.

To examine the magnetic property of the nanobeacon, field and temperature dependent magnetization curves of IONC-OA were recorded. The hysteresis curves showed superparamagnetism of the nanocubes in the range from -7 T to 7 T (Figure 5A), while the zero-field-cooled (ZFC) and field-cooled (FC) curves confirmed the superparamagnetic property appears at above 200 K (Figure 5B).

According to a study of Kim, et al. 44, the size of iron oxide nanoparticles have significant influences on both longitudinal relaxivity (r_1_) and transverse relaxivity (r_2_). In a range of 2.2 to 12 nm, the r_1_ relaxivity was decreased while the r_2_ relaxivity was increased as the size of iron oxide nanoparticles increases. To further investigate the capability of nanobeacon as an MRI contrast agent, *T*_1_ and *T*_2_ relaxation time measurement of ID, IP, and IPP were performed by a clinical 3T MR imaging device (Figure S6A and Figure 5C), and the r_1_ relaxivity and r_2_ relaxivity were determined by the linear fitting of 1/*T*_1_ and 1/*T*_2_ as a function of the Fe concentration (Figure S6B and Figure 5D).

Interestingly, r_2_ increased and r_1_ decreased after PAMAM conjugation and surface modification with Pluronic P123. As a result, IPP showed not only the highest r_2_ value of 109.1 mM^-1^s^-1^ (Figure 5D), but also the highest r_2_/r_1_ ratio of 53.19 (Figure 5E). Those results indicated the potential of IPP as a *T*_2_-weighted MRI contrast agent. It could be that after PAMAM conjugation and Pluronic P123 modification, the iron oxide core becomes more efficient at dephasing the spins of surrounding water protons, thus enhancing *T*_2_ relaxation times 45.

### *In vitro* cytotoxicity and hemolytic analysis of the IPP/MB nanobeacon

**Figure 6:**
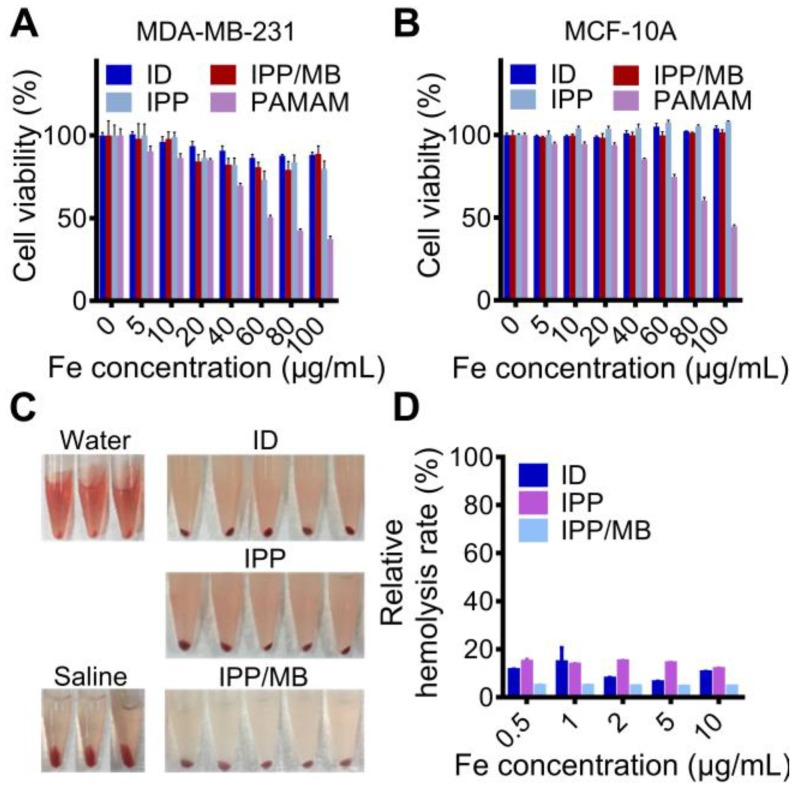
** Cytotoxicity and hemolytic analysis of the nanobeacon. (A, B)** Cell proliferation assay showed that cell viability was not compromised in neither cancerous nor normal cell lines after incubations with IONC-DMSA, PAMAM, IPP, and the IPP/MB nanobeacon for 48 h. The data are presented as the mean ± SEM. **(C, D)** Hemolytic assay indicated the nanobeacons have good hemocompatibility. The data are presented as the mean ± SEM.

The cytotoxicity of IONC-DMSA, PAMAM, IPP, and the IPP/MB nanobeacon were examined with a cell proliferation assay performed on cancer (MDA- MB-231) and normal (MCF-10A) cell lines (Figure 6A-B) 46. The results indicated the IPP/MB nanobeacon had good cytocompatibility (cell viability > 80%) at concentrations up to 100 μg Fe mL^-1^ in both cell lines. Next, the hemocompatibility of IONC-DMSA, IPP, and the IPP/MB nanobeacon were evaluated with the hemolysis assay (Figure 6C-D). The IPP/MB nanobeacon clearly did not show appreciable hemolytic activity at concentrations up to 10 μg Fe mL^-1^, with a corresponding hemolytic percentage of 4.63%. These results reveal that the IPP/MB nanobeacon showed negligible hemolytic activity and cytotoxicity in a concentration range of 0.5-10 μg Fe mL^-1^. Therefore, it was deemed suitable for further biomedical applications.

### Real-time quantitative detection and imaging of HSP90α mRNA* in vitro*

The IPP/MB nanobeacon was incubated with the MDA-MB-231 or MCF-10A cell line to verify cellular uptake behavior. The results showed that MDA-MB- 231 cells uptake 1.8-fold more nanobeacon than MCF-10A cells (Figure S7), which was consistent with previous studies 22, 47.

**Figure 7:**
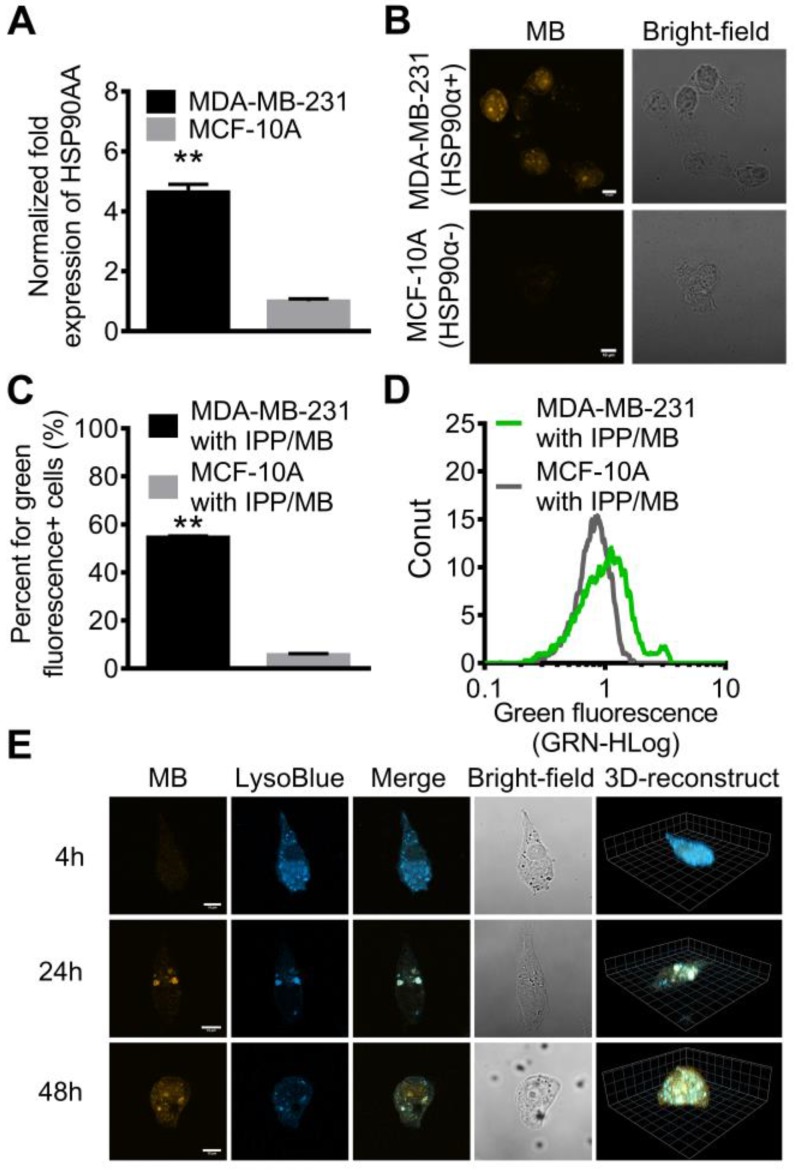
** Real-time quantitative detection and imaging of HSP90 mRNA *in vitro*. (A)** Different expression levels of HSP90AA in MDA-MB-231 and MCF-10A cell lines was confirmed by *q*RT-PCR. The data are presented as the mean ± SEM. **P<0.01. **(B)** CLSM images showed that high expression of HSP90 mRNA in MDA-MB-231 cells was detected by the nanobeacons while MCF-10A cells remained dark. Scale bar = 10 μm. **(C, D)** The fluorescence properties of the MDA-MB-231 and MCF-10A cell lines after incubating with nanobeacons were further examined by flow cytometry. The data are presented as the mean ± SEM. **P<0.01. **(E)** LSCM and 3D reconstructed images of MDA-MB-231 cells incubated with the nanobeacon for 4 h, 24 h and 48 h showed the intracellular location and distribution of the nanobeacon. Scale bar = 10 μm.

Since certain HSPs (such as HSP90) are overexpressed by malignant cells rather than normal cells, *q*RT-PCR analysis for HSP90AA mRNA level in the two cell lines was performed, which confirmed that the HSP90AA expression level in MDA-MB-231 cells was 4.7-fold higher than that in MCF-10A cells (Figure 7A).

Based on those findings, IPP nanoparticle was used as non-viral oligonucleotide delivery vector to transfer HSP90-MB into MDA-MB-231 or MCF-10A cells, to examine the HSP90α mRNA quantitative detection property of HSP90-MB in living cells.

Confocal microscopy was used to detect and visualize IPP/MB nanobeacon uptake and HSP90AA mRNA expression in the two cell lines. After a 48-h incubation, the fluorescence of Alexa Fluor 488 (labeled at the 5' end of HSP90-MB) was observed via confocal microscopy in MDA-MB-231 cells, while MCF-10A cells remained dark, which indicated that the IPP/MB nanobeacon was sensitive and specific enough to distinguish the HSP90α mRNA levels between MDA-MB-231 and MCF-10A *in vitro* (Figure 7B).

Further examination of the subcellular localization of the IPP/MB nanobeacon showed the nanobeacon was first located endosomes and lysosomes at 24 h and escaped to the cytoplasm after a 48-h incubation (Figure 7E and Figure S8). The endosomal/lysosomal escape of the IPP/MB nanobeacon could be explained by the proton sponge mechanism of the PAMAM dendrimer 48. Quantitative analysis for fluorescence of the IPP/MB nanobeacon in the two cell lines was performed by flow cytometry, which showed that green fluorescent (IPP/MB-positive) cells were 9.6-fold more abundant in the MDA-MB-231 line (Figure 7C-D). In consideration of the different cellular uptake profiles of these two cell lines, there was still 5.3-fold more positive cells in the MDA-MB-231 line than in MCF-10A, consistent with the *q*RT-PCR results. Taken together, these results suggested that the IPP/MB nanobeacon was capable of quantitative detection and imaging of mRNA expression levels in living cells. The fluorescence of nanobeacon is “ON” at cells that overexpression of HSP90α and “OFF” at HSP90α low-expression cells. Therefore, it is suitable for visualization of the difference between cancer and normal cells *in vitro*.

### Regulating intracellular mRNA and protein levels of HSP90α *in vitro*

**Figure 8:**
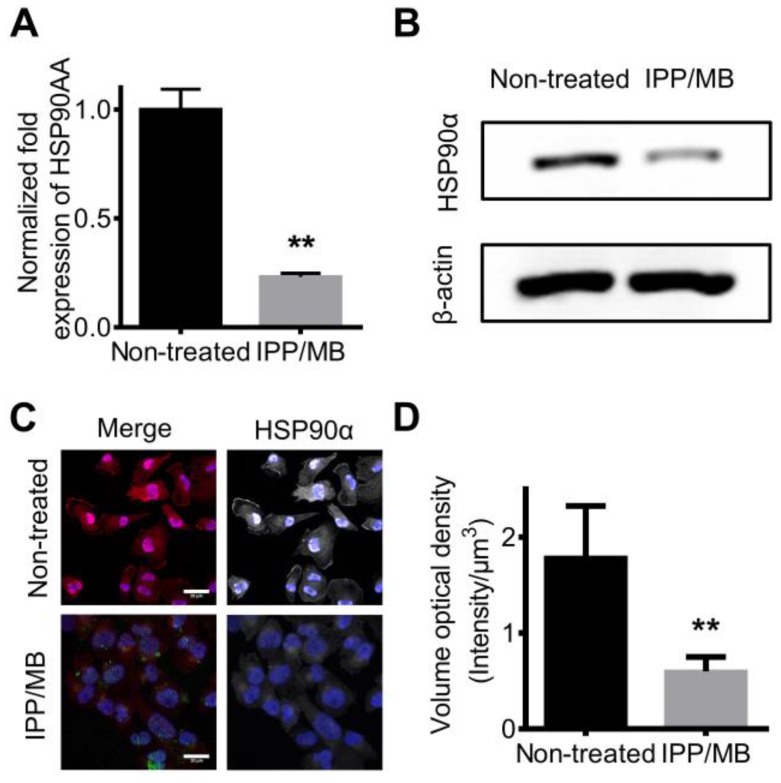
** Down-regulation of HSP90 mRNA and protein levels *in vitro*. (A)** *q*RT-PCR confirmed the expression level of HSP90AA mRNA was down-regulated by IPP/MB treatment. The data are presented as the mean ± SEM. **P<0.01. **(B-D)** Western blotting and immunofluorescence staining assay confirmed that the protein levels of HSP90α decreased significantly after incubation with the IPP/MB nanobeacon. Scale bar = 20 μm. The data are presented as the mean ± SEM. **P<0.01.

Next, the ability of IPP/MB nanobeacon to regulate mRNA and protein levels of HSP90α was further investigated. MDA-MB-231 cells were incubated with the IPP/MB nanobeacon (10 μg Fe mL^-1^, 54 nM of HSP90-MB) for 48 h. Then, total mRNA was collected and *q*RT-PCR analysis for HSP90α mRNA was performed as mentioned above. The results showed that HSP90α mRNA levels were depleted by 76.9% compared to non-treated cells (Figure 8A). This consisted with previous studies showing, in the case of molecular antisense that mRNA binding leads to enzyme activity, which degrades DNA/RNA hybrids 49. Western blotting and immunofluorescence staining confirmed that protein levels of HSP90α decreased significantly after incubation with the IPP/MB nanobeacon (Figure 8B-D), which could be explained as either translation inhibition or mRNA depletion 50. Since the IPP/MB nanobeacon can efficiently downregulate HSP90α mRNA and protein levels, it might be applied to enhance the sensitivity of chemotherapy and hyperthermia, a benefit for cancer treatment in the future.

### In vivo *T_2_*-weighted MR imaging in a tumor model

**Figure 9:**
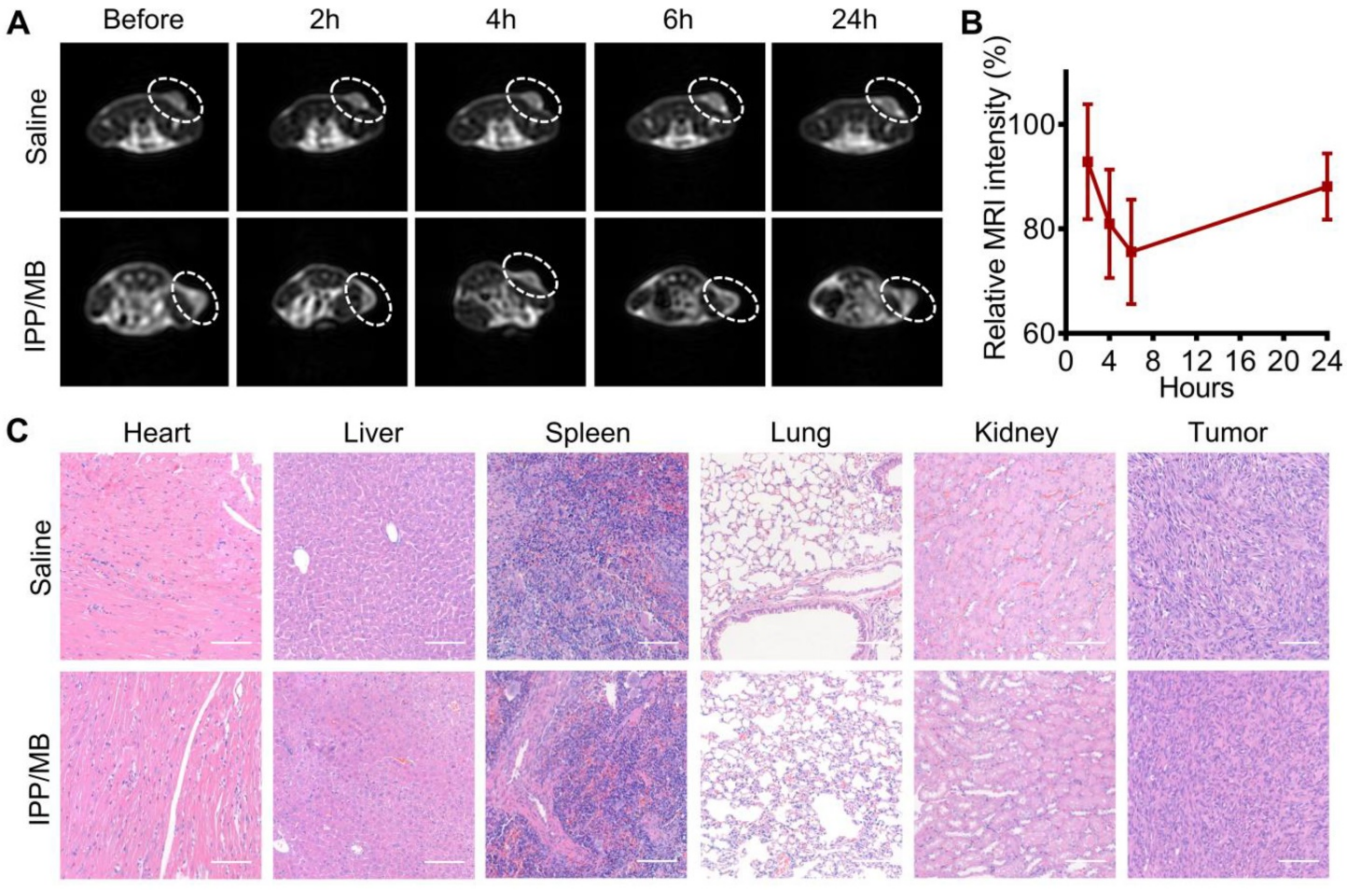
***In vivo T*_2_-weighted MR imaging and biocompatibility of the IPP/MB nanobeacon. (A)** Images of the *T*_2_-weighted MR imaging of a tumor model before and at different time points after injection of either IPP/MB or saline. **(B)** Quantitative analysis of the MRI signal intensity of the tumor sites at different time points after IPP/MB administration, relative to the corresponding time points after saline administration. The data are presented as the mean ± SEM. **(C)** H&E staining of major organs (heart, liver, spleen, lung and kidney) and tumors from mice treated with saline or the IPP/MB nanobeacon. Scale bar = 100 μm.

The potential of the IPP/MB nanobeacon to act as a *T*_2_-weighted MR imaging contrast agent was explored on a xenografted MDA-MB-231 tumor model. *T*_2_- weighted MR images of the tumor-bearing mice were obtained before and at different time points after injection of either IPP/MB or saline (Figure 9A). These images showed a decline of MR signal intensity (became darker on images) at the tumor site within the first 6 h after IPP/MB injection, after which the signal intensity slowly recovered up to 24 h post-injection. Quantitative analysis of the *T*_2_- weighted MR images revealed the IPP/MB injection gradually decreased the signal intensity from 92.8% (2 h) to 75.6% (6 h), and eventually increased to 88.1% (24 h) (Figure 9B). These results suggested that the IPP/MB nanobeacon could accumulate at the tumor site via the EPR effect *in vivo*, thus allowing for *T*_2_-weighted MR imaging of tumors 51.

### *In vivo* biocompatibility of IPP/MB nanobeacon

The biocompatibility of the IPP/MB nanobeacon was also evaluated *in vivo*. A complete blood count analysis, which included red blood cells (RBC), white blood cells (WBC), lymphocytes (Lymph), platelets (PLT), mean platelet volume (MPV), hematocrit (HCT), hemoglobin (HGB), mean corpuscular volume (MCV), as well as the mean corpuscular hemoglobin concentration (MCHC), showed no significant difference in the levels of these indicators between saline and IPP/MB treatment (Figure S9). Moreover, major organs (heart, liver, spleen, lungs, and kidneys) and tumors from mice treated with saline or the IPP/MB nanobeacon were collected, and H&E staining was performed (Figure 9C). Neither pathological tissue damage nor inflammation was observed in either group, suggesting that the IPP/MB nanobeacon had no acute systemic toxicity for mice at a dose of 3 mg Fe kg^-1^.

## Conclusions

In this study, we designed and synthesized a new kind of superparamagnetic nanobeacon for HSP90α mRNA detection and regulation in living cells, as well as enhancement of *T*_2_-weighted MR Imaging in a tumor model. The IPP/MB nanobeacon showed negligible hemolytic activity and cytotoxicity *in vitro* and had no acute systemic toxicity *in vivo*, exhibiting good biocompatibility and hemocompatibility. Confocal microscopy and flow cytometry analysis confirmed that the IPP/MB nanobeacon was able to distinguish HSP90α mRNA levels in living cells and was thus suitable for visualizing the difference between cancer and normal cells. Importantly, the IPP/MB nanobeacon was also capable of efficiently down-regulating HSP90α mRNA and protein levels, which might be beneficial for the sensitization of chemotherapy and hyperthermia of cancer. In addition, *T*_2_-weighted MR imaging ability was increased after PAMAM conjugation and Pluronic P123 modification, which allowed the IPP/MB nanobeacon to enhance *T*_2_-weighted MR image contrasts of tumors *in vivo*. Altogether, the IPP/MB nanobeacon showed promise for future applicability for tumor-related mRNA detection, regulation and multiscale imaging in the field of cancer diagnostics and therapeutics.

## Abbreviations

AF488: Alexa Fluor 488
ATCC: American type culture collection
BSA: bovine serum albumin
CDI: N,N'-carbonyldiimidazole
DAPI: 4',6-diamidino-2- phenylindole
DLS: dynamic light scattering
DMEM/ F12: Dulbecco's modified Eagle medium
DMSA: meso-2,3-dimercaptosuccinic acid
DMSO: dimethylsulfoxide
DTG: differential thermogravimetry
EDC: N-(3-dimethylaminopropyl)-N'-ethylcarbodiimide hydrochloride
EPR: enhanced permeability and retention
Em: emission wavelength
Ex: excitation wavelength
FC: field-cooled
FOV: view or field
FRET: Förster resonance energy transfer
FSE: fast spin echo
FT-IR: Fourier-transform infrared spectroscopy
G4 PAMAM: generation 4 poly (amidoamine) dendrimer
GAPDH: glyceraldehyde 3-phosphate dehydrogenase
HCT: hematocrit
HGB: hemoglobin
HSP: heat shock protein
HSP90-MB: HSP90α mRNA- specific molecular beacon
ICP-AES: Inductively coupled plasma atomic emission spectroscopy
IONC: cubic-shaped iron oxide nanoparticle
IONP: iron oxide nanoparticle
IPP: IONC-PAMAM-P123
Lymph: lymphocytes
LSCM: laser scanning confocal microscopy
MB: molecular beacon
MCHC: mean corpuscular hemoglobin concentration
MCV: mean corpuscular volume
MPV: mean platelet volume
MR: magnetic resonance
MRI: magnetic resonance imaging
MWCO: molecular weight cut-off
NCG: NOD CRISPR Prkdc Il2r gamma
OA: oleic acid
P123: triblock copolymer Pluronic P123
PBS: phosphate buffered saline
PDI: polydispersity index
PEI: polyethylenimine
PLGA: poly(lactic-co-glycolic acid)
PLT: platelets
PVDF: polyvinylidene fluoride or polyvinylidene difluoride
qRT-PCR: quantitative real-time polymerase chain reaction
RBC: red blood cells
SDS-PAGE: sodium dodecyl sulfate polyacrylamide gel electrophoresis
SE-IR: spin echo-inversion recovery
SEM: standard error of the mean
SPF: specific-pathogen-free
SQUID: superconducting quantum interference device
sulfo-NHS: N-hydroxysulfosuccinimide sodium salt
TBST: a mixture of tris-buffered saline and Tween 20
TEM: transmission electron microscope
TGA: thermogravimetric analysis
TMB: 3,3',5,5'-tetramethylbenzidine
TNBC: triple negative breast cancer
TRAIL: TNF-related apoptosis-inducing ligand
WBC: white blood cells
XRD: X-ray diffraction
ZFC: zero-field-cooled
