# Supplementary material for: Dendrimer-Functionalized Superparamagnetic Nanobeacons for Real-Time Detection and Depletion of HSP90α mRNA and MR Imaging
Source: Theranostics. 2019 Aug 12;9(20):5784–96. doi: 10.7150/thno.36545 (PMC6735378; doi:10.7150/thno.36545)
Supplement: Supplementary file 1 — Supplementary figures and tables. [file thnov09p5784s1.pdf]

# Supporting Information

## Dendrimer-functionalized superparamagnetic nanobeacons for real-time detection and depletion of HSP90α mRNA and MR imaging

Zhongyuan Chen[1], Yueting Peng[1], Xiaoxue Xie[1], Yi Feng[1], Tingting Li[1,3], Shun Li[1,3], Xiang Qin[1,3], Hong Yang[1,3], Chunhui Wu[1,3], Chuan Zheng [2], Jie Zhu [2], Fengming You [2], Yiyao Liu[1,2,3 *]

[1]Department of Biophysics, School of Life Science and Technology, University of Electronic Science and Technology of China, Chengdu 610054, Sichuan, P.R. China;

[2]Hospital of Chengdu University of Traditional Chinese Medicine, No. 39 Shi-er-qiao Road, Chengdu 610072, Sichuan, P.R. China;

[3]Center for Information in Biology, University of Electronic Science and Technology of China, Chengdu 610054, Sichuan, P.R. China

**Synthesis and characterization of oleic acid-coated iron oxide nanocubes (IONC-OA)**

The preparation of the iron oleate precursor and the synthesis and purification of iron oxide nanocubes followed previous descriptions [1] with minor modifications. In brief, iron(III) chloride hexahydrate (40 mmol, 10.8 g) was first dissolved in 20 mL Milli-Q water to prepare 2 M stock solution. Sodium oleate (82%, NLT 120 mmol, 45 g) was dissolved in a mixture of 40 mL Milli-Q water, 60 mL ethanol and 140 mL n-hexane in a round-bottom flask, followed by 20 mL iron(III) chloride 2 M stock solution added dropwise with magnetic stirring. The solution was stirred until the reagents were completely dissolved, after which the solution was refluxed for 4 h. The solution was cooled and allowed to phase separate; the brown hexane phase was separated from the clear $H_2O$/ethanol phase and washed three times with Milli-Q water/ethanol mixture (50 mL/10 mL). The hexane phase was then transferred into a round-bottom flask, and the solvent was removed by distillation at 70 °C and dried in an incubator shaker at 50 °C for three days, yielding a red-brown viscous product. The iron oleate precursor was dissolved into 1-octadecene to make a 40% (m:m) stock solution.

Iron oxide nanocubes were synthesized by thermal decomposition of the dissolved precursor in a high-boiling solvent in the presence of a mixture of oleic acid and sodium oleate. In brief, the synthesis was performed in a 100-mL three-necked round-bottom flask using 25 mL of 1-octadecene (b.p. = 315 °C),

5 mmol iron oleate, 0.715 mmol oleic acid, and 0.715 mmol sodium oleate. The heating rate and holding times were adjusted using a home-made Arduino-based PID controller (Figure S2). The temperature of the synthesis solution was first increased to 120 °C under a constant flow of nitrogen gas at a heating rate of 5 °C $min^{-1}$, with magnetic stirring, and held at this temperature for 1 h to remove trace amounts of water. Then, the magnetic stir bar was removed, and the temperature of the synthesis solution was increased to the reflux temperature at a heating rate of 3 °C $min^{-1}$, and allowed to reflux for 40 min. The flow of nitrogen gas was momentarily increased to drive vapors from the volatile decomposition products to the gas absorber (filled with 95% ethanol), if the temperature is fluctuated while refluxing. Finally, the solution was allowed to cool to room temperature under a constant flow of nitrogen gas.

After cooling to room temperature, 75 mL of ethanol was added to the solution followed by a 5-min sonication. Then, the oleic acid-coated iron oxide nanocubes were precipitated by centrifugation at 5000 *g* for 5 min. The nanocubes were washed using n-hexane and ethanol. After 3-4 washing cycles, the nanocubes redispersed in n-hexane. The n-hexane dispersions, with a concentration of 10 mg iron oxide $mL^{-1}$, were used for further conjugation. The sizes and shapes of the nanoparticles were determined using transmission electron microscopy (TEM). Dilute n-hexane dispersions of the iron oxide nanocubes were distributed over carbon-coated TEM grids and analyzed using a JEM-2010 electron microscope. Size distributions of the nanocubes were

determined by measuring the edge length of ~3000 nanocubes using ImageJ software.

**Phase transfer by ligand-exchange with meso-2,3-dimercaptosuccinic acid (DMSA)**

Ligand-exchange was used to replace oleic moieties by meso-2,3-dimercaptosuccinic acid (DMSA) following previous descriptions [2] with minor modifications. IONC-OA (100 mg) was precipitated from the n-hexane suspension by adding ethanol and using a strong magnetic field gradient, discarding the supernatant. After several ethanol washes, the IONC-OA was redispersed in toluene (20 mL), added to a solution of DMSA (180 mg) in DMSO (5 mL) and mixed with sonication. The mixture was incubated at room temperature for 48 h in an incubator shaker at 250 rpm. After the reaction, the translucent solvent containing the oleic acid was discarded and the black particles (IONC-DMSA) attached to the walls of the glass bottle were redispersed in ethanol with sonication and vortexing. This mixture was magnetically separated and redispersed in ethanol several times to clean the particles. Finally, the IONC–DMSA was redispersed in Milli-Q water, basified to pH 9.0 with sodium hydroxide (0.01 M NaOH). Filtration through a 0.22-μm pore membrane and storage in 4 °C was carried out as a final step.

**Synthesis of PAMAM-conjugated iron oxide nanocubes (IONC-PAMAM)**

After ligand-exchange, multilayer DMSA moieties were coated on the surface of the IONC core, and the remaining carboxylates of DMSA were used for further conjugation [3]. IONC-PAMAM conjugates were prepared by crosslinking generation 4 PAMAM dendrimers (G4 PAMAM) to carboxylic-terminated IONC-DMSA via EDC and sulfo-NHS chemistry according to previous descriptions (Figueroa et al. 2014) with minor modifications. In brief, 100 mg of IONC-DMSA dispersed in water was acidified to pH 1.0-2.0 with diluted hydrochloric acid (0.01 M HCl). The acidified IONC-DMSA was precipitated, magnetically separated, and washed with ethanol twice to remove the excess hydrochloric acid. The supernatant ethanol was removed with pipettes, and 9 mL of DMSO were added to the IONC-DMSA precipitate and redispersed with sonication. 10 mg of EDC (0.052 mM) and 10 mg of sulfo-NHS (0.046 mM) were dissolved in 1 mL of DMSO. The EDC/sulfo-NHS DMSO solution was added to 9 mL of IONC-DMSA DMSO dispersion dropwise with magnetic stirring. The stirring continued for 15 min, allowing EDC and sulfo-NHS to react with carboxyl groups on the surface of IONC-DMSA and form amine-reactive sulfo-NHS esters. Then, the amine-reactive IONC-DMSA was added to 10 mg of G4 PAMAM in 10 mL of PBS dropwise with magnetic stirring. Finally, IONC-PAMAM was separated from the reaction mixture using a magnet. The supernatant was discarded, and IONC-PAMAM was acidified with dilute hydrochloric acid (0.01 M HCl), sonicated to form a clear solution and washed six times with 0.01 M HCl using a centrifuge filter (30,000 MWCO). IONC-

PAMAM was redispersed in 0.01 M HCl, and 0.01 M NaOH were added to reach pH 7.0. The neutralized dispersion of IONC-PAMAM was stored at 4 °C until used.

**Synthesis of IONC-PAMAM-P123**

Pluronic P123 (MW 5,800) was conjugated to the primary amine of PAMAM according to previous descriptions [4]. In brief, after dissolving 0.05 mM P123 in 2.5 mL of anhydrous acetonitrile, 0.05 mM CDI was dissolved in 2.5 mL of anhydrous acetonitrile and added dropwise to the P123 solution in the presence of nitrogen gas. The mixture was stirred for 4 h at 40 °C, diluted with 7.5 mL of water and dialyzed six times against 20% ethanol. For IONC-PAMAM-P123 conjugation, mono-imidazolylcarbonyl-activated P123 (10 mg $mL^{-1}$, 580 μL) was added dropwise with constant stirring to 10 mL of 0.5 mg Fe $mL^{-1}$ IONC-PAMAM, with a final P123 concentration of ~0.1 mM. The reaction mixture was stirred at 25 °C for 48 h. The products were washed six times with Milli-Q water using a centrifuge filter (30,000 MWCO) to remove unbound P123. IONC-PAMAM-P123 was redispersed in Milli-Q water and stored at 4 °C until used.

**Calculation of the HSP90-MB content in IPP/MB nanobeacons.**

Based on TEM images, synthesized cube shape of IONC-OA had an average edge length (L) of 10.7 nm. So the average volume of each IONC core was calculated as

$$V = L^3 \approx 1225\ nm^3 = 1.225\times10^{-24}\ m^3.$$

Since the crystalline structure of the IONC was similar to synthetic magnetite ($Fe_3O_4$), we assume the density of the IONC (ρ) was the same as bulk $Fe_3O_4$, which was about 5180 kg/m$^3$, the mass of each IONC core (M) was calculated as

$$M = \rho V = 6.3455\times10^{-21}\ kg = 6.3455\times10^{-12}\ \mu g.$$

The mass of Fe in each IONC core ($M_{Fe}$) was calculated as

$$M_{Fe} = M\times((56\times3) / (56\times3+16\times4)) \approx 5.0764\times10^{-12}\ \mu g.$$

The molecular weights of HSP90-MB was 11577.97 μg/μmole according to manufacturer's synthesis report.

So the mass of each HSP90-MB ($M_{MB}$) was calculated as

$$M_{MB} = MW(HSP90\text{-}MB) / 10^{-6} N_A = 11577.97 / (10^{-6}\times6.02\times10^{23})$$

$$=1.92325\times10^{-14}\ \mu g.$$

Since the mass ratio of Fe in IPP to HSP90-MB was determined to be 16:1 by agarose gel electrophoresis, and each IPP particle only have one IONC core according to TEM images, the ratio of molecular number of HSP90-MB to particle number of IPP (R) was calculated as

$$R = (1 / M_{MB}) : (16 / M_{Fe}) = M_{Fe} / 16\ M_{MB} \approx 16.5 : 1.$$

So there are about 16 ~ 17 HSP90-MBs in each IPP/MB nanobeacons.

## References


1. Wetterskog E, Agthe M, Mayence A, Grins J, Wang D, Rana S, et al. Precise control over shape and size of iron oxide nanocrystals suitable for assembly into ordered particle arrays. Sci Technol Adv Mater. 2014; 15: 055010.
2. Palma S, Marciello M, Carvalho A, Veintemillas-Verdaguer S, Morales MDP, Roque ACA. Effects of phase transfer ligands on monodisperse iron oxide magnetic nanoparticles. J Colloid Interface Sci. 2015; 437: 147-55.
3. Chen ZP, Zhang Y, Zhang S, Xia JG, Liu JW, Xu K, et al. Preparation and characterization of water-soluble monodisperse magnetic iron oxide nanoparticles via surface double-exchange with DMSA. Colloid Surface A. 2008; 316: 210-6.
4. Gu J, Fang X, Hao J, Sha X. Reversal of P-glycoprotein-mediated multidrug resistance by CD44 antibody-targeted nanocomplexes for short hairpin RNA-encoding plasmid DNA delivery. Biomaterials. 2015; 45: 99-114.

**Table S1. Sequences of oligonucleotides.**

| Name | Sequence (5'-3') |
|---|---|
| HSP90-MB | **Alexa Fluor 488**-TTGGATCTCTGTGCCTACGTGTGCTCCGATCCAA-**BHQ1** |
| MB target | GGAGCACACGTAGGCACAGA |
| MB single-base mismatched | GGTGCACACGTAGGCACAGA |
| MB two-base mismatched | GGTGCACACGTCGGCACAGA |
| MB three-base mismatched | GGTGCACACGTCGGCTCAGA |
| MB four-base mismatched | GGTGCACACGTCGGCTCAGT |
| MB non-target | AACGACGAACTCGGGGGAAC |
| HSP90AA_F | CAGGAGATGGTTAAACACTAG |
| HSP90AA_R | TGGACACTAAGAGAACACAT |

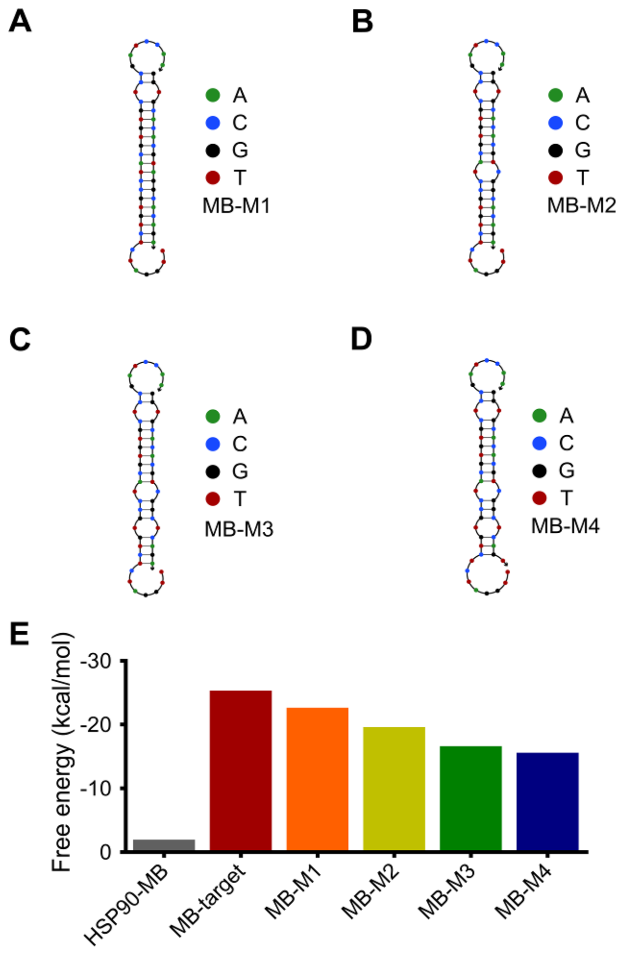


**Figure S1.** (**A-D**) A 1-4 base mismatched sequence and HSP90-MB hybridization secondary structures and (**E**) the corresponding free energy were predicted using NUPAK.

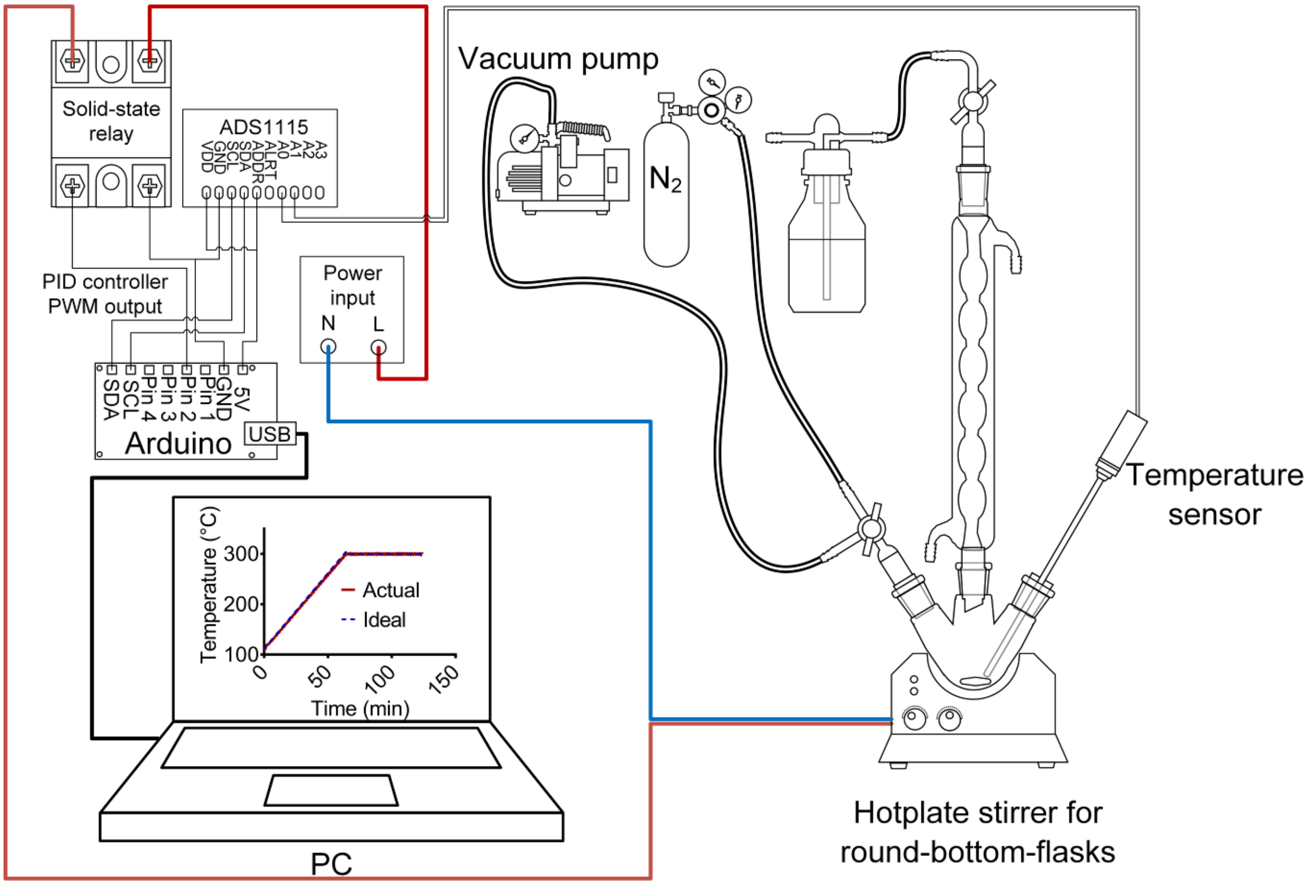


**Figure S2.** The thermal decomposition experimental setup for the synthesis of iron oxide nanocrystals. The heating rate was controlled by an Arduino-based home-made PID controller. The temperature of the reaction was recorded by a personal computer through serial port communication with Arduino. The result showed that the temperature linearly increased at a heat rate of 3 °C $min^{-1}$.

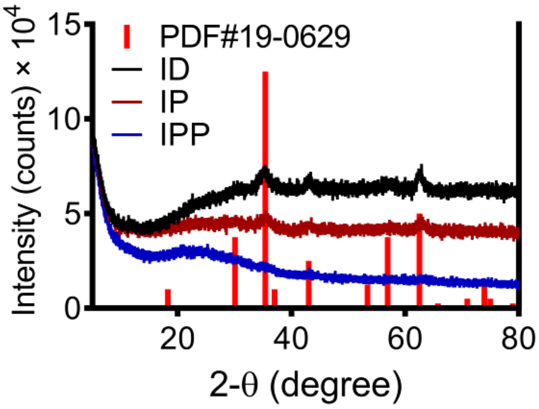


**Figure S3.** The XRD pattern of ID, IP and IPP confirmed the IONC core had the same crystalline structure as synthetic magnetite ($Fe^{2+}Fe^{3+}{}_2O_4$, PDF#19-0629).

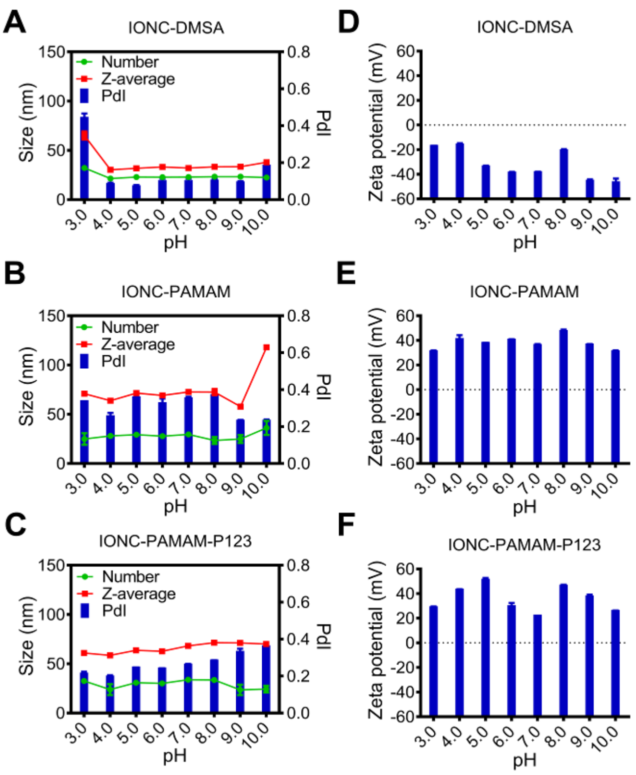


**Figure S4**. DLS analysis of the size (A-C) and zeta potential (D-F) of ID, IP, and IPP over the ranges of pH 3.0-10.0. The data are presented as the mean ± SEM.

| Line | 1 | 2 | 3 | 4 | 5 | 6 | 7 |
|---|---|---|---|---|---|---|---|

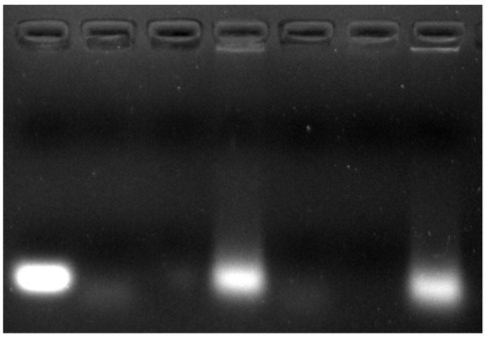

| | | | | | | | |
|---|---|---|---|---|---|---|---|
| HSP90-MB | + | + | + | + | + | + | + |
| IPP | - | - | + | + | + | + | + |
| DNase I only | - | + | - | - | - | + | - |
| Heparin only | - | - | - | + | - | - | - |
| Heparin + DNase I | - | - | - | - | + | - | - |
| DNase I + Heparin | - | - | - | - | - | - | + |

**Figure S5. Agarose gel electrophoresis assay of HSP90-MB protection.** Line 1: HSP90-MB was detected by agarose gel electrophoresis, as a positive control; Line 2: HSP90-MB was incubated with and rapidly degraded by DNase I; Line 3: HSP90-MB was incubated with IPP to form IPP/MB nanobeacon; Line 4: After forming IPP/MB nanobeacon, heparin was added to release the HSP90-MB; Line 5: After forming IPP/MB nanobeacon, heparin was added to release the HSP90-MB, and DNase I was added to degrade the HSP90-MB released; Line 6: After forming IPP/MB nanobeacon, DNase I was added, no HSP90-MB was detected; Line 7: After forming IPP/MB nanobeacon, DNase I was added; After incubated with DNase I at 37 °C for 1 h, the DNase I was inactivated by EDTA and 65 °C treatment; And heparin was added to release the HSP90-MB protected by IPP nanoparticles.

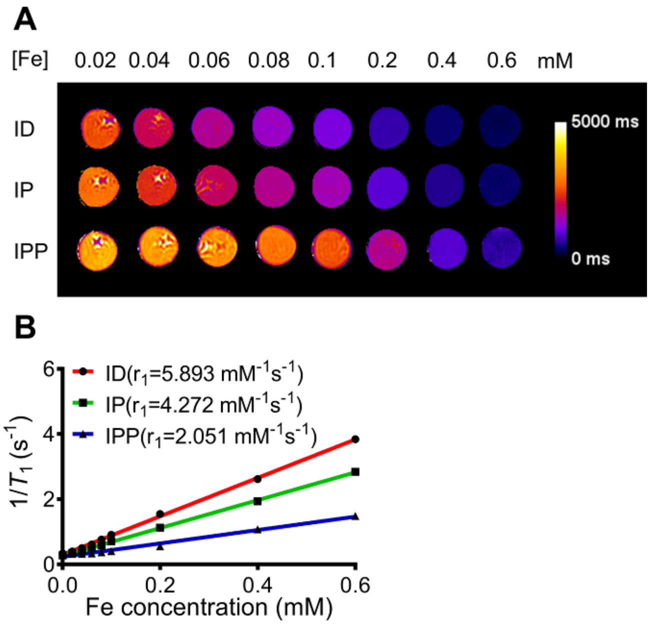


**Figure S6**. ***$T_1$*-mapping MR imaging and $r_1$ relaxivity of ID, IP and IPP.** (**A**) *$T_1$* relaxation time was measured using SE-IR sequence with increasing inversion times (TI = 50, 400, 1100 and 2500 ms), and the $T_1$-mapping images was generated. (**B**) The $r_1$ relaxivity of each sample were determined by the linear fitting of 1/*$T_1$* as a function of the Fe concentration. The data are presented as the mean ± SEM.

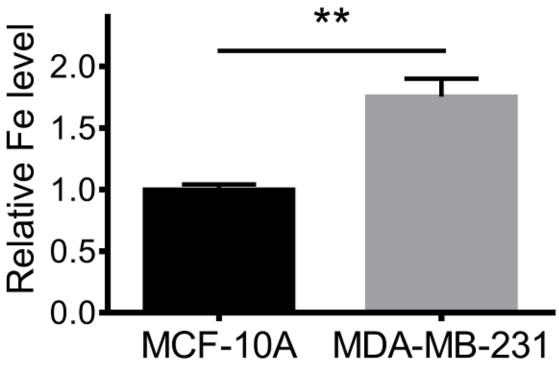


**Figure S7.** Cellular uptake behavior of the IPP/MB nanobeacon in the MDA-MB-231 or MCF-10A cell lines. The data are presented as the mean ± SEM. **$P$<0.01.

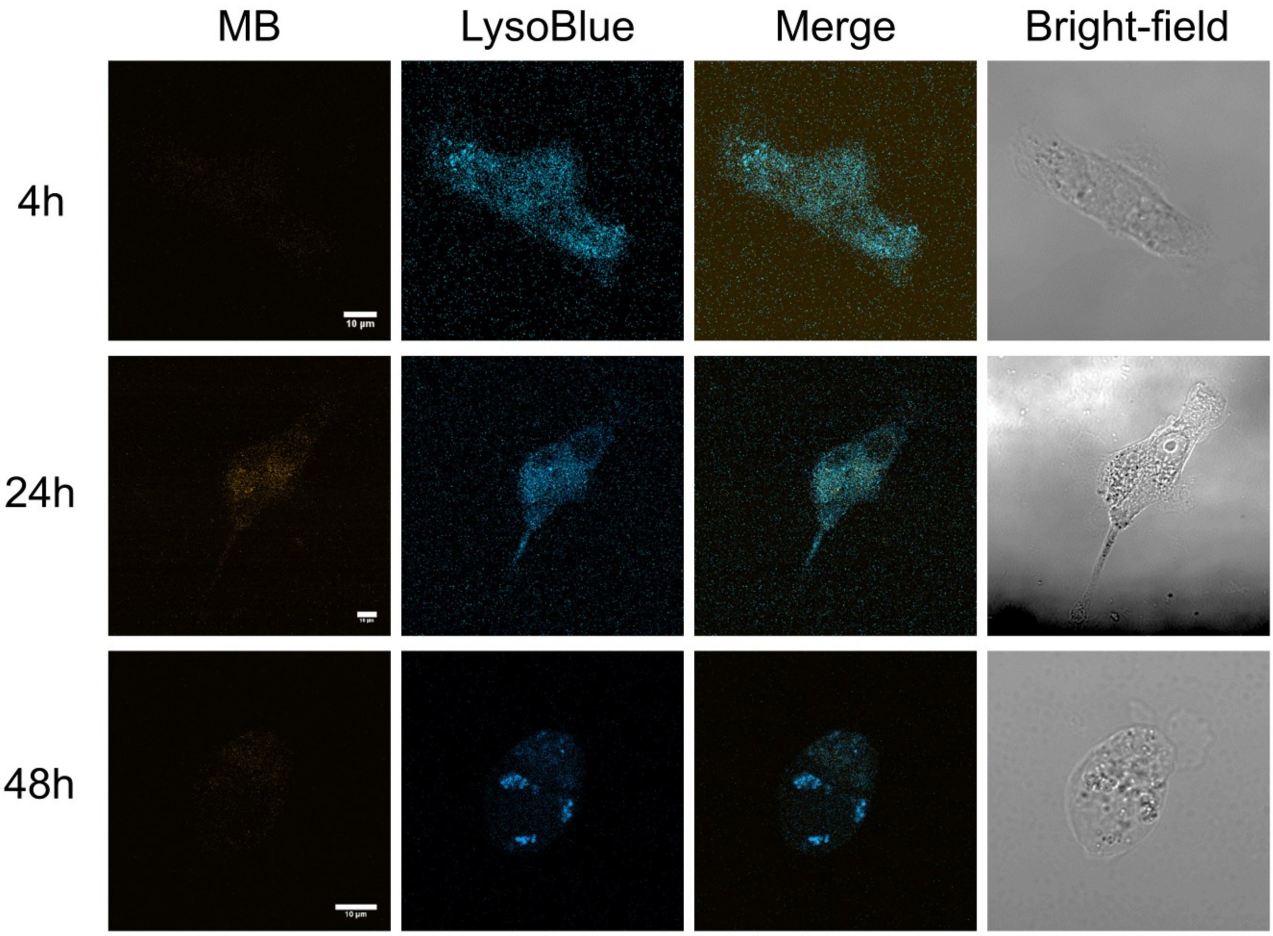


**Figure S8.** LSCM images of MCF-10A cells incubated with the nanobeacons for 4, 24 and 48 h. The fluorescence of the nanobeacon did not recover significantly during the period of the experiment, indicating the low-expression of HSP90AA mRNA in MCF-10A cells. Scale bar = 10 μm.

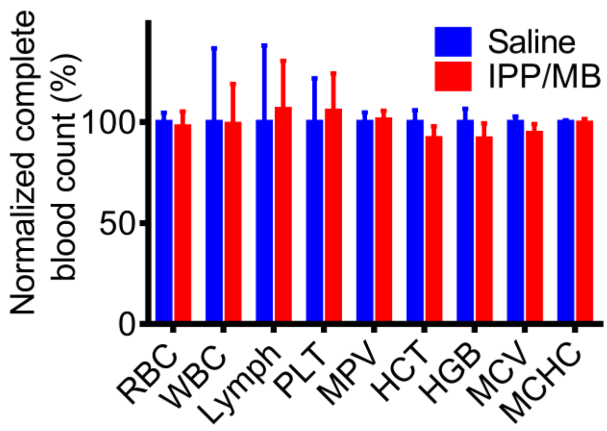


**Figure S9.** Complete blood count results (RBC, WBC, Lymph, PLT, MPV, HCT, HGB, MCV, and MCHC) of mice at 72 h after intravenous injection of the IPP/MB nanobeacon (30 µg Fe $mL^{-1}$ in 100 µL saline) or saline (100 µL).

**The distribution curves for the size and Zeta potential of ID, IP, IPP and IPP/MB:**

IONC-DMSA pH 3:

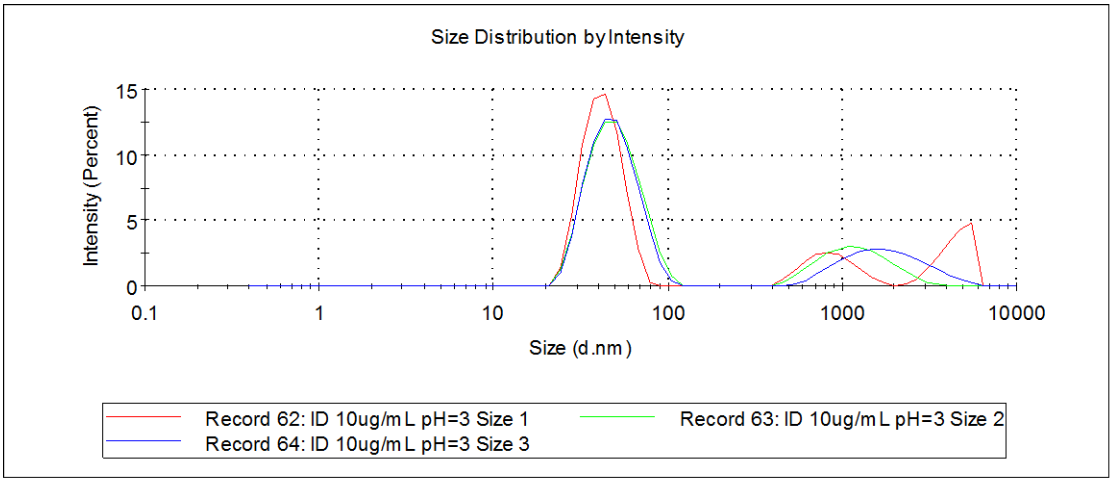


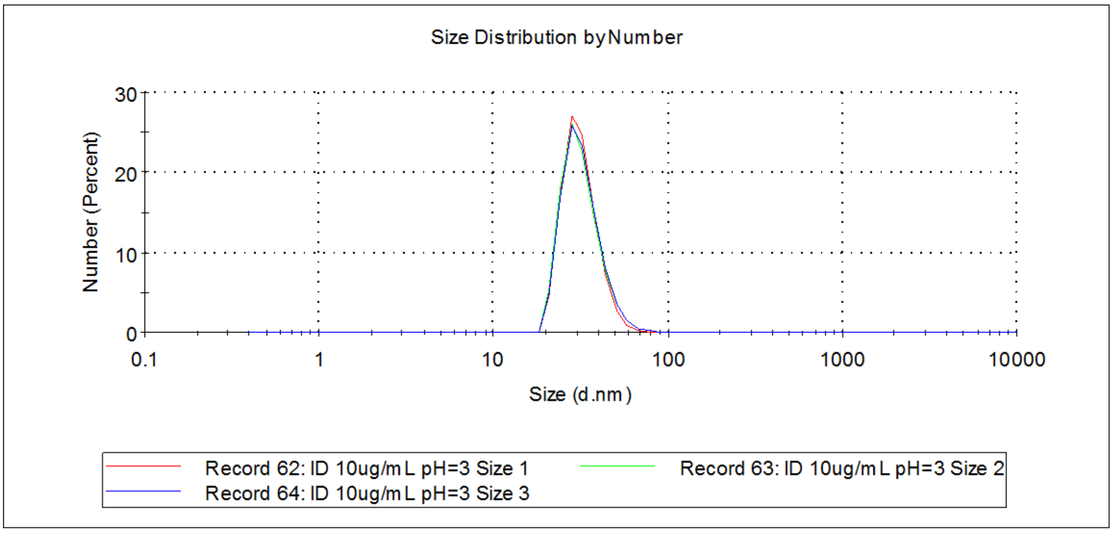


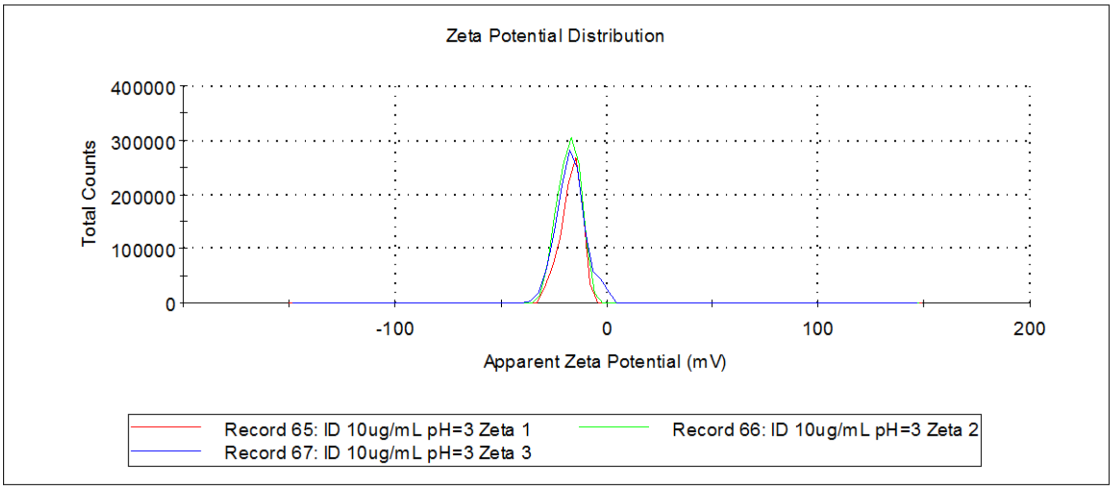

IONC-DMSA pH 4:

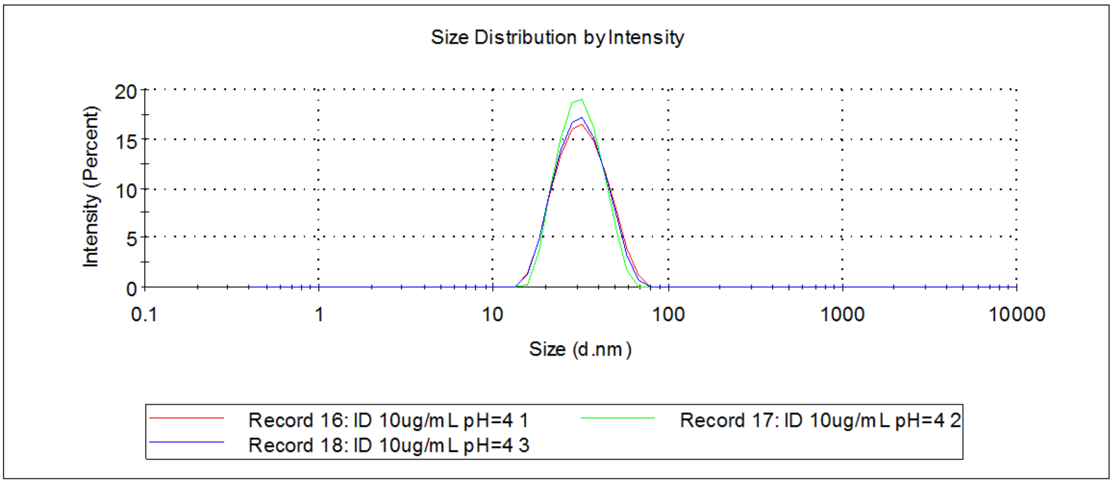


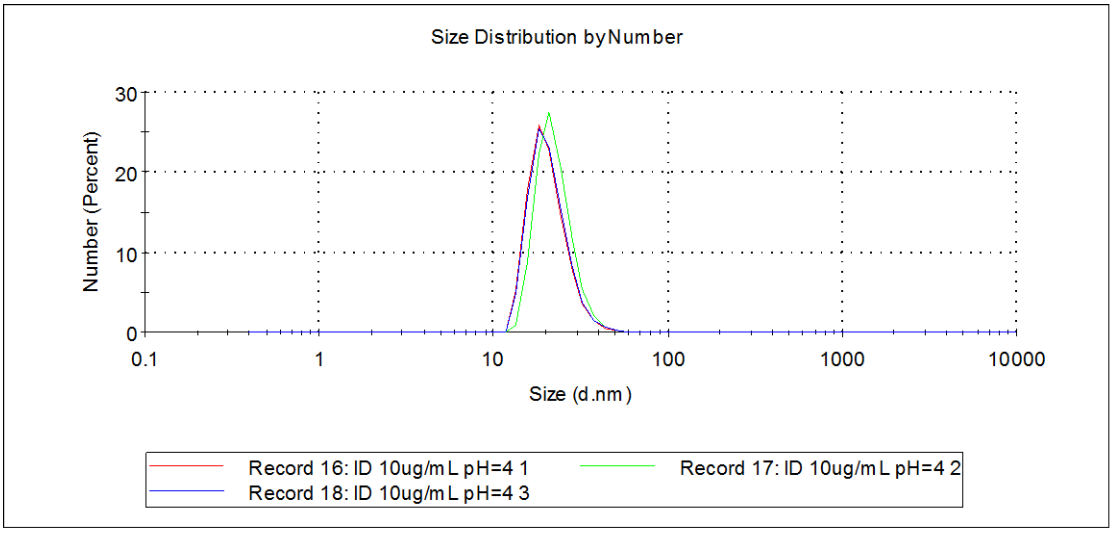


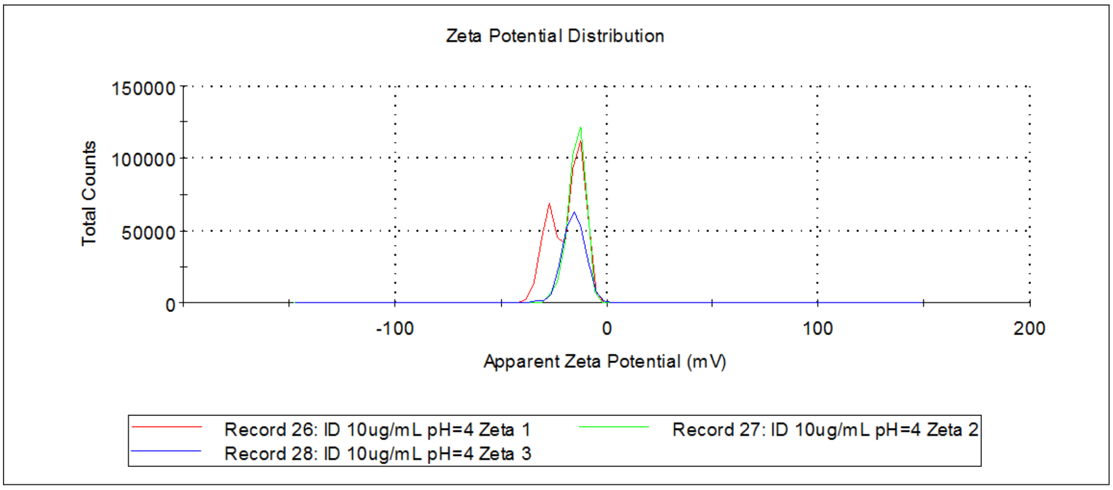

IONC-DMSA pH 5:

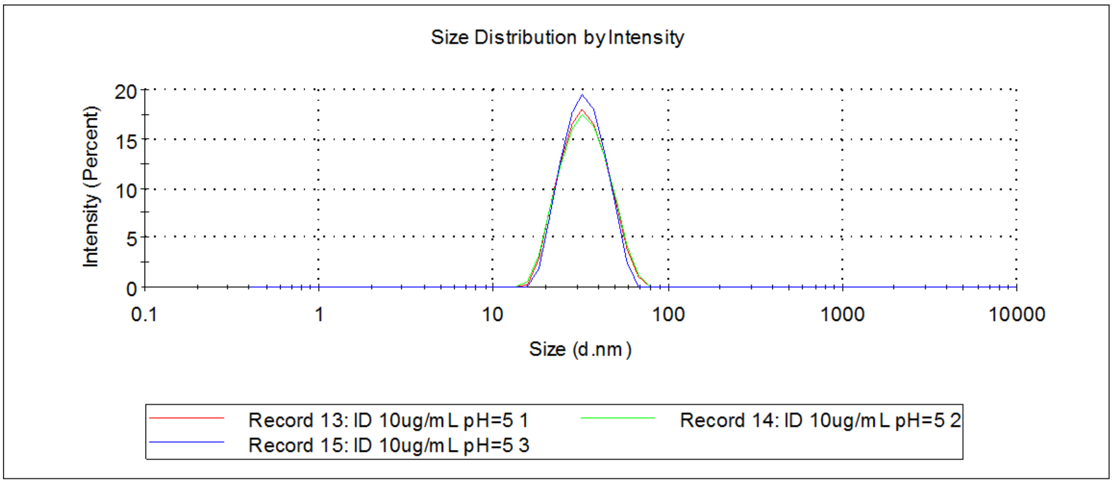


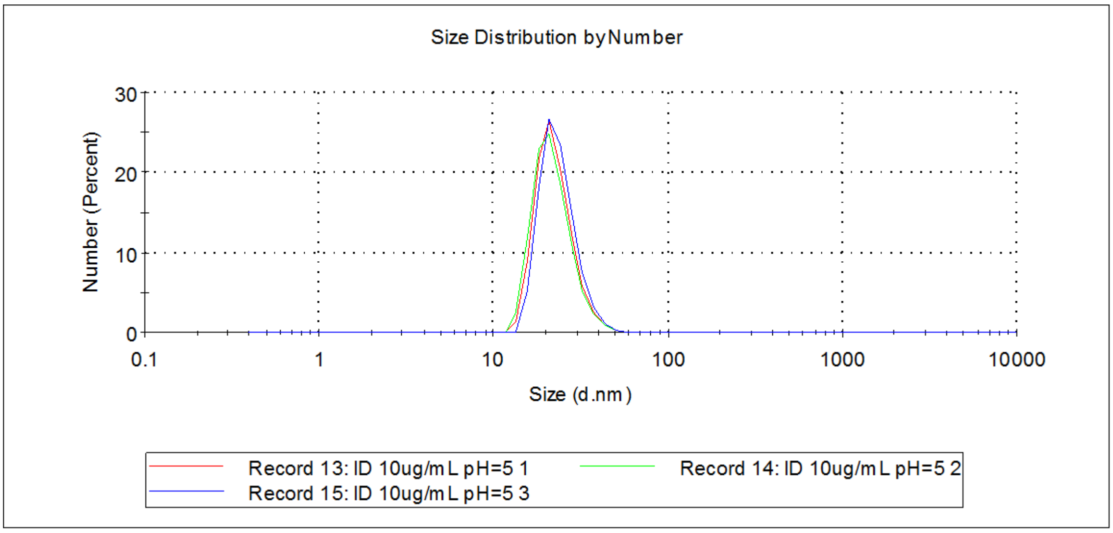


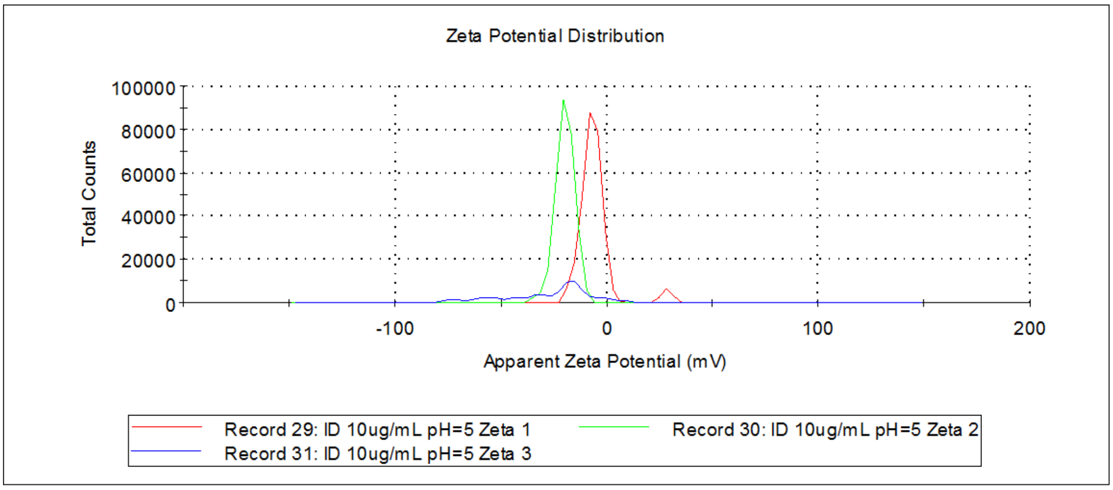

IONC-DMSA pH 6:

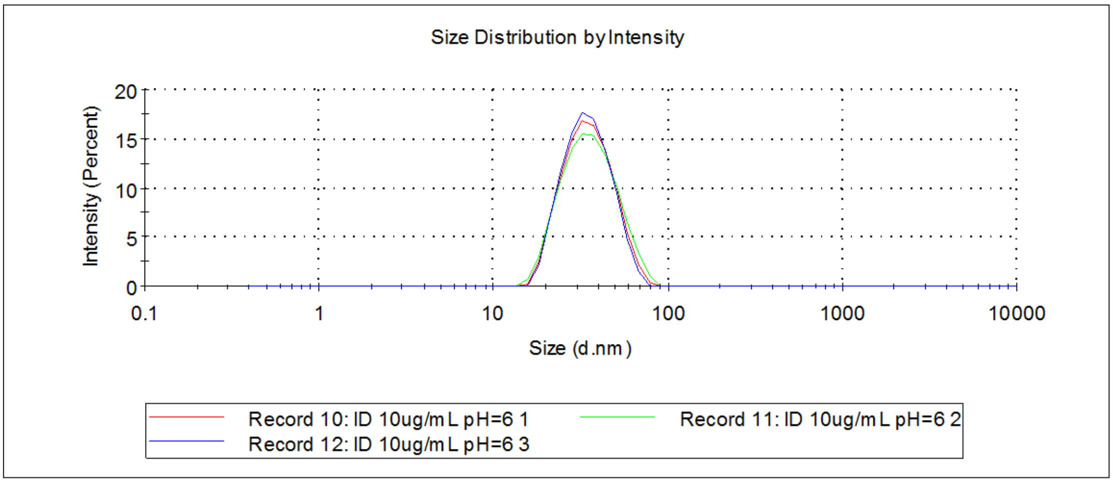


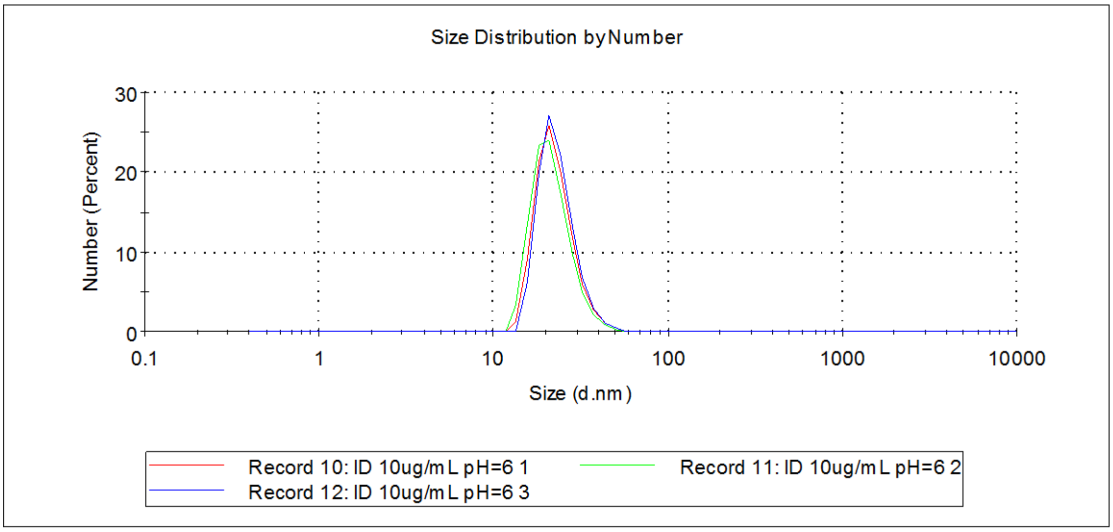


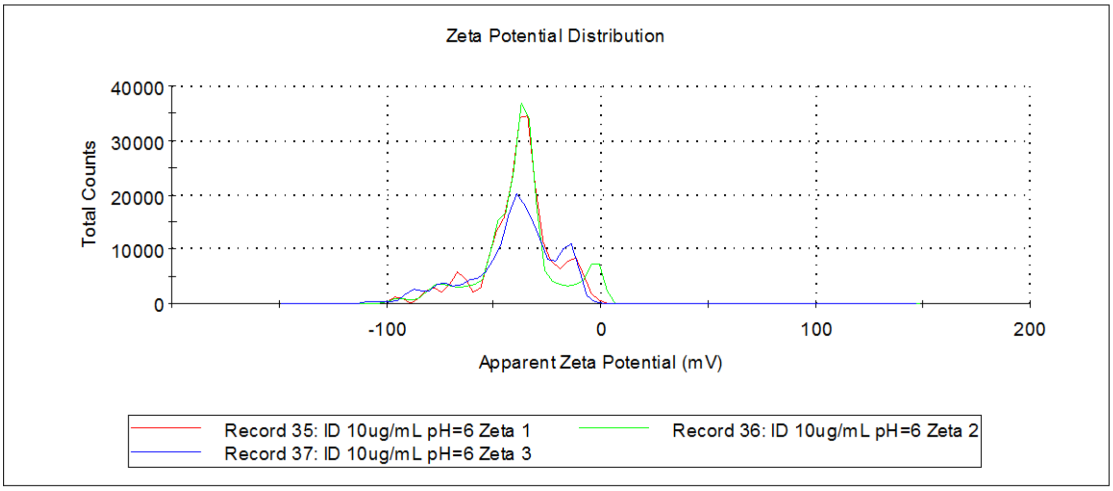

IONC-DMSA pH 7:

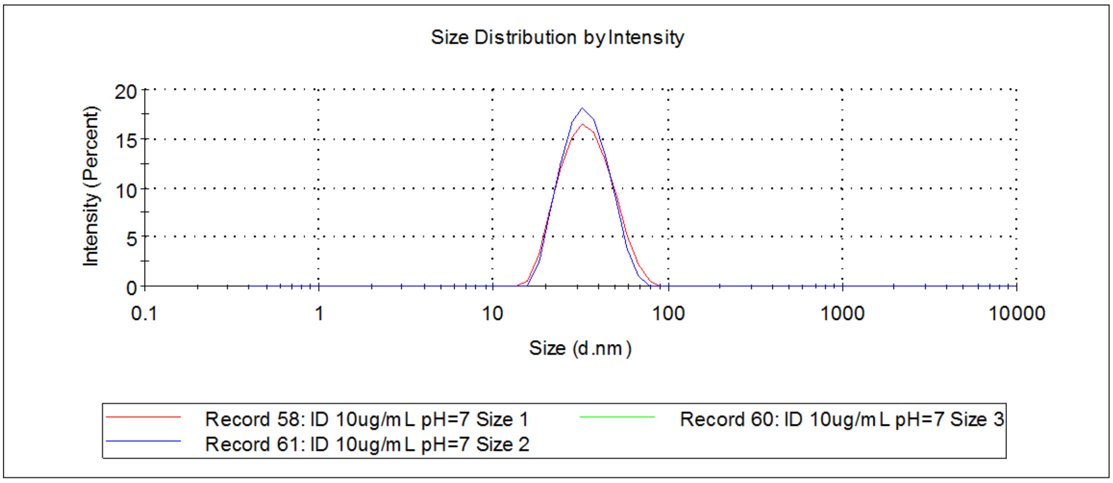


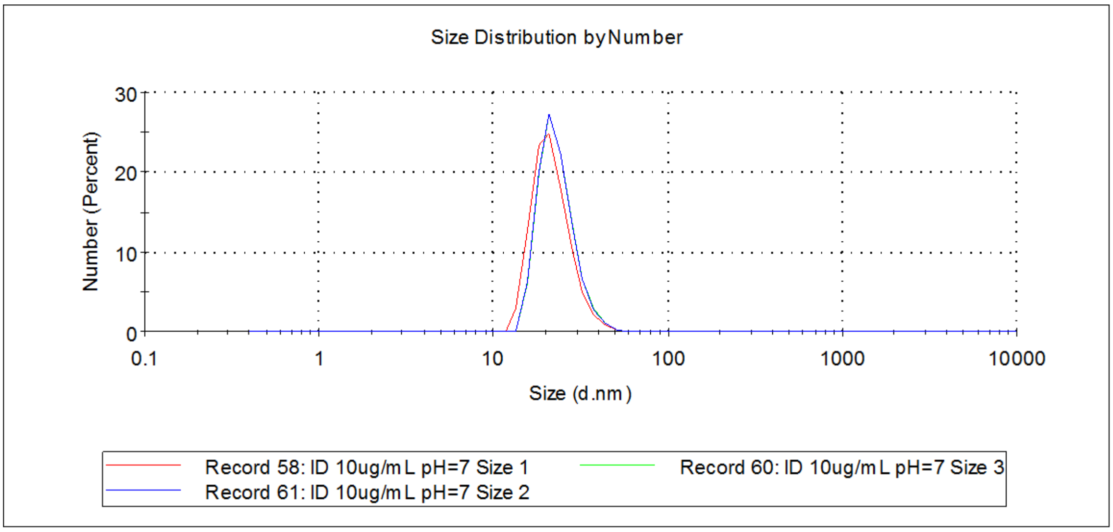


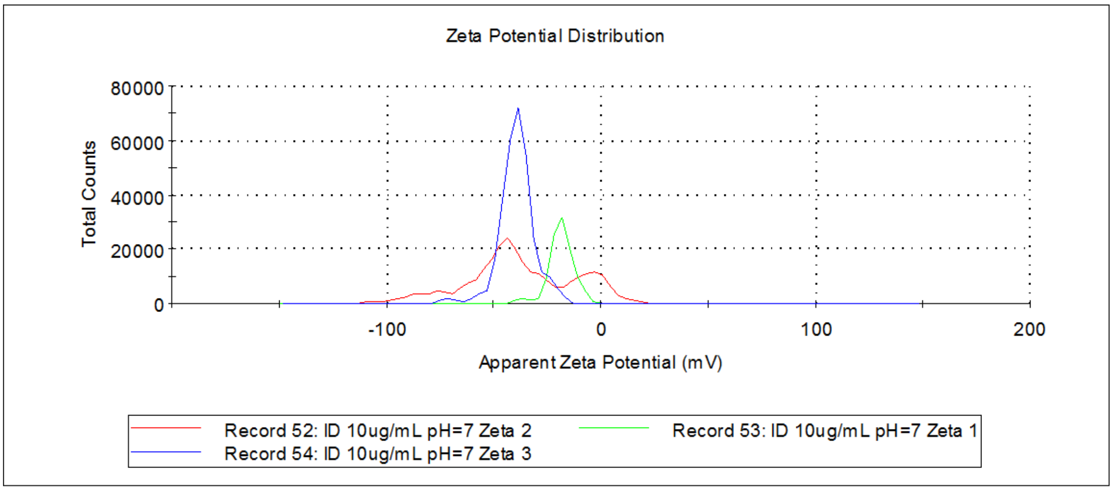

IONC-DMSA pH 8:

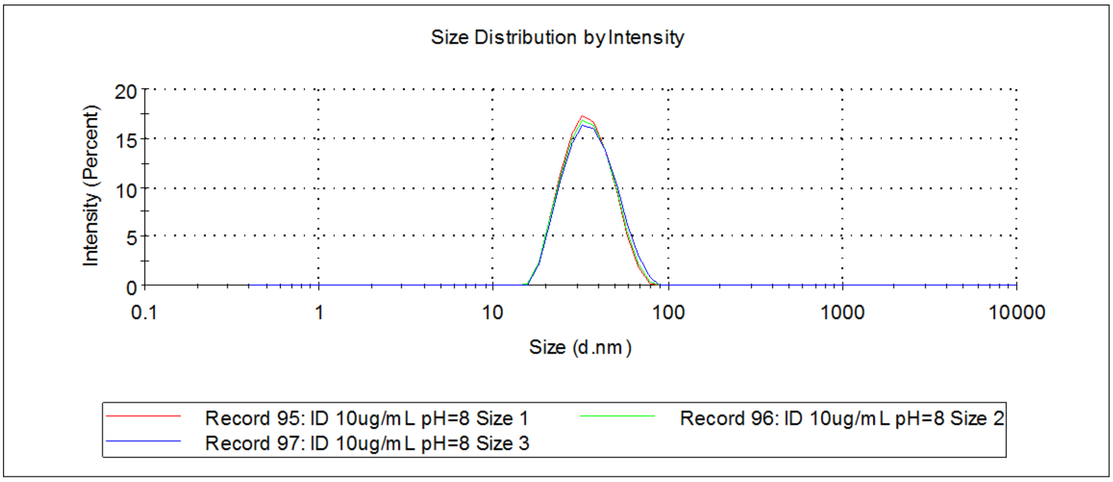


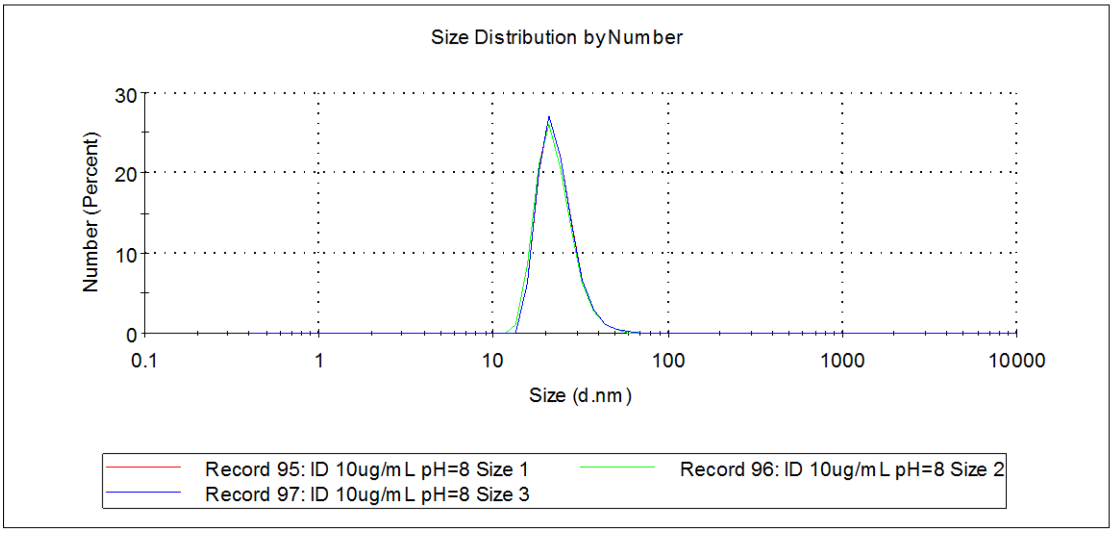


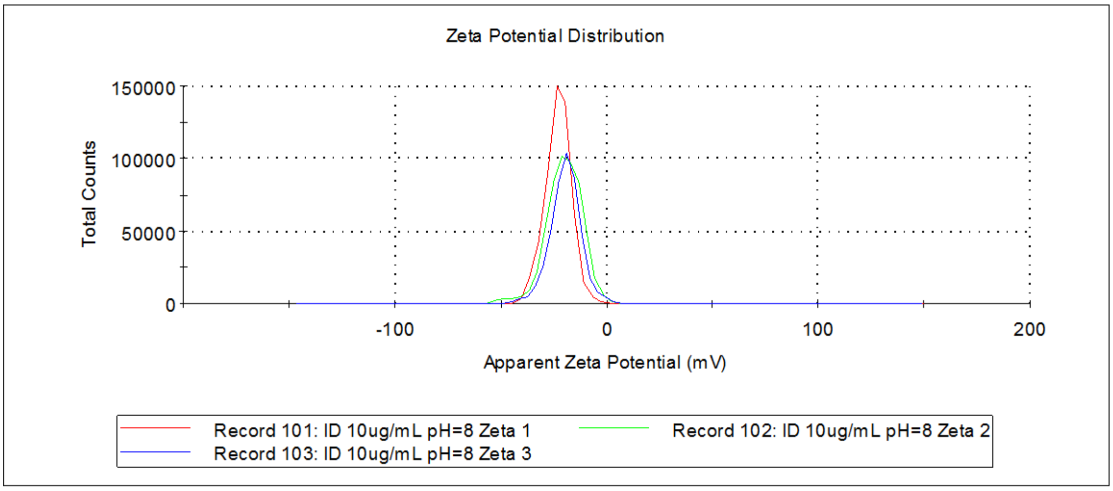

IONC-DMSA pH 9:

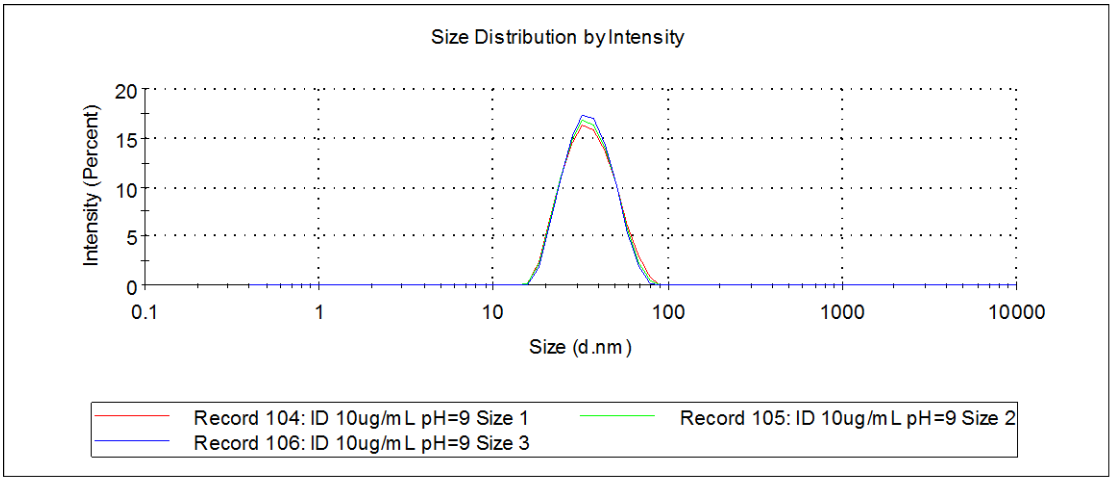


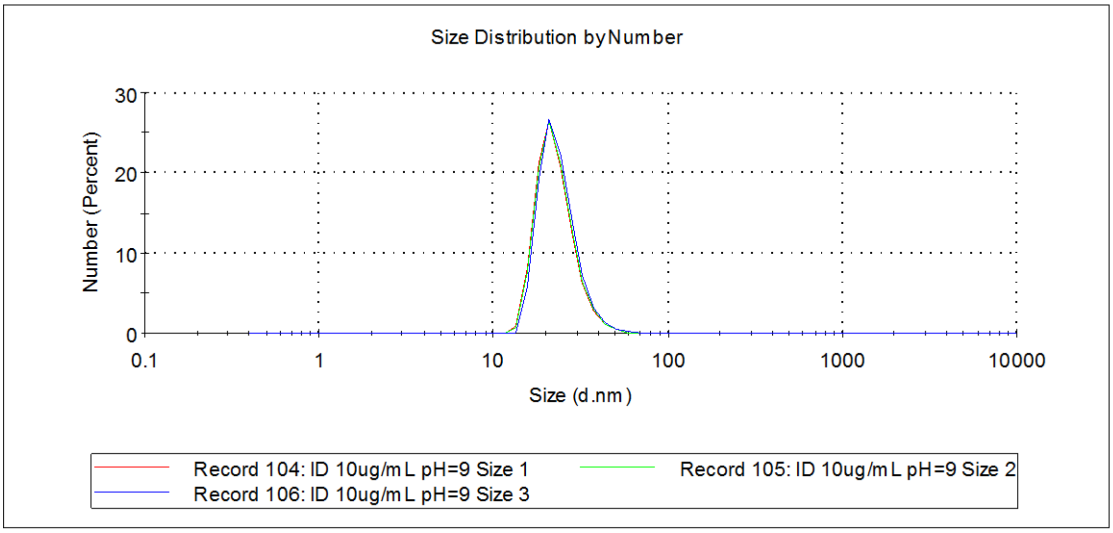


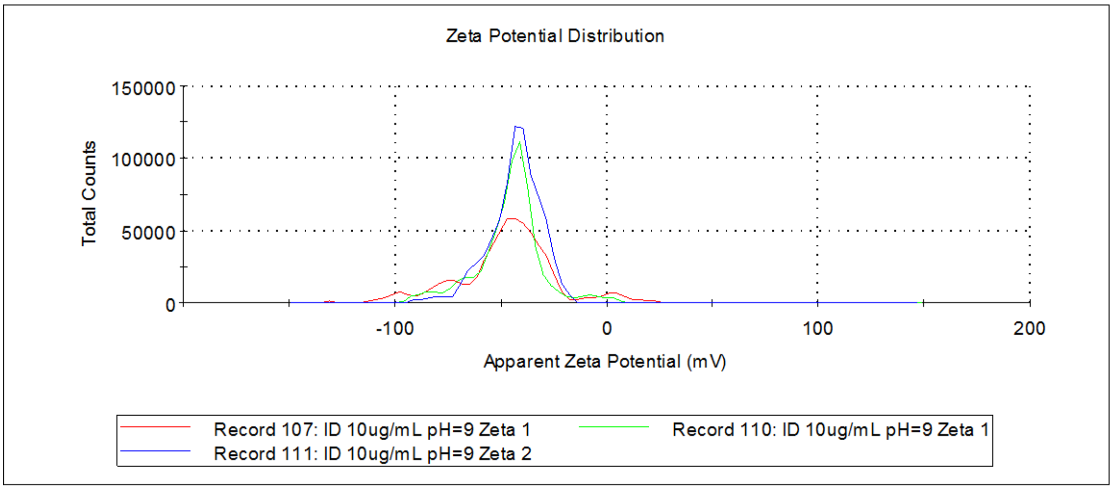

IONC-DMSA pH 10:

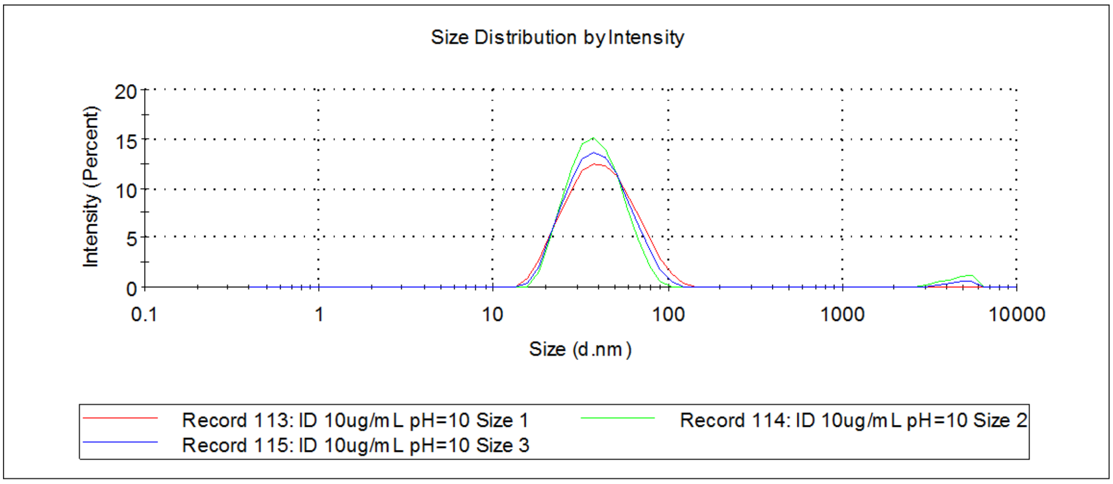


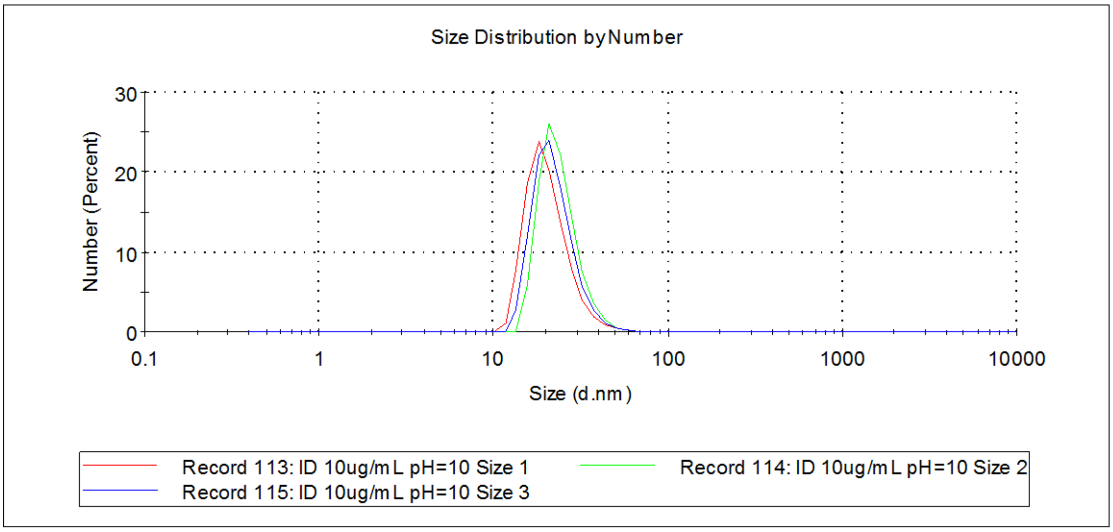


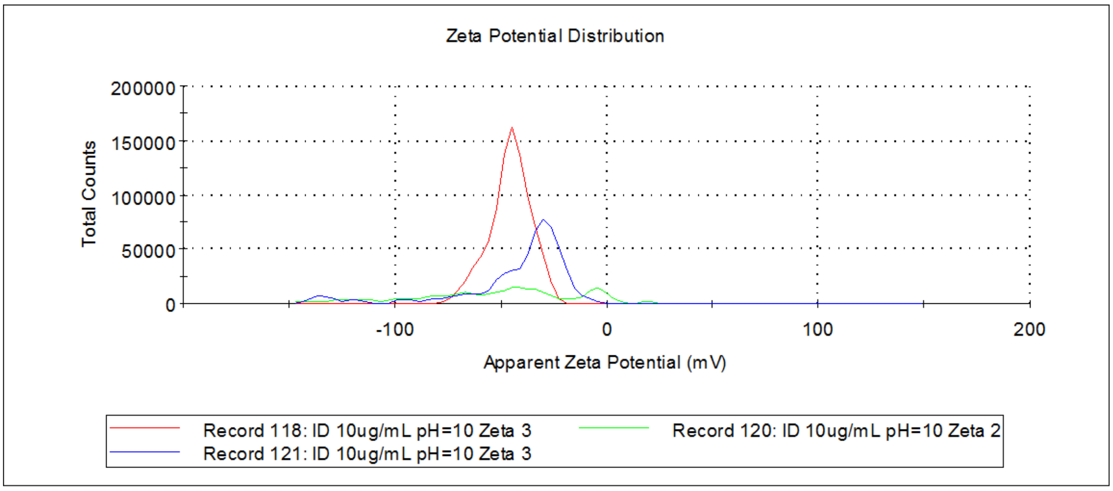

IONC-PAMAM pH 3:

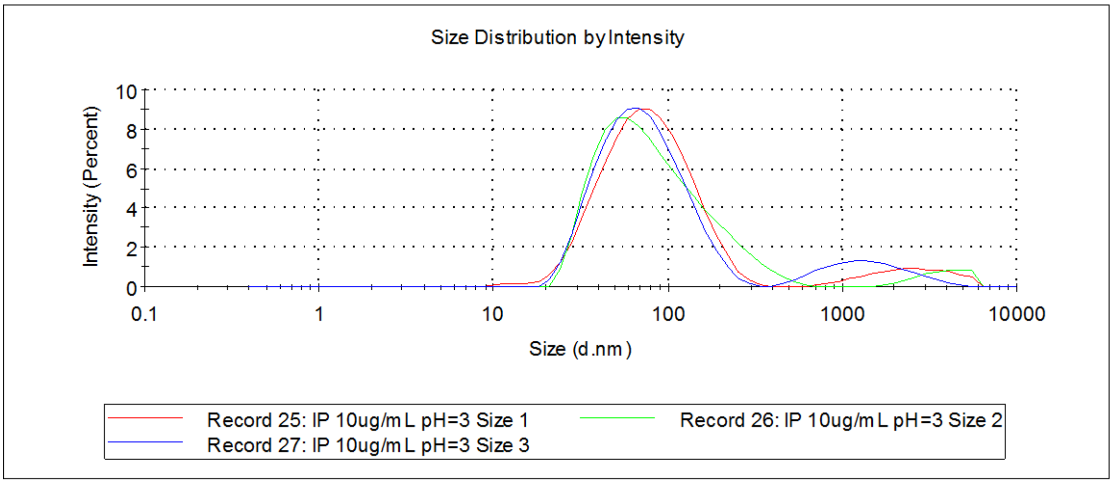


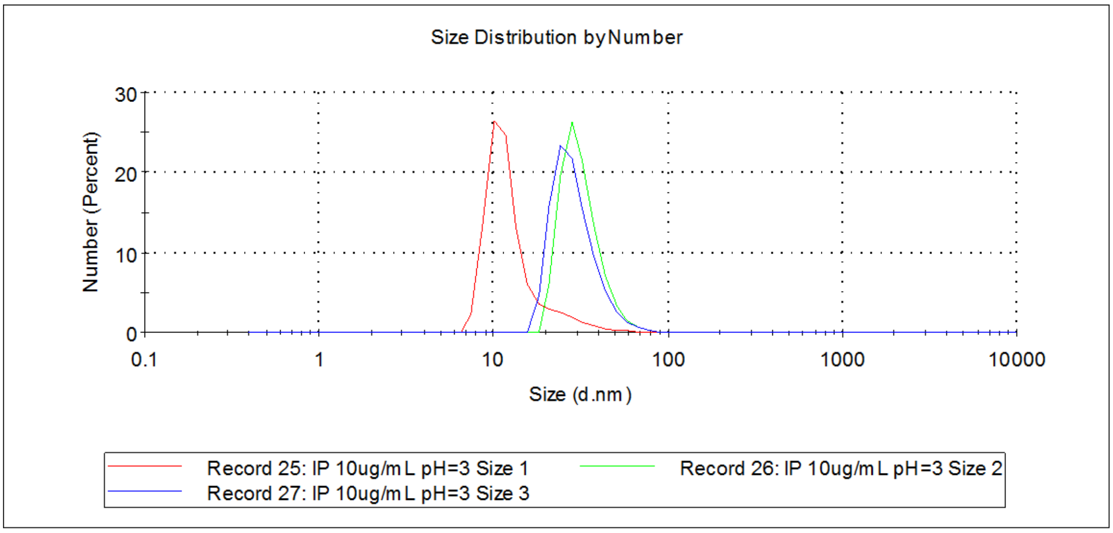


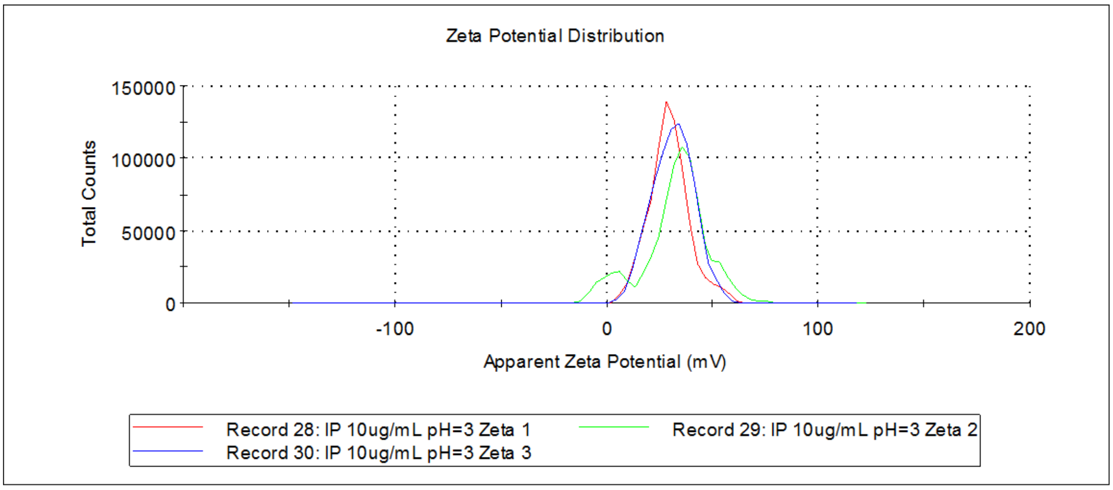

IONC-PAMAM pH 4:

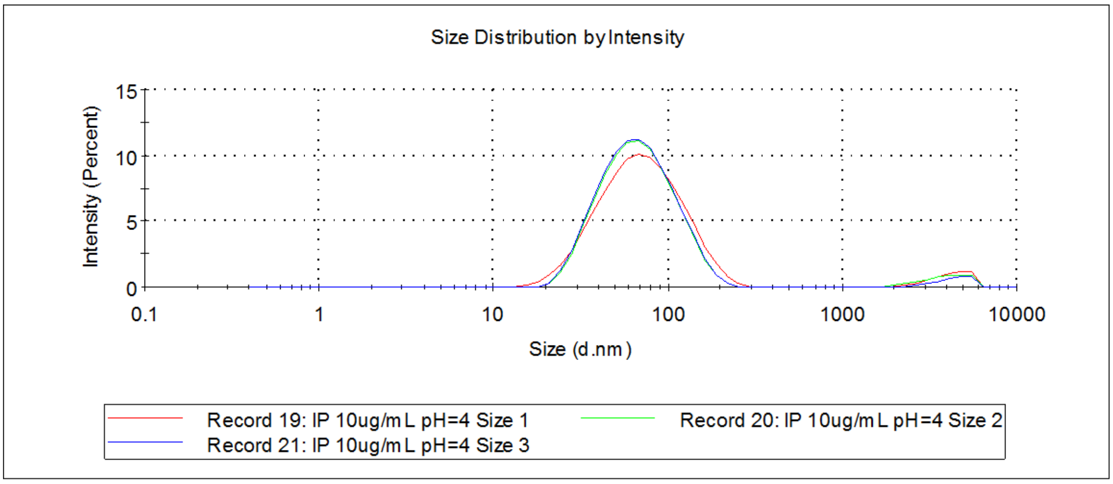


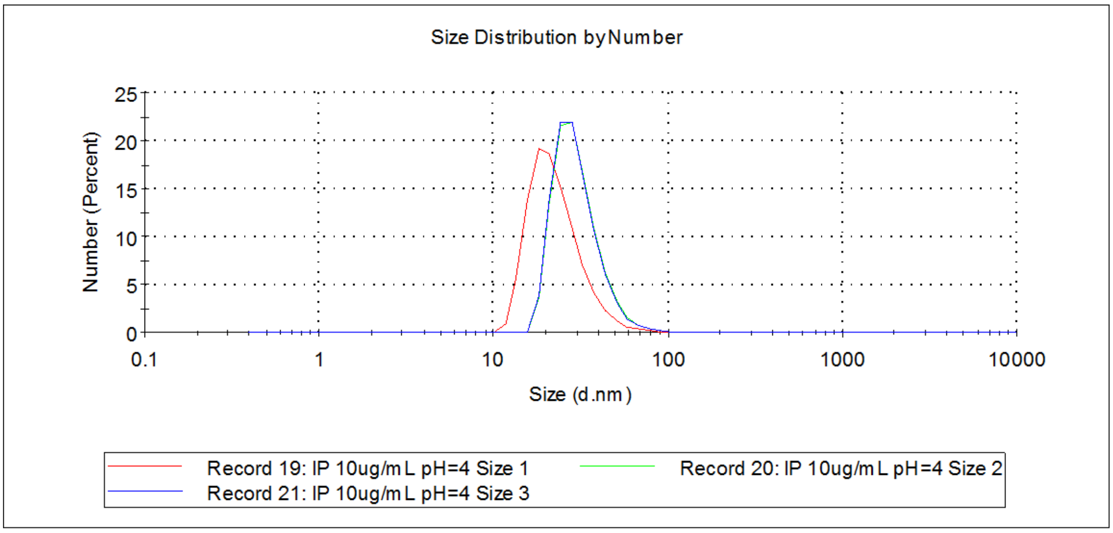


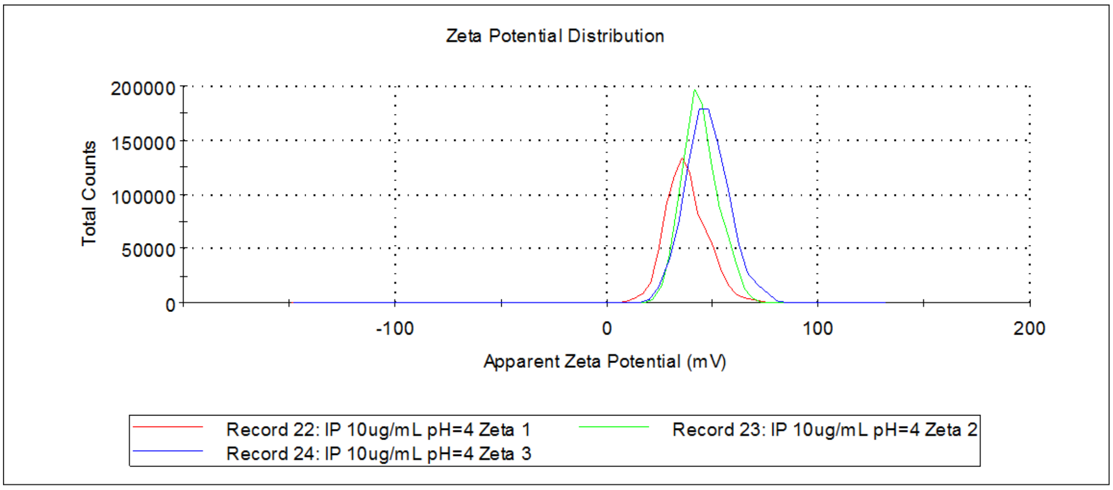

IONC-PAMAM pH 5:

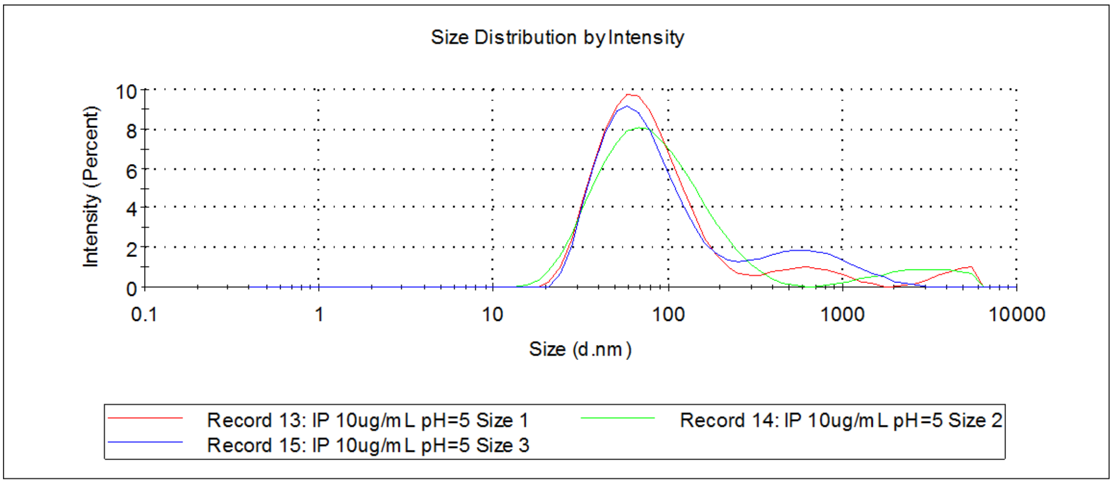


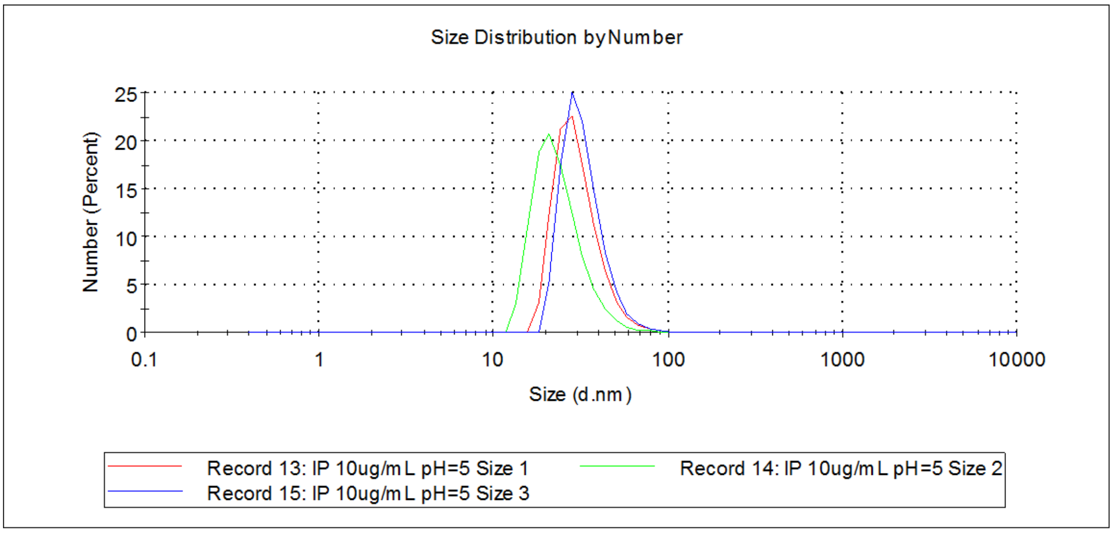


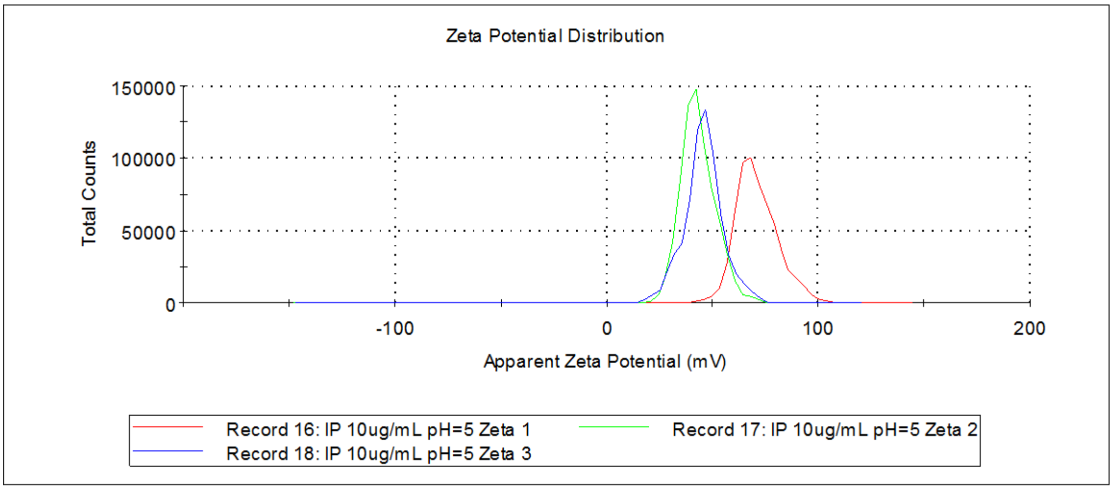

IONC-PAMAM pH 6:

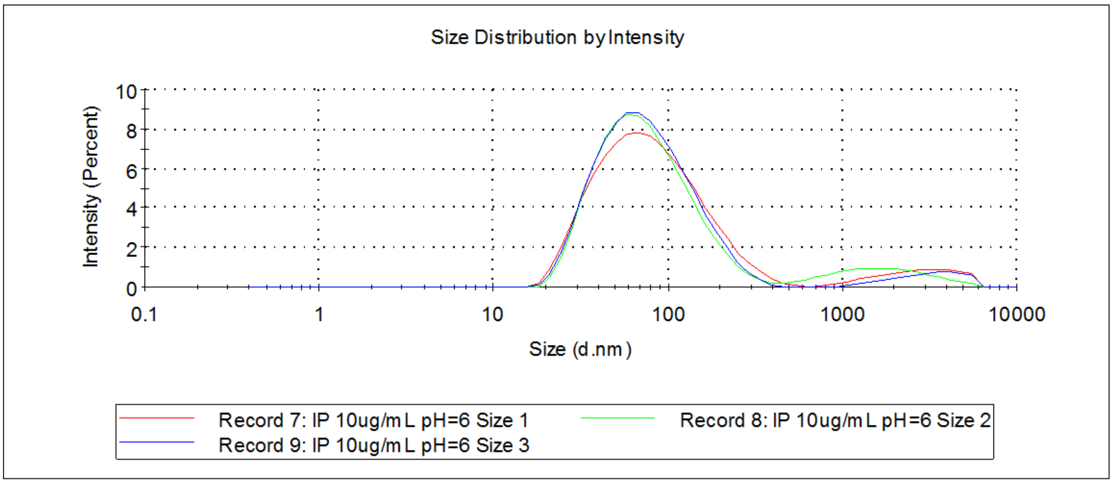


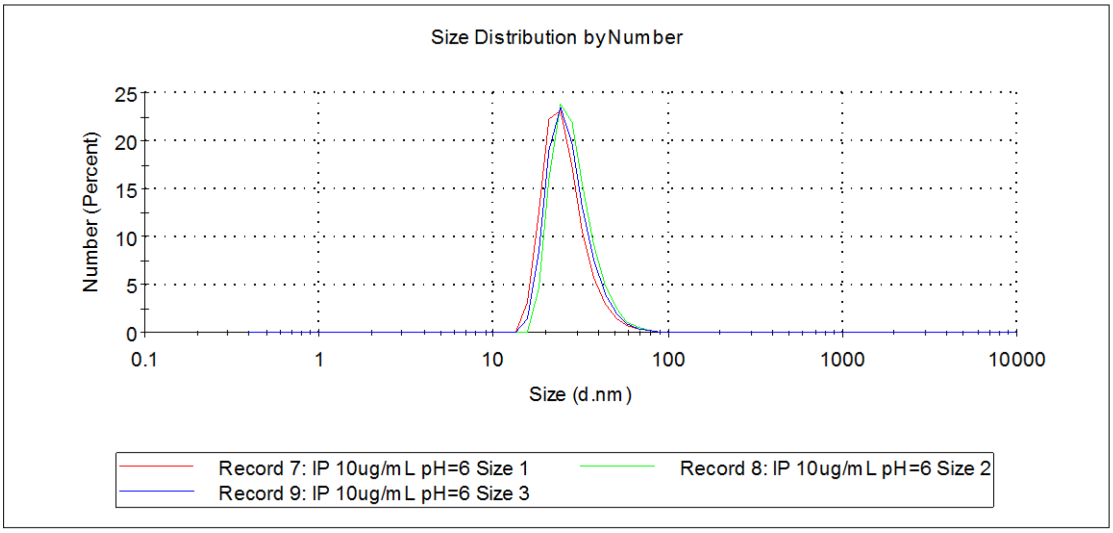


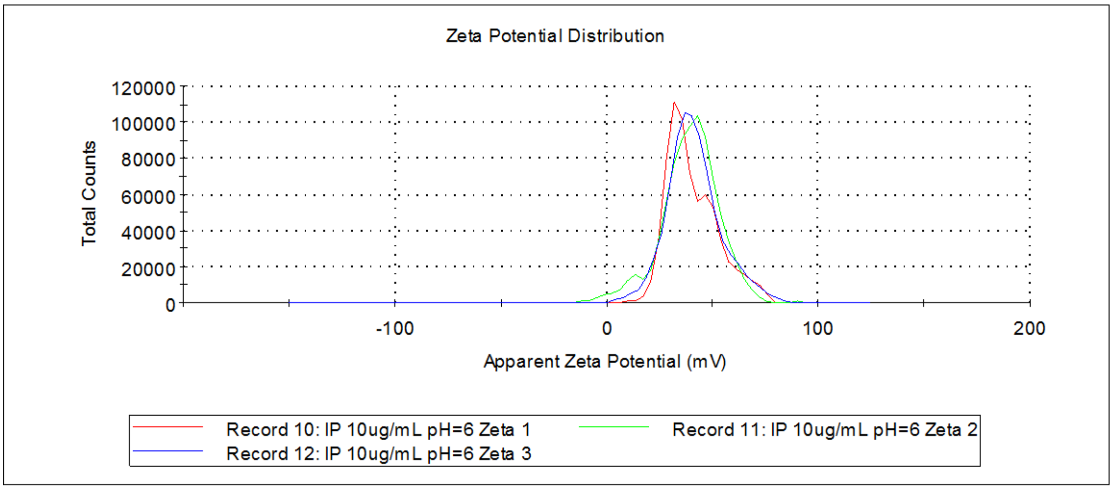

IONC-PAMAM pH 7:

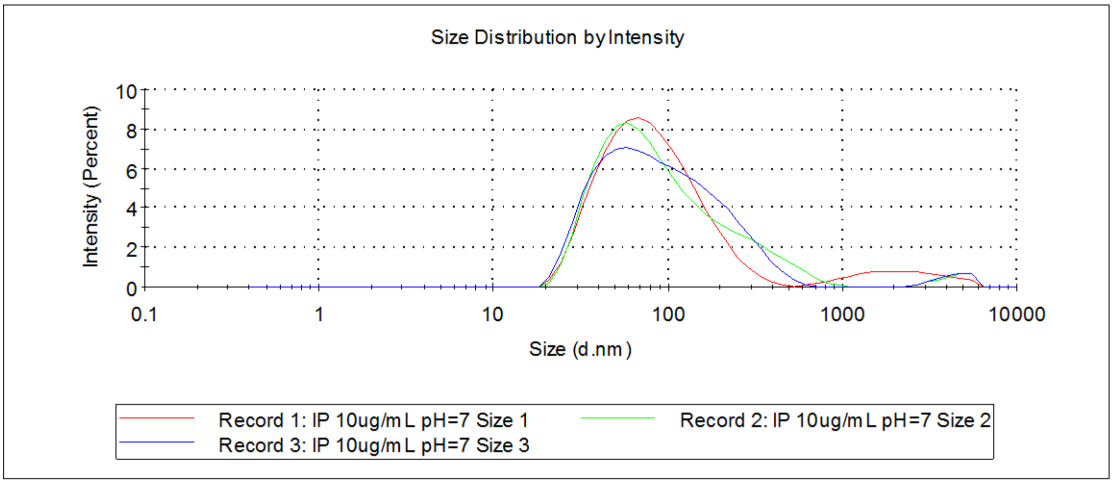


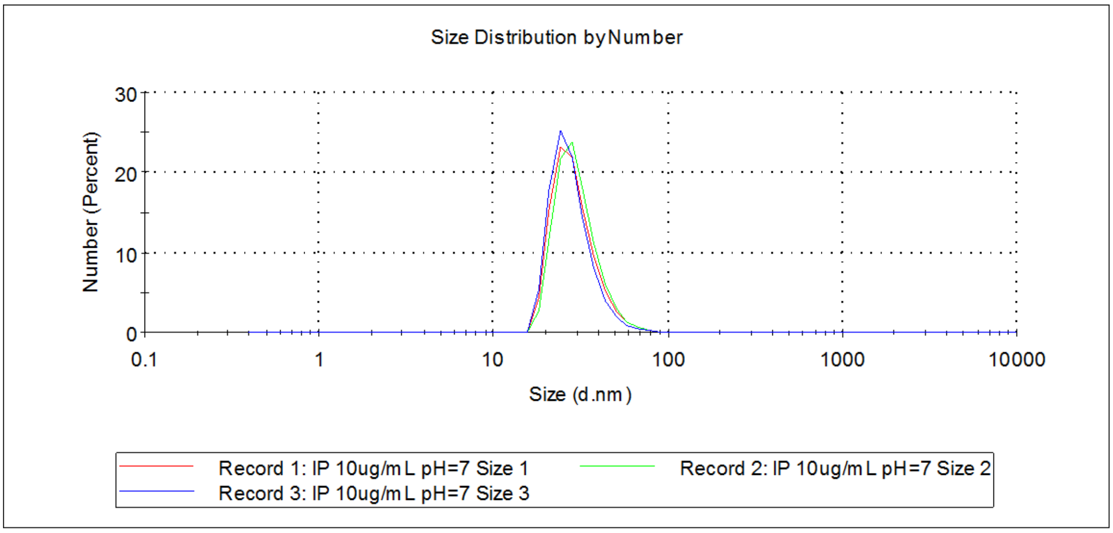


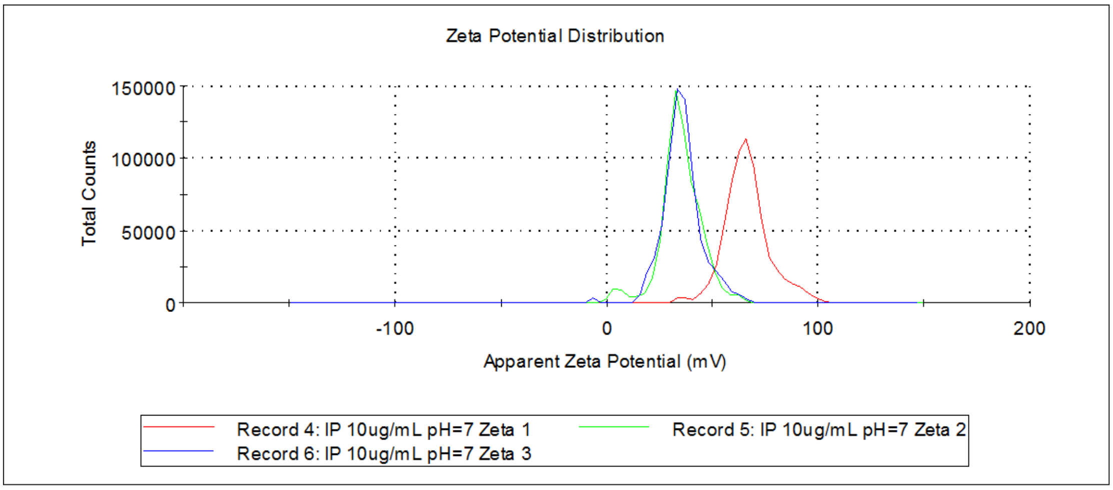

IONC-PAMAM pH 8:

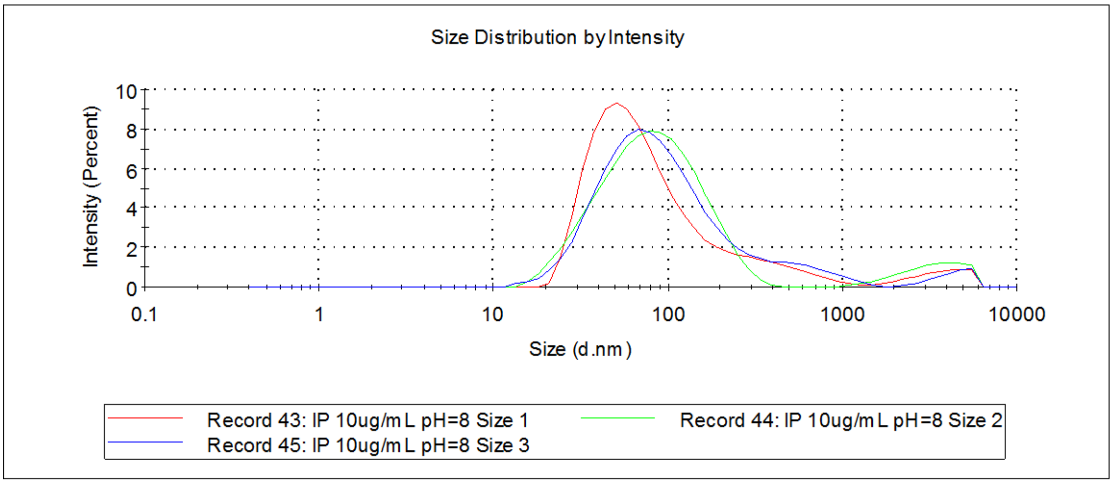


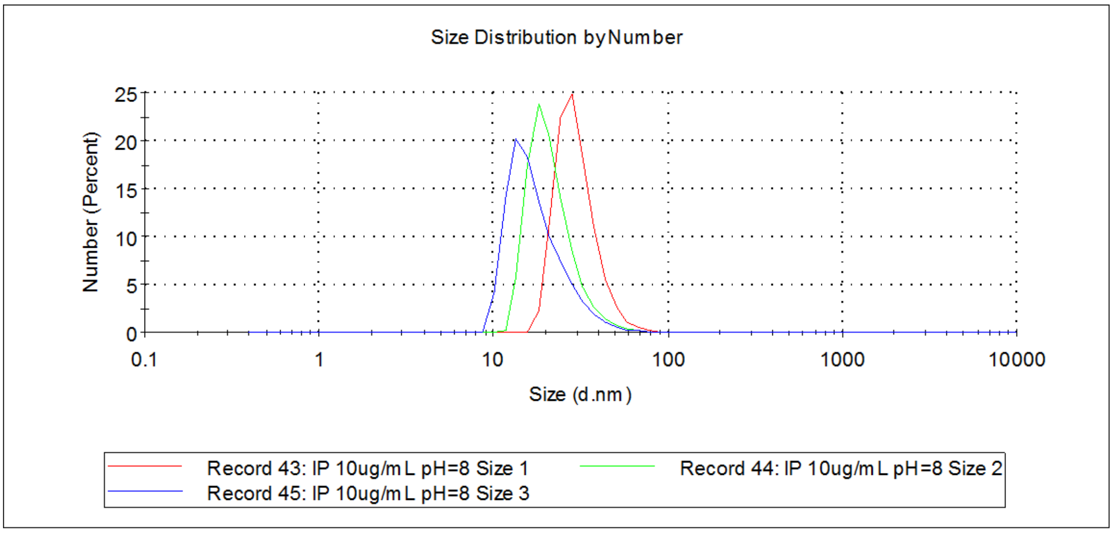


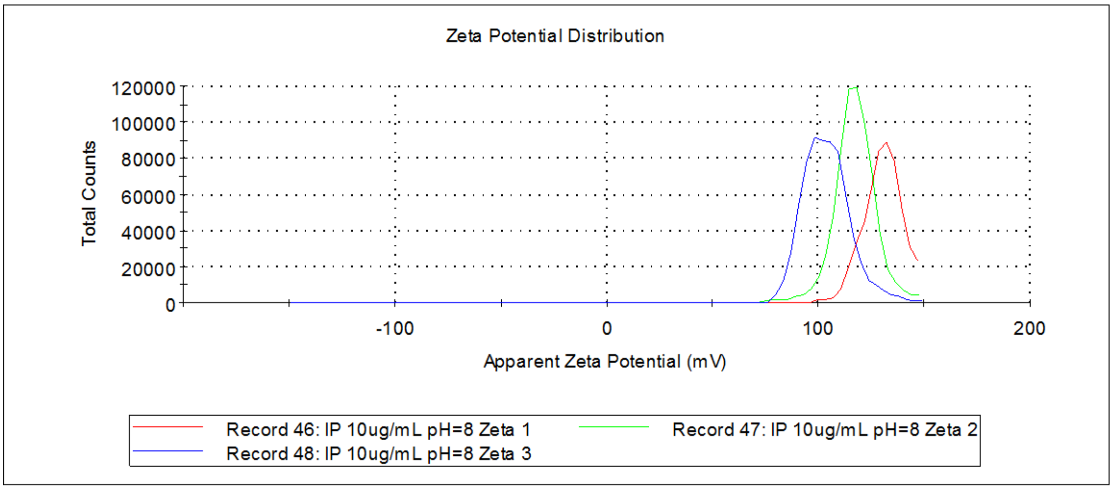

IONC-PAMAM pH 9:

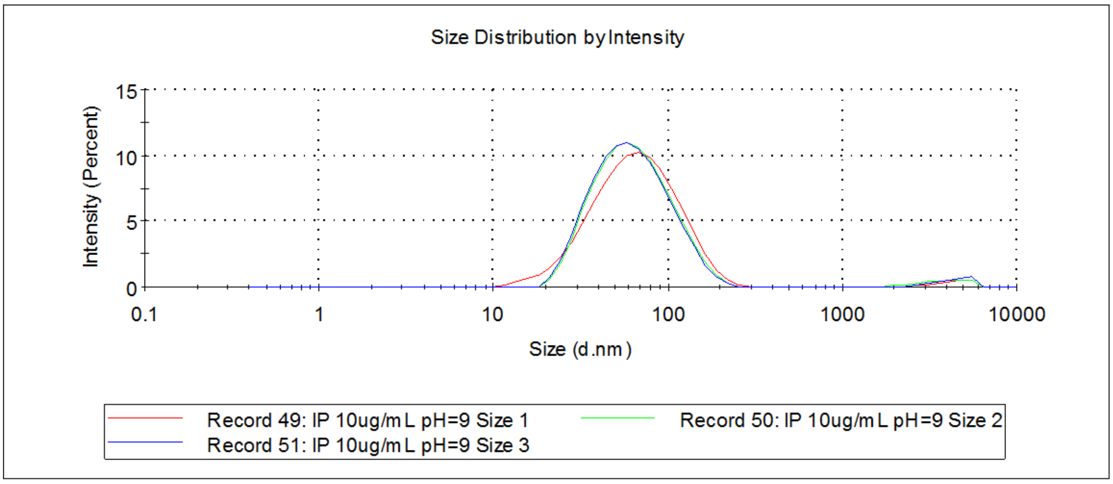


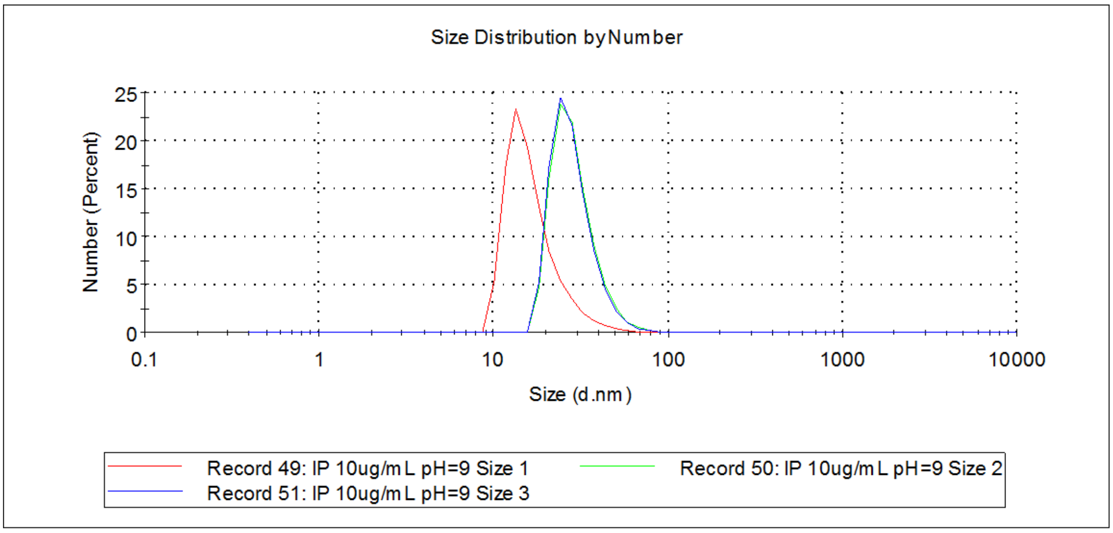


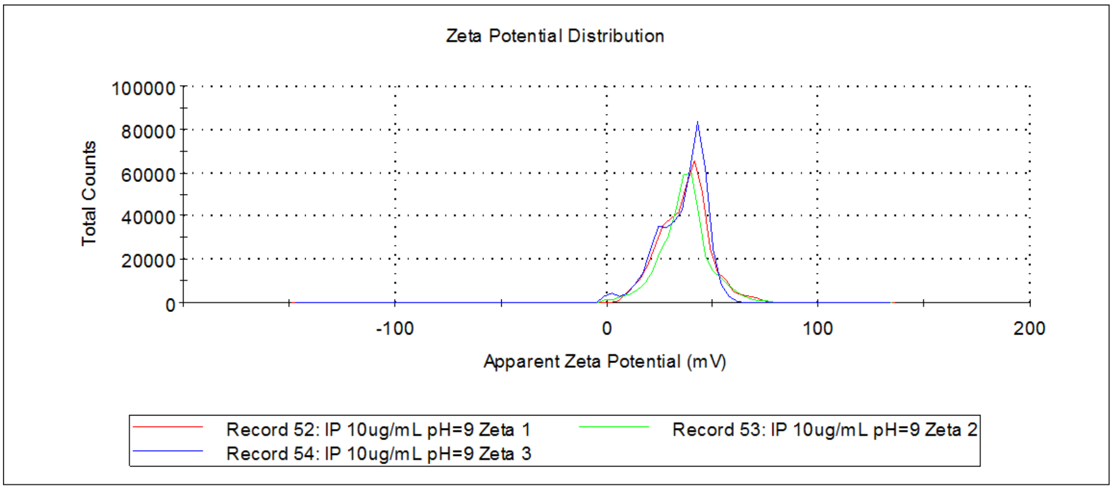

IONC-PAMAM pH 10:

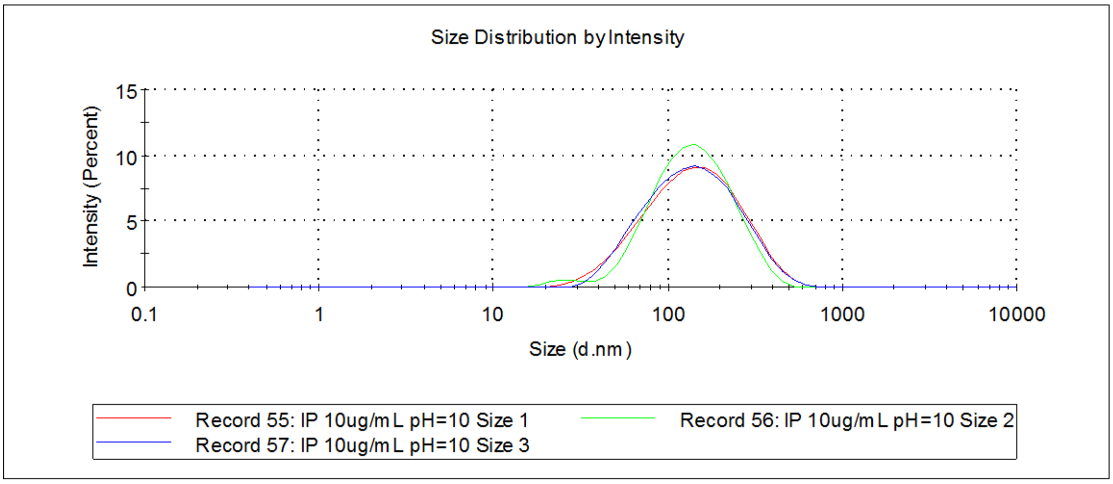


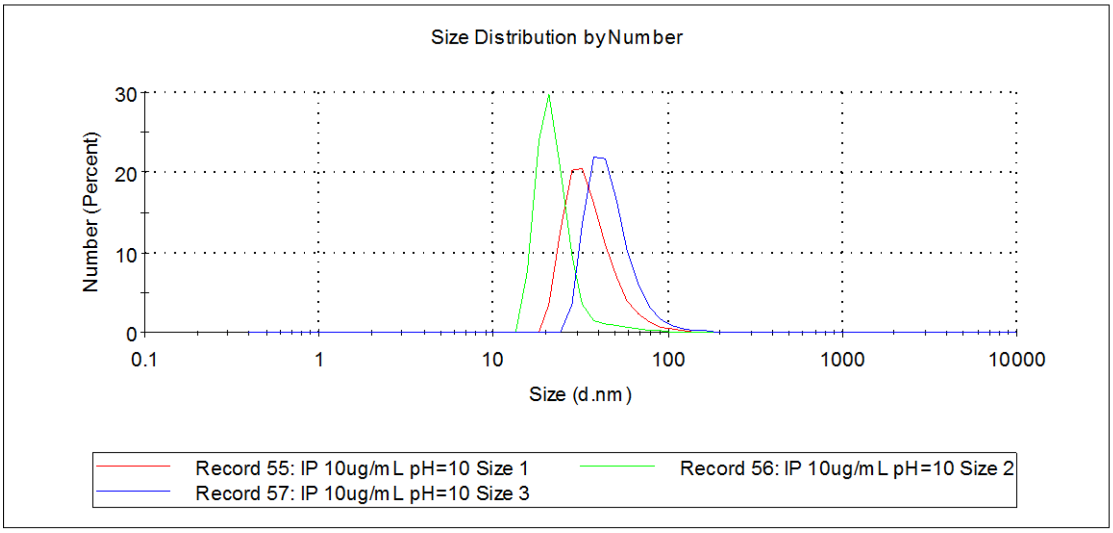


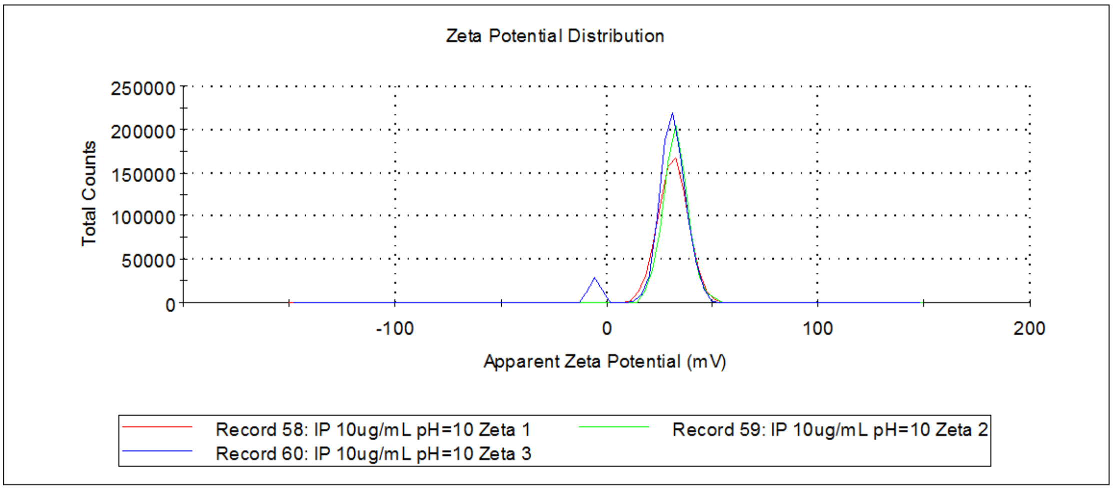

IONC-PAMAM-P123 pH 3:

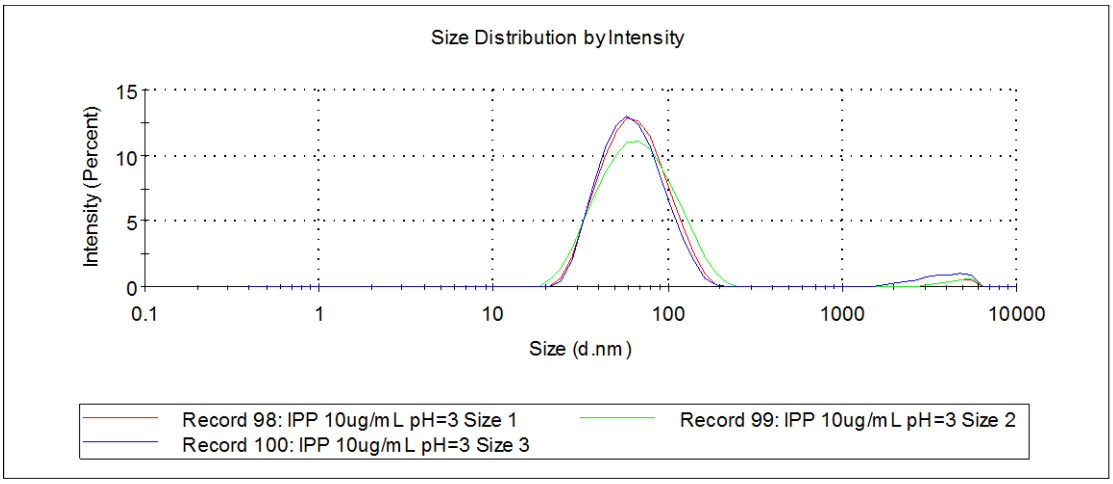


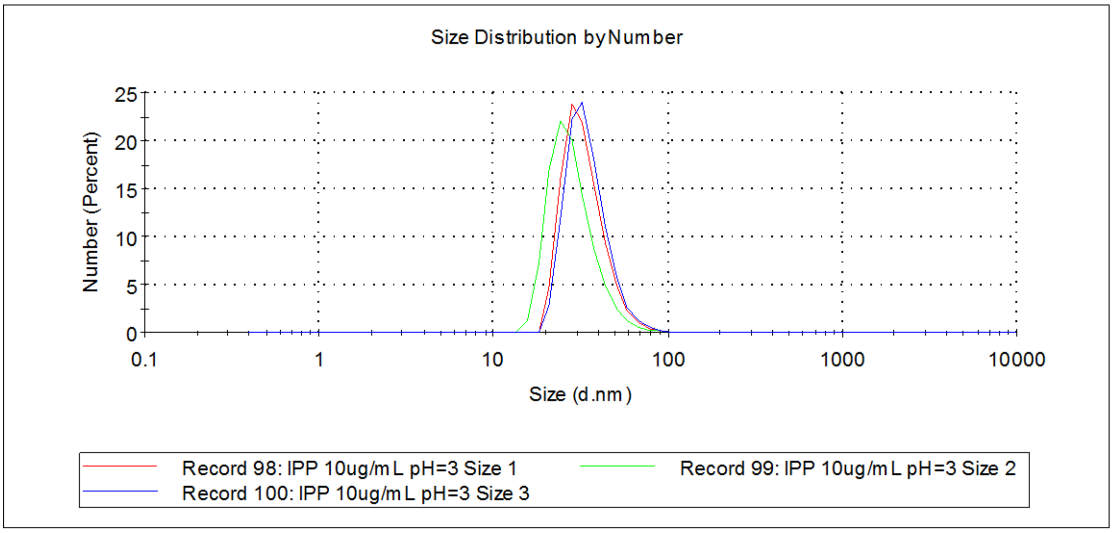


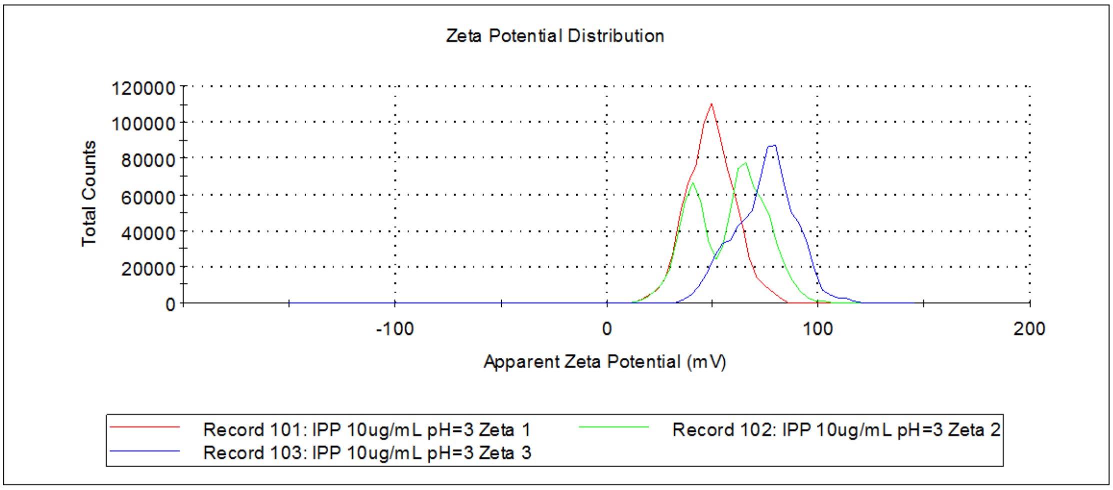

IONC-PAMAM-P123 pH 4:

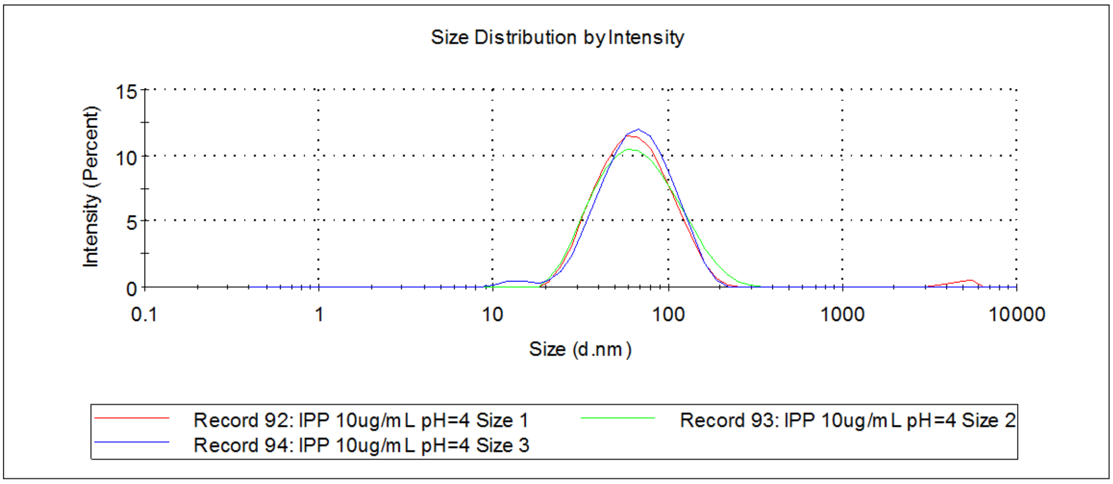


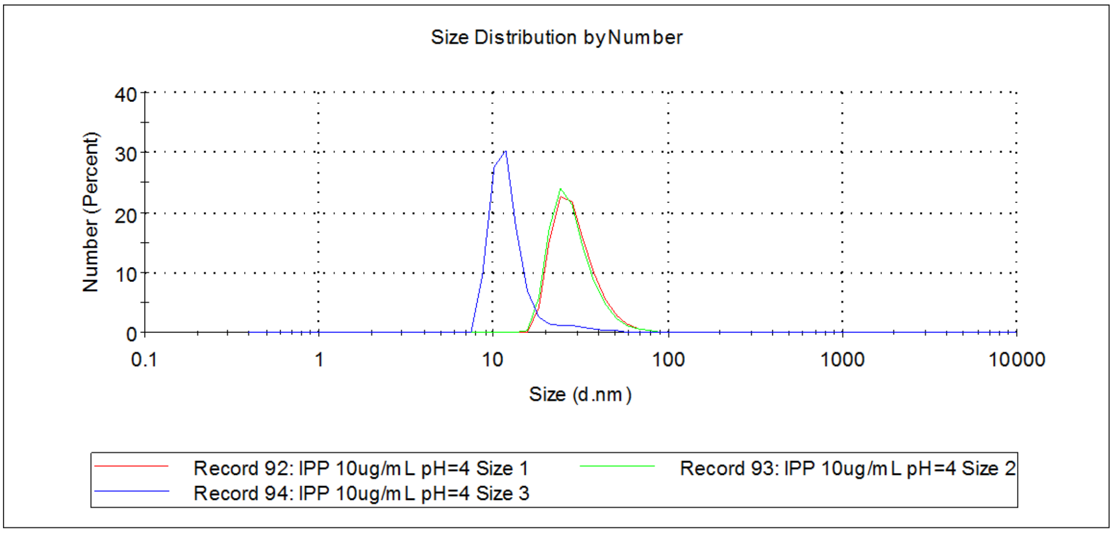


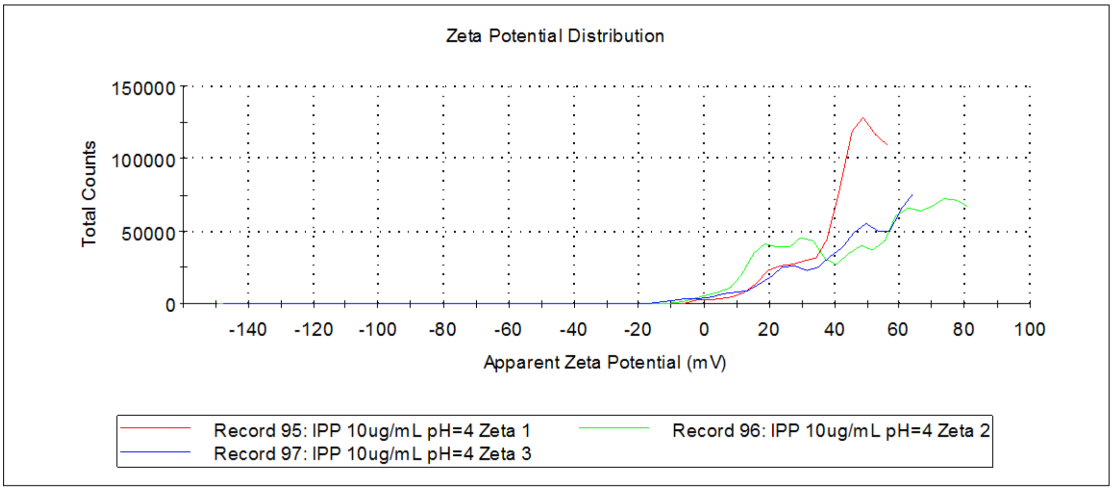

IONC-PAMAM-P123 pH 5:

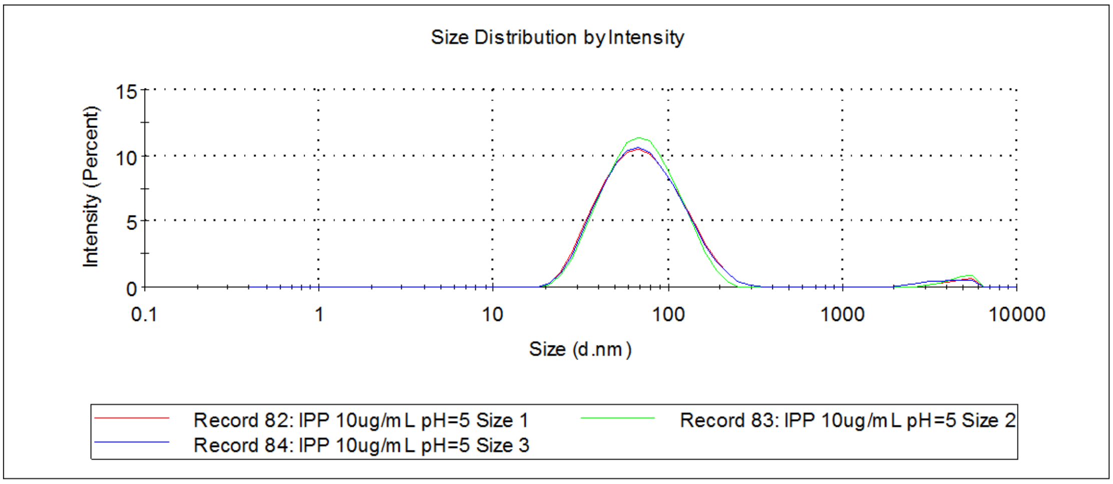


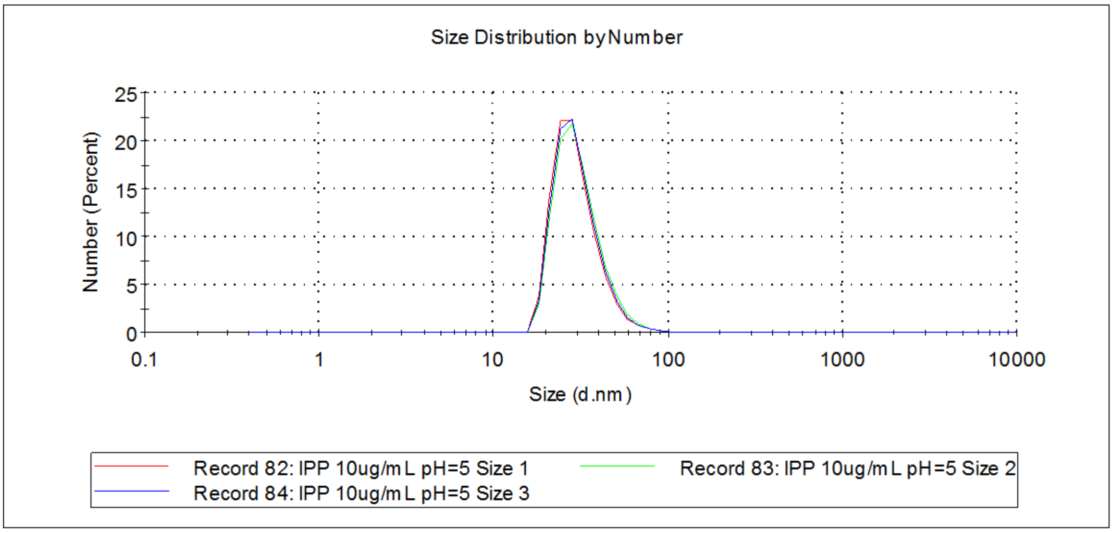


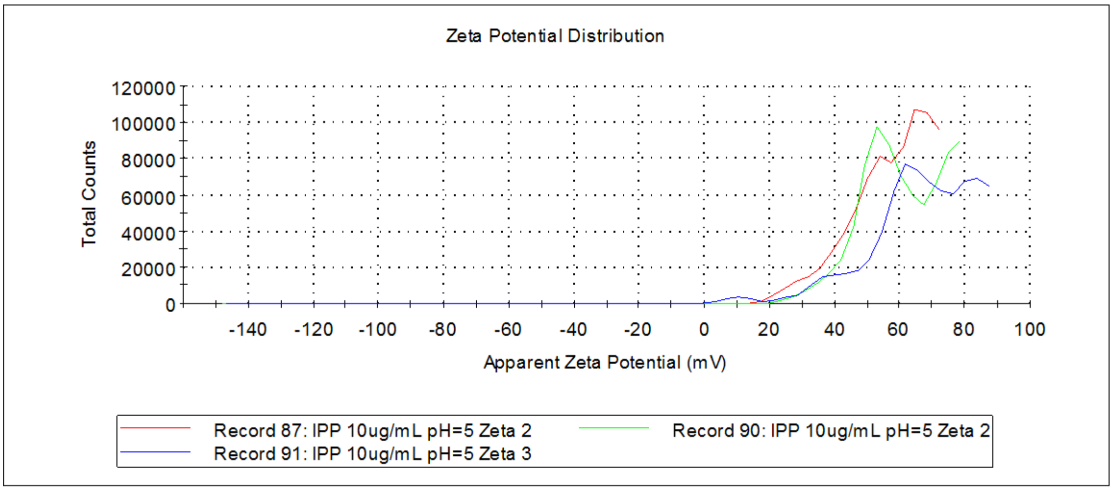

IONC-PAMAM-P123 pH 6:

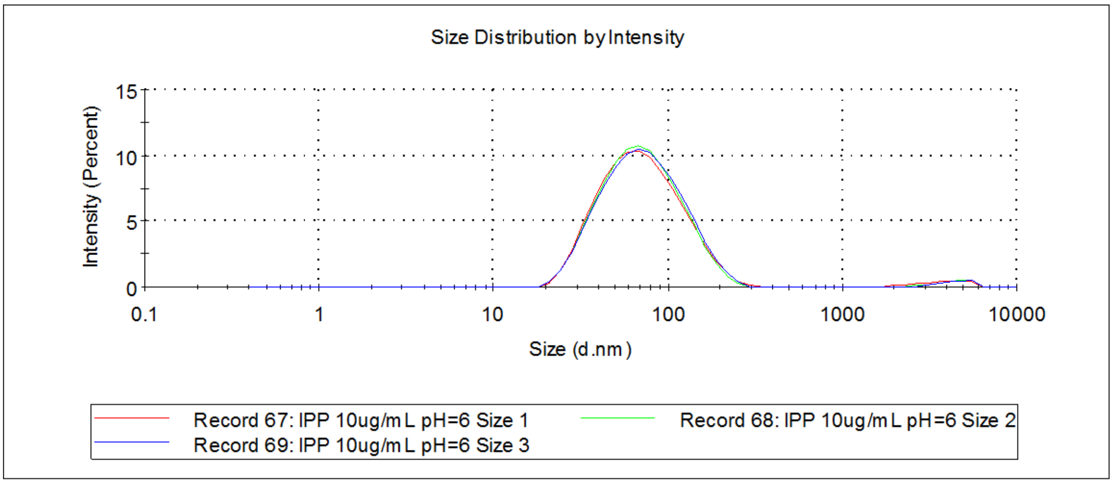


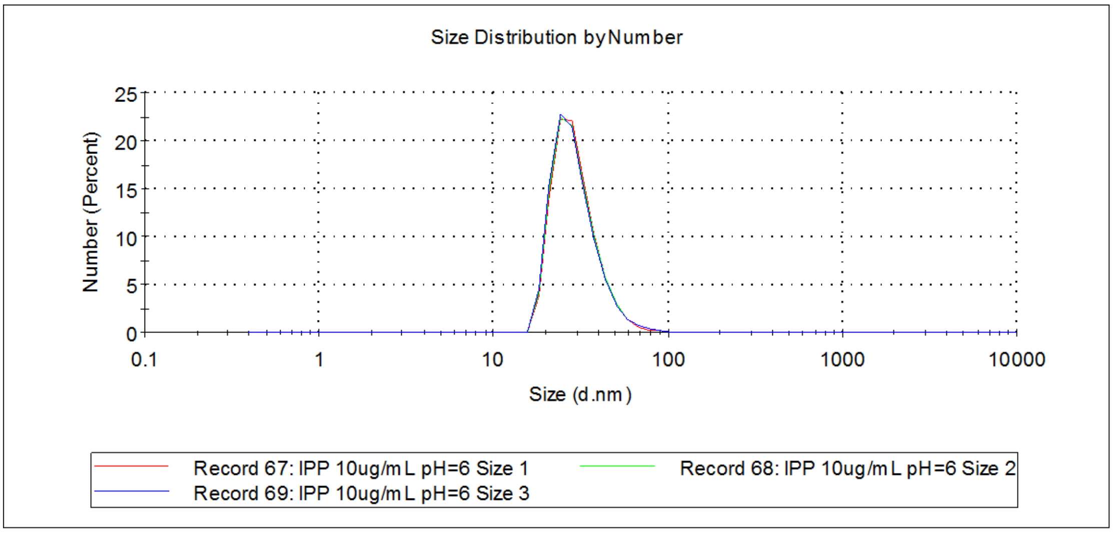


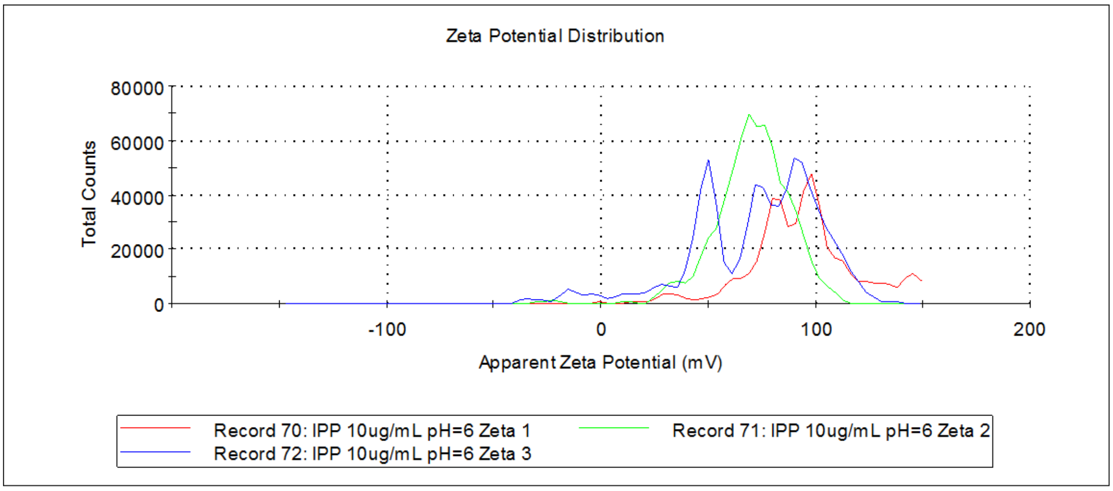

IONC-PAMAM-P123 pH 7:

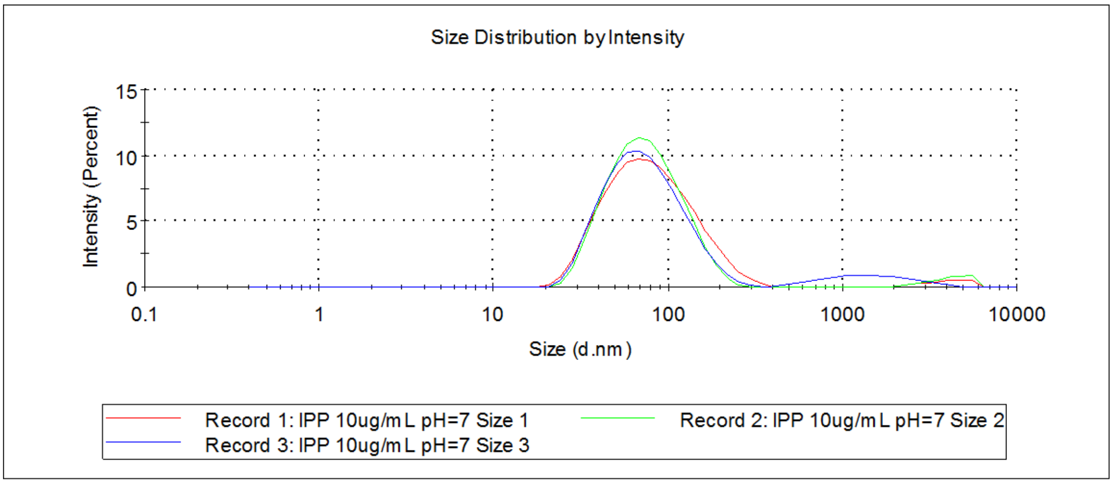


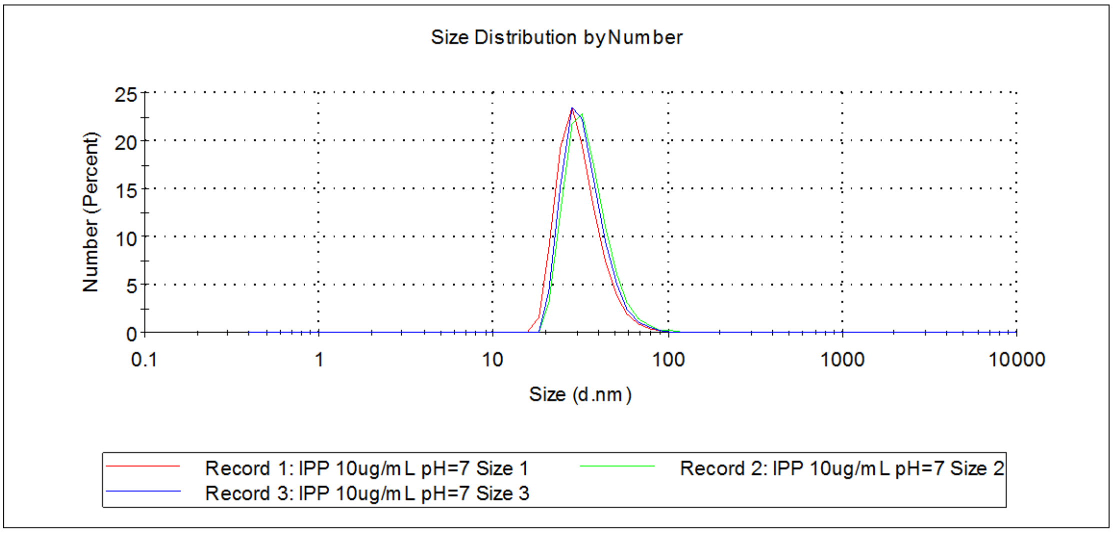


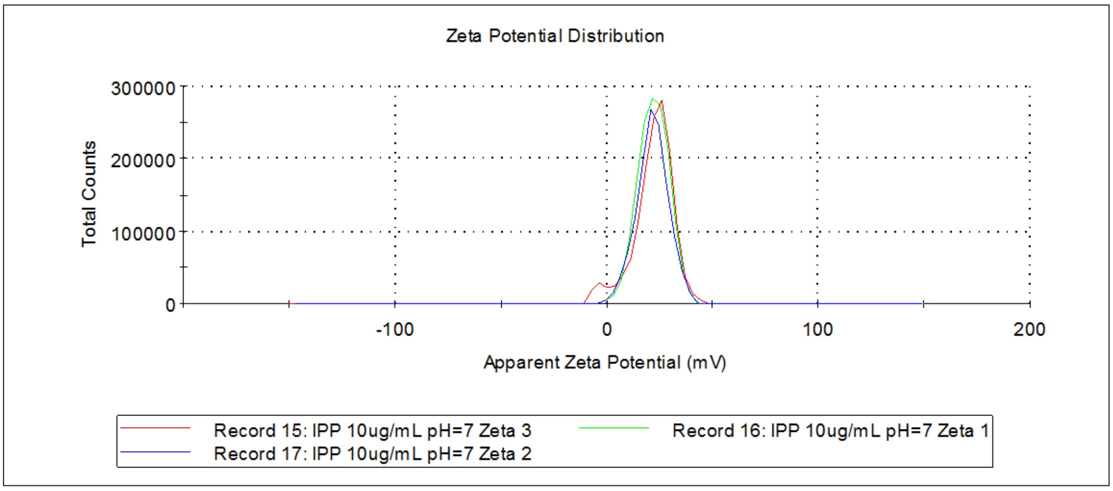

IONC-PAMAM-P123 pH 8:

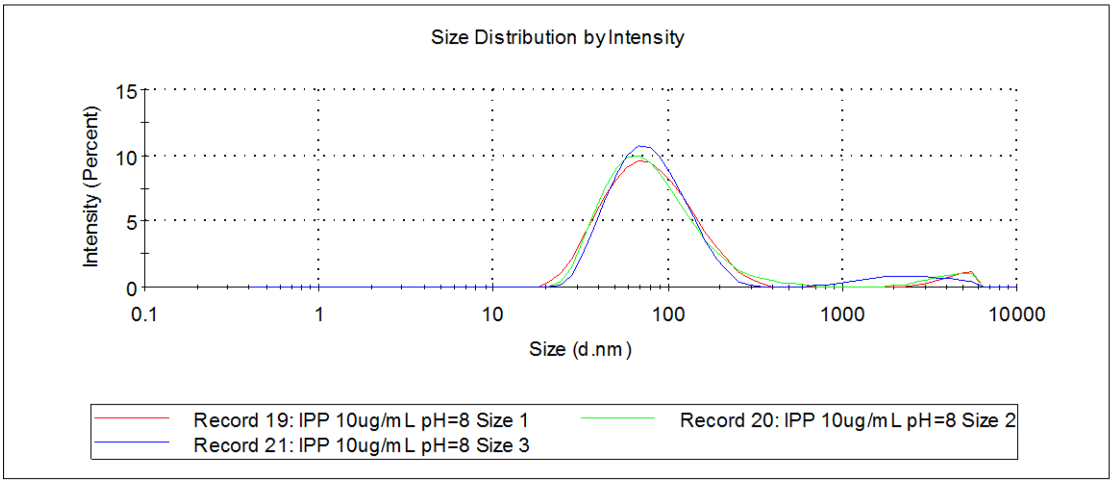


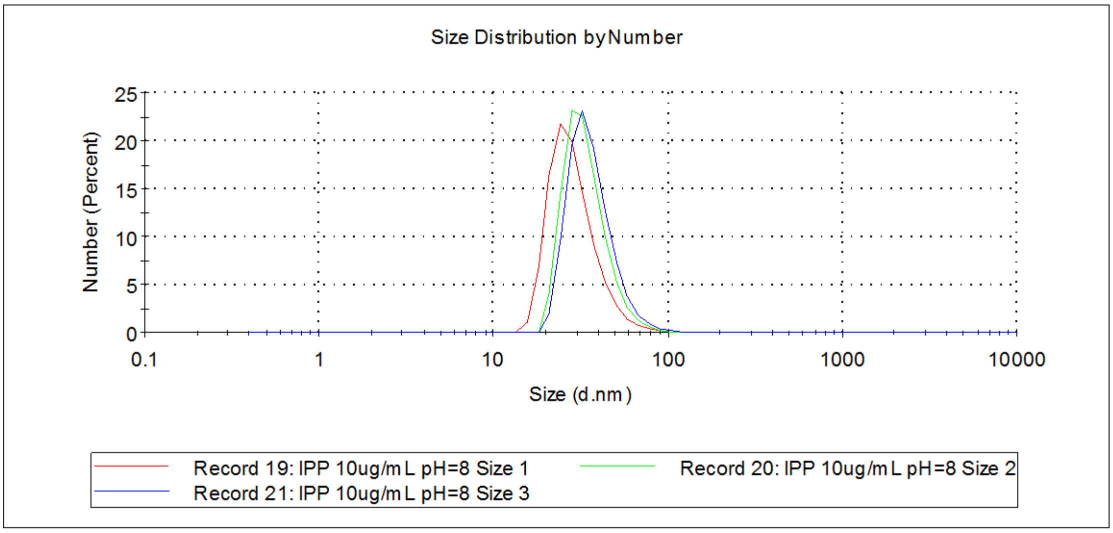


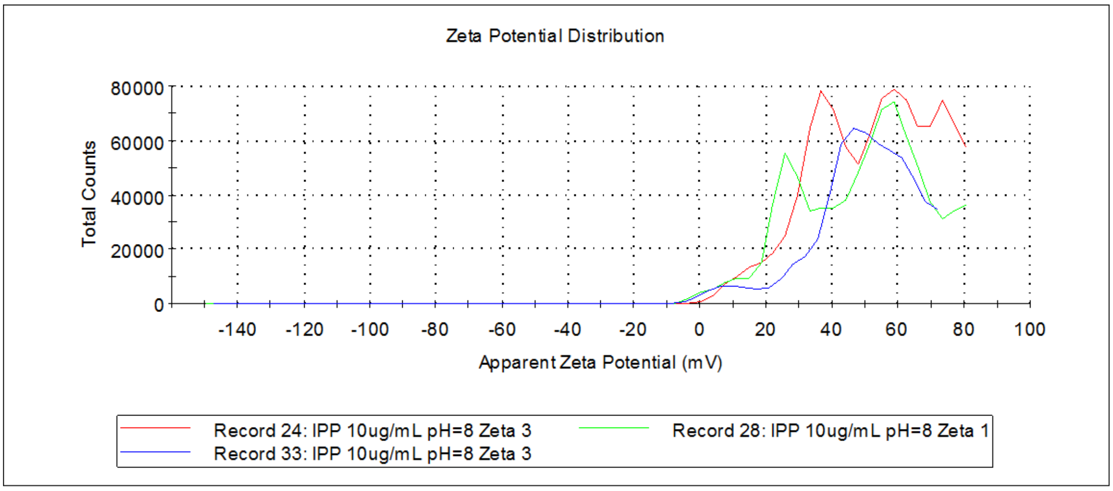

IONC-PAMAM-P123 pH 9:

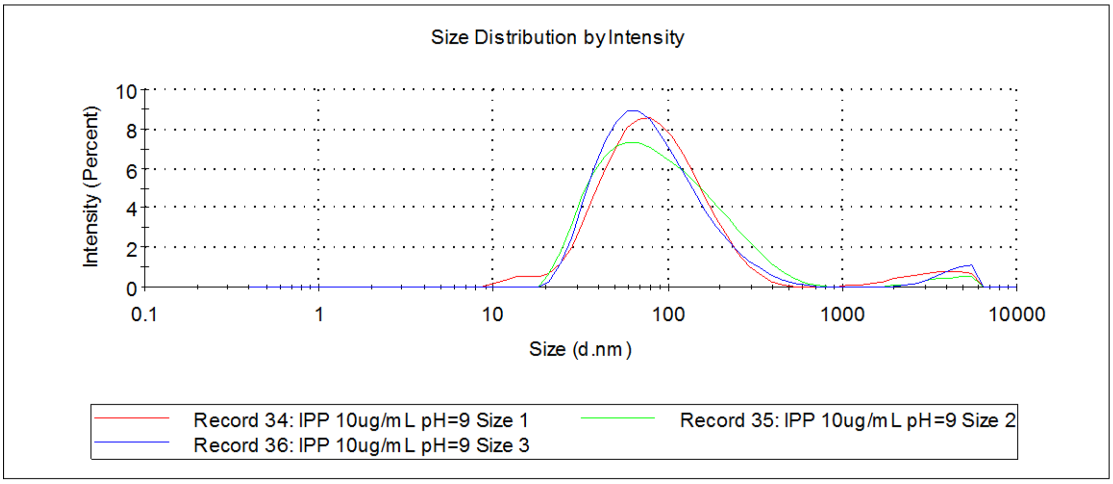


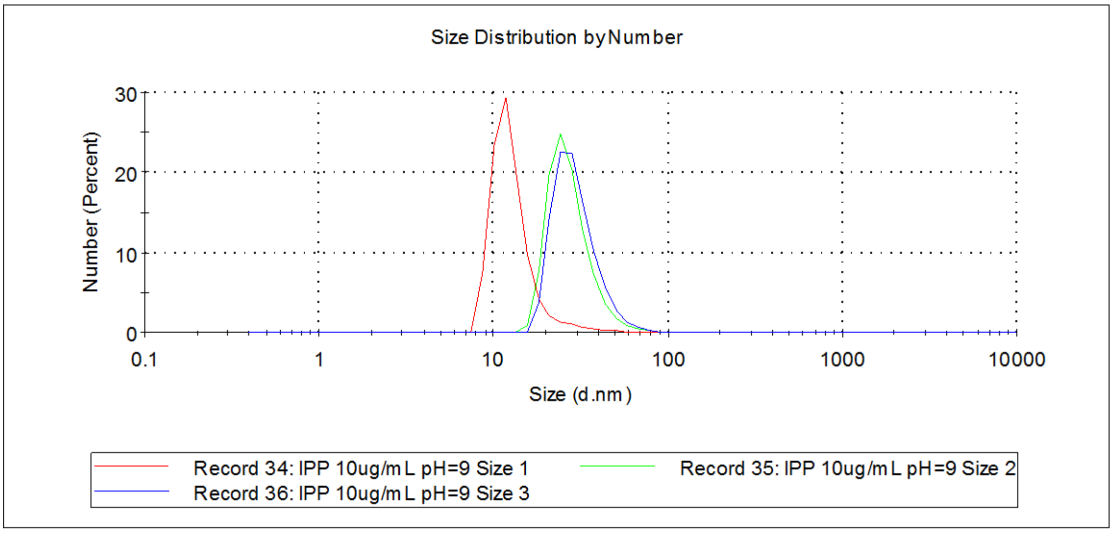


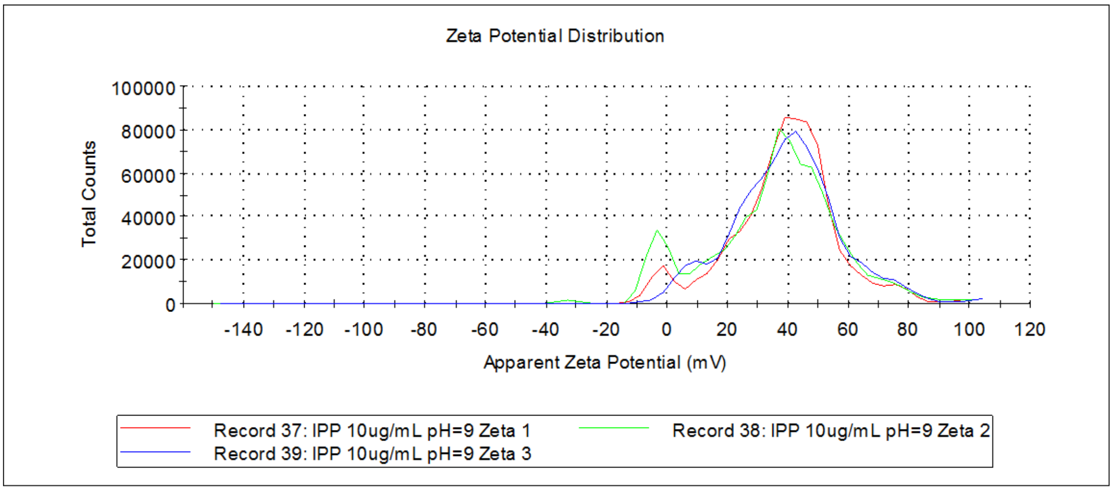

IONC-PAMAM-P123 pH 10:

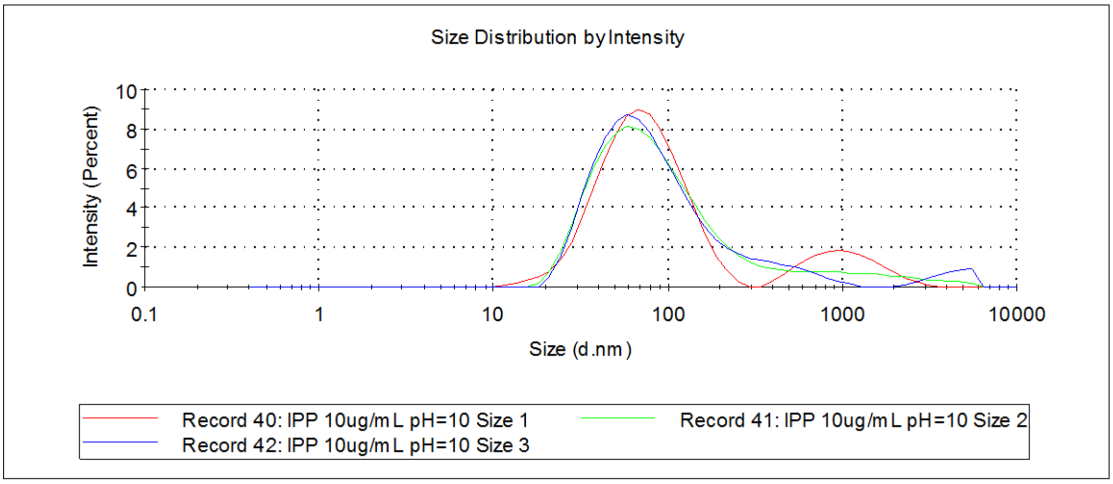


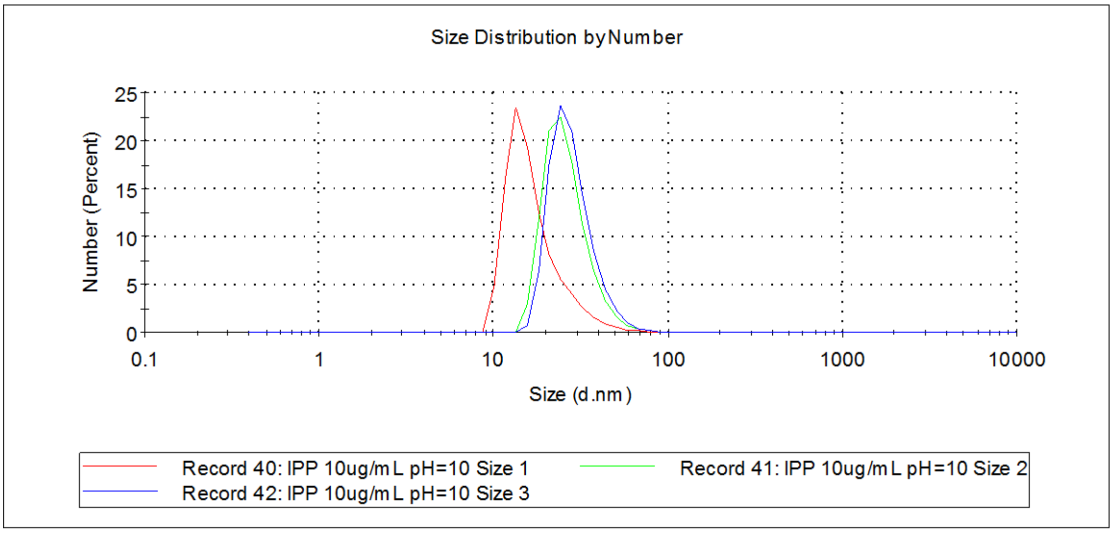


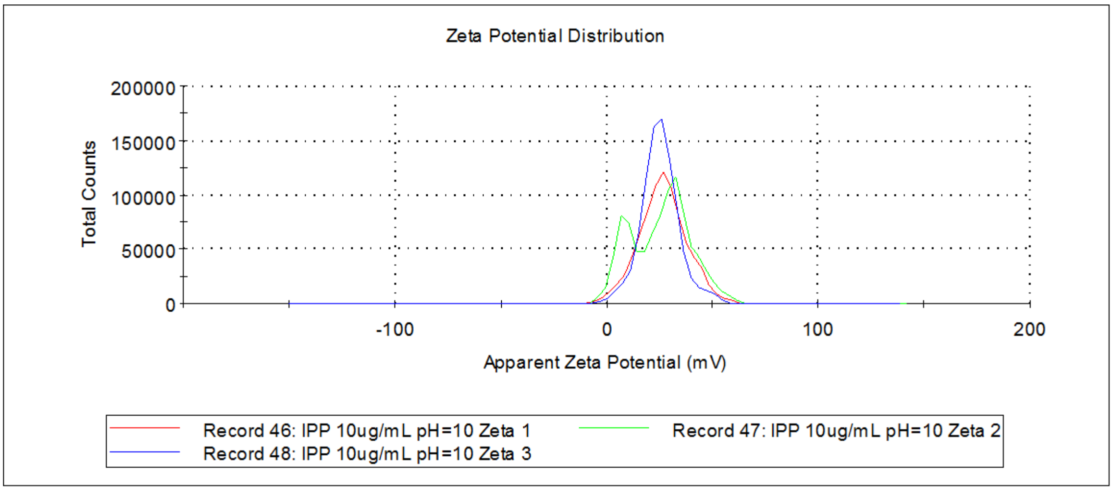

IPP/MB pH 7:

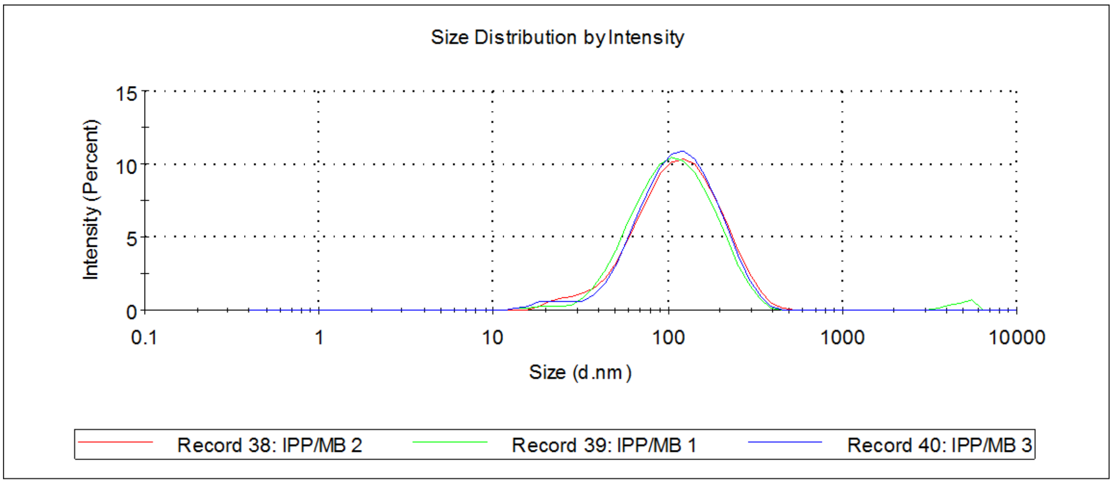
Size Distribution by Intensity
Intensity (Percent)
15
10
5
0
0.1
1
10
100
1000
10000
Size (d.nm)
Record 38: IPP/MB 2
Record 39: IPP/MB 1
Record 40: IPP/MB 3

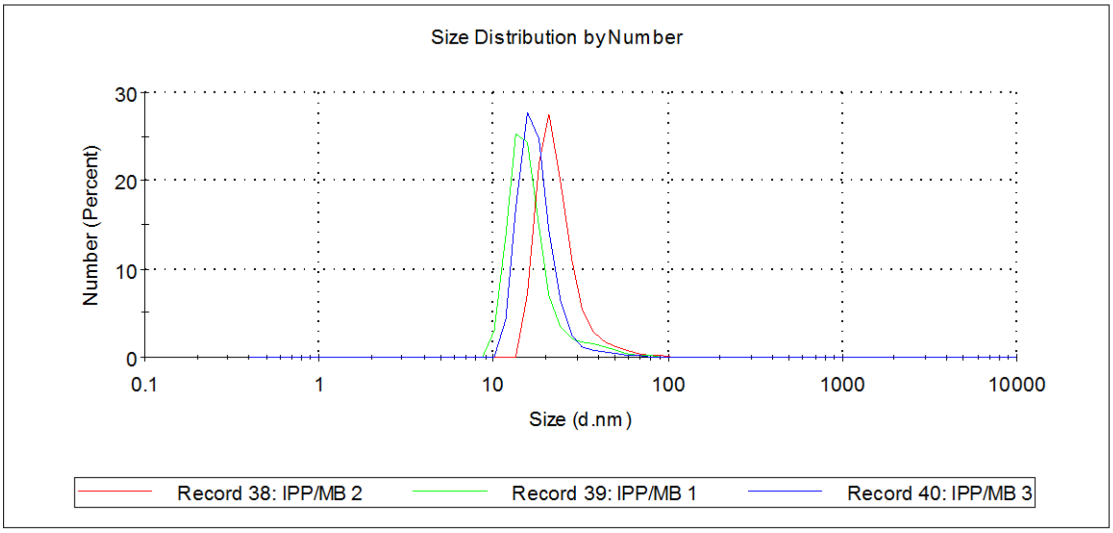
Size Distribution by Number
Number (Percent)
30
20
10
0
0.1
1
10
100
1000
10000
Size (d.nm)
Record 38: IPP/MB 2
Record 39: IPP/MB 1
Record 40: IPP/MB 3

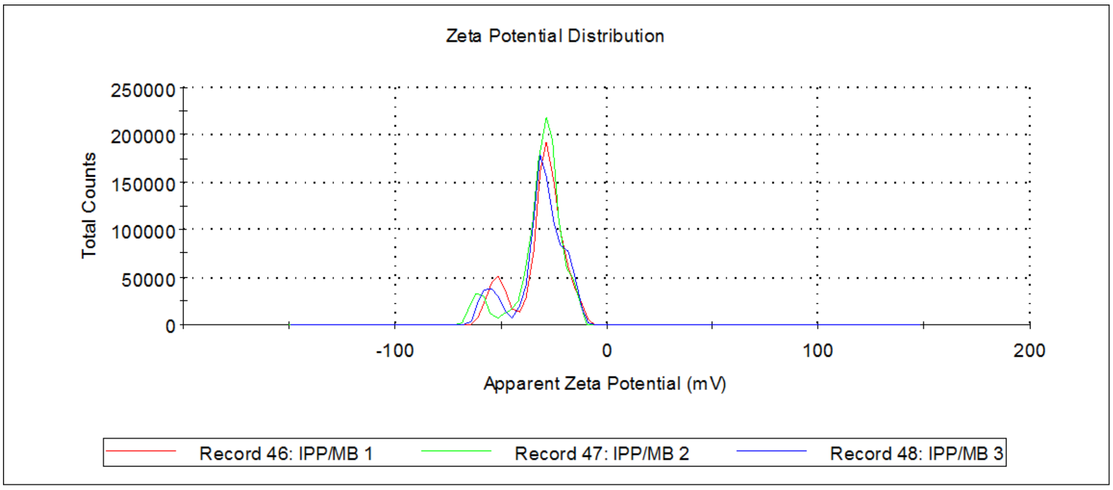
Zeta Potential Distribution
Total Counts
250000
200000
150000
100000
50000
0
-100
0
100
200
Apparent Zeta Potential (mV)
Record 46: IPP/MB 1
Record 47: IPP/MB 2
Record 48: IPP/MB 3
